# Magma recharge and mush rejuvenation drive paroxysmal activity at Stromboli volcano

**DOI:** 10.1038/s41467-022-35405-z

**Published:** 2022-12-13

**Authors:** Chiara Maria Petrone, Silvio Mollo, Ralf Gertisser, Yannick Buret, Piergiorgio Scarlato, Elisabetta Del Bello, Daniele Andronico, Ben Ellis, Alessio Pontesilli, Gianfilippo De Astis, Pier Paolo Giacomoni, Massimo Coltorti, Mark Reagan

**Affiliations:** 1 Natural History Museum, Volcano Petrology Group, Cromwell Road, SW7 5BD London, UK; 2 Department of Earth Sciences, Sapienza – University of Rome, P.Le Aldo Moro 5, 00185 Roma, Italy; 3 Istituto Nazionale di Geofisica e Vulcanologia – Sezione di Roma 1, Via di Vigna Murata 605, 00143 Roma, Italy; 4 School of Geography, Geology and the Environment, Keele University, Keele, Staffordshire, ST5 5BG UK; 5 Natural History Museum, Core Research Laboratory, Cromwell Road, SW7 5BD London, UK; 6 Istituto Nazionale di Geofisica e Vulcanologia, Osservatorio Etneo, Sezione di Catania, Piazza Roma 2, 95123 Catania, Italy; 7 Institute of Geochemistry and Petrology, ETH Zurich, 8092 Zurich, Switzerland; 8 Department of Earth Sciences, University of Pisa, Via Santa Maria 53, 56126 Pisa, Italy; 9 Department of Physics and Earth Sciences, University of Ferrara, Via Saragat 1, 44122 Ferrara, Italy; 10 Department of Earth and Environmental Sciences, University of Iowa, Iowa City, IA 52242 USA

**Keywords:** Natural hazards, Petrology, Volcanology, Mineralogy

## Abstract

Open-conduit basaltic volcanoes can be characterised by sudden large explosive events (paroxysms) that interrupt normal effusive and mild explosive activity. In June-August 2019, one major explosion and two paroxysms occurred at Stromboli volcano (Italy) within only 64 days. Here, via a multifaceted approach using clinopyroxene, we show arrival of mafic recharges up to a few days before the onset of these events and their effects on the eruption pattern at Stromboli, as a prime example of a persistently active, open-conduit basaltic volcano. Our data indicate a rejuvenated Stromboli plumbing system where the extant crystal mush is efficiently permeated by recharge magmas with minimum remobilisation promoting a direct linkage between the deeper and the shallow reservoirs that sustains the currently observed larger variability of eruptive behaviour. Our approach provides vital insights into magma dynamics and their effects on monitoring signals demonstrating the power of petrological studies in interpreting patterns of surficial activity.

## Abstract (web summary)

Petrological studies along with volcano monitoring data relate the unusual 2019 explosive activity at Stromboli volcano (Italy) to deep magma recharges up to a few days prior the eruption and a direct link between deep and shallow magma reservoirs.

## Introduction

Basaltic volcanoes can remain active for tens to thousands of years with the continual presence of magma^1^, requiring storage and transport conditions that can sustain persistently eruptible melt. Magma storage conditions beneath these volcanoes may significantly change with time, leading to sudden and dramatic changes in explosivity^2,3^. Determining the rates and causes of these changes and how they modulate eruptive style over societally relevant timescales is of paramount importance for evaluating potential hazards. The three large-scale explosions (one major explosion and two paroxysms) that occurred at Stromboli volcano (Italy) over an uncommonly short time interval between 25 June and 28 August 2019 offer a unique opportunity to study the short-term variations in a basaltic plumbing system that can lead to paroxysmal events.

**Fig. 1:**
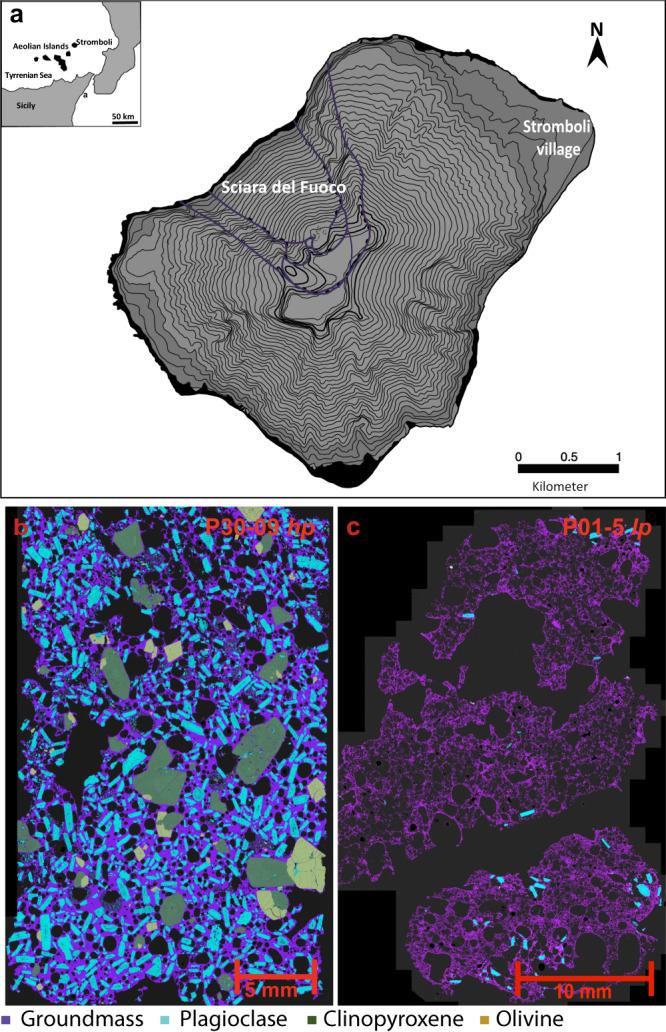
Map of Stromboli volcano and mineralogical map of 2019 tephra. **a** Map of Stromboli volcano with its location in respect to Southern Italy shown in the inset. Mineralogical maps with colour-coded mineral phases of 2019 *hp* (**b**) and *lp* (**c**) tephra from the 3 July (samples P30-09 *hp* and P01-5 *lp*) paroxysmal events. Typical *hp* black scoriae with high-porphyritic seriate textures (~45–50 vol%), low vesicularity (~10–20 vol.%) and microlite-rich glassy groundmass have erupted during normal Strombolian activity, major explosions, and paroxysmal events. Light *lp* pumices characterised by weakly porphyritic seriate textures (<10 vol.% phenocrysts and microphenocrysts), high vesicularity (~30–40 vol.%) and a glassy groundmass are erupted during paroxysmal events and less frequently during major explosions. Phenocrysts and microphenocrysts of plagioclase (~20–30 vol.% of the total crystal content), clinopyroxene (~10–15 vol.%) and minor olivine (≤5 vol.%) are present in both *hp* and *lp* tephra. However, the larger plagioclase phenocrysts visible in the *lp* pumice in **c** (cyan crystals), are derived by exchange from *hp-*magma.

Stromboli is an active open conduit basaltic volcano (Fig. 1a) well-known for its persistent mild (normal) Strombolian activity occasionally interrupted by sudden, short-lived events ranging in size and intensity from major (violent Strombolian) to paroxysmal explosions^4,5^. Four paroxysms occurred at Stromboli over the last 70 years of recorded eruptive history prior to 2019. Each was preceded by week- to month-long periods of effusive activity^6,7^. Notably, the 2003 and 2007 paroxysms followed eruption of a similar cumulative volume of lava^7^, suggesting that lava volume may herald future paroxysms^7^ or that slow decompression rates may control the transition from effusive to violent (paroxysmal) explosions^8^. The prolonged effusive phase in May–October 2014, however, did not culminate in a violent explosion^8,9^.

The two paroxysms of 3 July and 28 August 2019 occurred without appreciable lava effusion, but Strombolian activity intensified abruptly on 9 June 2019^10^, prior to the major explosion on 25 June. Frequent and unusual sustained lava fountaining persisted until 2 July^11^. The first paroxysm on 3 July was preceded by a small lava flow and edifice inflation ~45 and 10 minutes before the event^11–13^, respectively. The explosion formed an 8.4 km high eruption column^11^. Two pyroclastic currents descended the NW flank of the volcano (Sciara del Fuoco) and caused a ~1 m high tsunami^10,11,14^. After the 3 July paroxysm, explosive activity increased significantly for several weeks^10^ and discontinuous discharge of lava overtopped the southern crater area^10,11,14,15^. A second paroxysm on 28 August produced a 6.4 km high eruption column, a pyroclastic current, a smaller tsunami^10,11^, inflation of the edifice some 7 minutes before the event^16^ and a lava flow lasting a few hours. On 29 August, two major explosions occurred followed by lava effusion that lasted ~24 hours^14^. Volcanic activity returned to a normal level by 20 September 2019^10^.

Previous work on Stromboli’s 2019 paroxysms combining petrological and geophysical data^17^, implicated shallow conduit processes as triggers, while changing CO_2_ flux^18^ was interpreted to reflect an increasing amount of volatiles exsolved in the plumbing system since 2006, with an escalation in CO_2_ and SO_2_ degassing in the weeks to months before the paroxysms^19^. The focus of these earlier studies on the shallow plumbing system and concentration on either eruption precursors^10,12^, or possible triggering mechanisms^17–19^ have left key aspects of the role of the deeper plumbing system largely unexplored.

Here, we compare textural information and the compositional variability of clinopyroxene, glassy groundmass and bulk rocks from the major explosion on 25 June and the paroxysms on 3 July and 28 August with the products of normal Strombolian activity of 11 May 2019 and similar data of previous activity, particularly for 2003–2017^3^. By decoding complex zonation in clinopyroxene, and integrating thermobarometry and diffusion modelling results, we reconstruct the temporal paths of clinopyroxene populations to illuminate the role of magma recharge and mush remobilisation in modulating eruption dynamics. Our approach allows the interpretation of magmatic processes and their surficial expression, as recorded by monitoring data, showing that magma-mush dynamics exercise a key control on eruption style and magnitude at persistently active basaltic volcanoes.

## Results and discussion

### Petrography and geochemistry of the 2019 major explosion and paroxysms

The persistent mild Strombolian activity, effusive eruptions and major explosions, all involve a degassed, highly porphyritic (*hp*) magma from a shallow reservoir, seismically identified at 1–3 km depth^20^. Deep-seated (10–12 km depth^21^) more mafic and, volatile-rich low-porphyritic (*lp*) magma is erupted, alongside *hp-*magma, during paroxysms^21,22^, and in smaller quantities during some of the major explosions^23,24^.

**Fig. 2:**
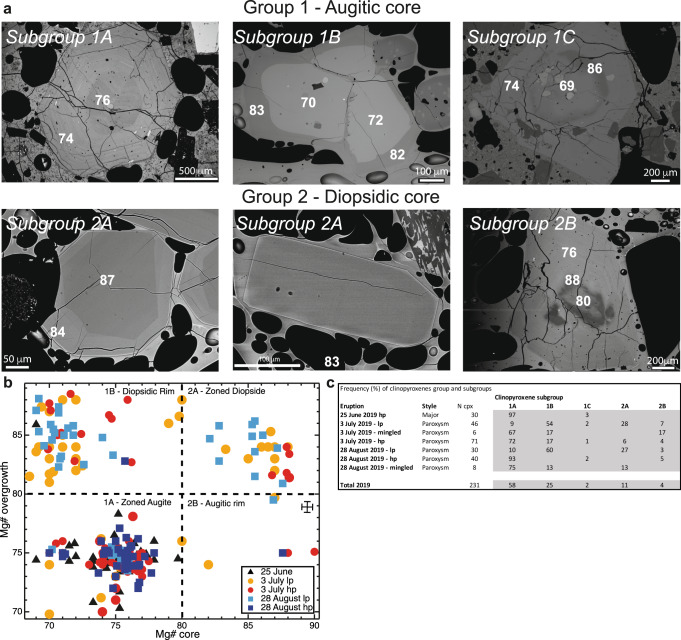
Textural classification, chemical composition, and frequency of 2019 clinopyroxenes. Clinopyroxenes from the major explosion of 25 June and the two paroxysms of 3 July and 28 August are euhedral with well-defined edges and tabular forms, with both phenocrysts (*Lmax* > 0.3 mm, on the basis of the longest size dimension) and microphenocrysts (*Lmax* = 0.3–0.1 mm; cf. Mollo et al.^67^) present (**a**) BSE (back-scattered electron) images for the two groups and respective subgroups of clinopyroxenes populations identified in the 25 June major explosions and the 3 July and 28 August paroxysms. The white numbers indicate the Mg# (Mg/(Mg+Fe^2+^_tot_) at.) for the different portions of clinopyroxene and correspond to augitic (Mg#_69–79_) and diopsidic (Mg#_80–90_) compositions. Subgroup 1A (zoned augite): Px3 sample P30-09-2-hp, 3 July paroxysm; subgroup 1B (diopsidic overgrowth): Px4-5 sample P24-3-lp, 3 July paroxysm; subgroup 1C (diopsidic band): Px35 sample 25062019, 25 June major explosion; subgroup 2A (zoned diopside): Px2 sample P01-05-lp, 3 July paroxysm and Px 7 (homogenous diopside), sample P44-1-lp, 28 August paroxysm; subgroup 2B (augitic overgrowth): Px1 sample P21-1-lp, 3 July paroxysm; **b** Mg#-overgrowth vs Mg#-core diagram for the 2019 clinopyroxenes. The chemical composition of each clinopyroxene core is plotted against the corresponding composition of the overgrowth for 25 June (black), 3 July *hp* (red) and *lp* (orange), 28 August *hp* (blue) and *lp* (cyan). The two main groups with the relative subgroups are indicated. Only the subgroup 1C is not plotted due to the low abundance of this crystal population. Mg# error bars are reported in the lower right quadrant. **c** Abundan**c**e (%) of clinopyroxene in each subgroup according to eruption (major, paroxysm) and magma (*hp*, *lp, mingled*) type. The total for all 2019 clinopyroxene is also given. *N*: number of clinopyroxenes for each subgroup.

Two products were erupted during both the 3 July and 28 August paroxysms: (1) typical *hp* black scoriae with high-porphyritic seriate texture, low vesicularity and microlite-rich glassy groundmass. This lithotype also characterises normal Strombolian activity and was the only one erupted during the major explosion on 25 June (Fig. 1b); (2) light *lp* pumice characterised by a weakly porphyritic seriate texture, high vesicularity and a glassy groundmass (Fig. 1c). Both endmembers have paragenesis of plagioclase, clinopyroxene and minor olivine (Fig. 1b, c), typical of products of the Present-day activity of Stromboli^3,22,25,26^ (i.e., <1.2 ka^27^). The two endmembers were syn-eruptively mingled, resulting in some homogenisation and crystal exchange between the two magmas (e.g., plagioclase in *lp* ejecta was largely derived by exchange from *hp-*magma, see Fig. 1c).

Bulk compositions of summer 2019 tephra are shoshonitic basalts typical of the Present-day activity and chemically similar to the *lp-*magma groundmass glass (Supplementary Fig. 1 and Supplementary Tables 1, 2). However, the glassy groundmass is compositionally bimodal (Supplementary Fig. 1).

### Clinopyroxene textures and compositions

Clinopyroxene (Supplementary Tab. 3) displays a broad compositional range from diopside (Di_69–80_Hd_9–16_En_8–15_Fs_1–3,_ Mg#_80–90_) to augite (Di_50-67_Hd_18-25_En_10-20_Fs_2-9,_ Mg#_69-79_) (see Methods) (Fig. 2b), as previously documented for the Present-day activity and interpreted as caused by the continuous *lp-hp* magma mixing and antecryst recycling from crystal mush^3,28–30^. Augitic compositions are in equilibrium with the more evolved resident *hp*-magma. Diopsidic compositions are in equilibrium with the more mafic *lp*-magma and their presence record inheritance from a mush region or recharging events of the more primitive *lp*-magma. Clinopyroxene can be texturally and chemically categorised into two main groups and several subgroups (Fig. 2).

Group 1 consists of clinopyroxene phenocrysts and microphenocrysts with an augitic core. Crystals are nearly homogeneous or slightly zoned (i.e., zoned augite subgroup 1A) or, alternatively, crystals have augitic cores with diopsidic rims (i.e., diopsidic overgrowth; subgroup 1B) reaching hundreds of microns (Fig. 2). Rare crystals from Group 1 show diopsidic recharge bands, appearing as sharp to diffused boundaries around euhedral to resorbed augitic cores and euhedral augitic rims (subgroup 1C). Group 1, particularly 1A is modally dominant (85% of crystals; Fig. 2c).

Group 2 clinopyroxene phenocrysts and microphenocrysts (15% of crystals; Fig. 2c) have diopsidic cores (Fig. 2a, b). These crystals are homogeneous or slightly zoned (subgroup 2A zoned diopside), or they have diopsidic cores with dissolution-reaction features (i.e., cores are resorbed and likely antecrystic) and augitic overgrowths (subgroup 2B; Fig. 2). It is worth noting that not all subgroups are represented in each tephra. The main differences in clinopyroxene cargoes are summarised in Fig. 2c.

**Fig. 3:**
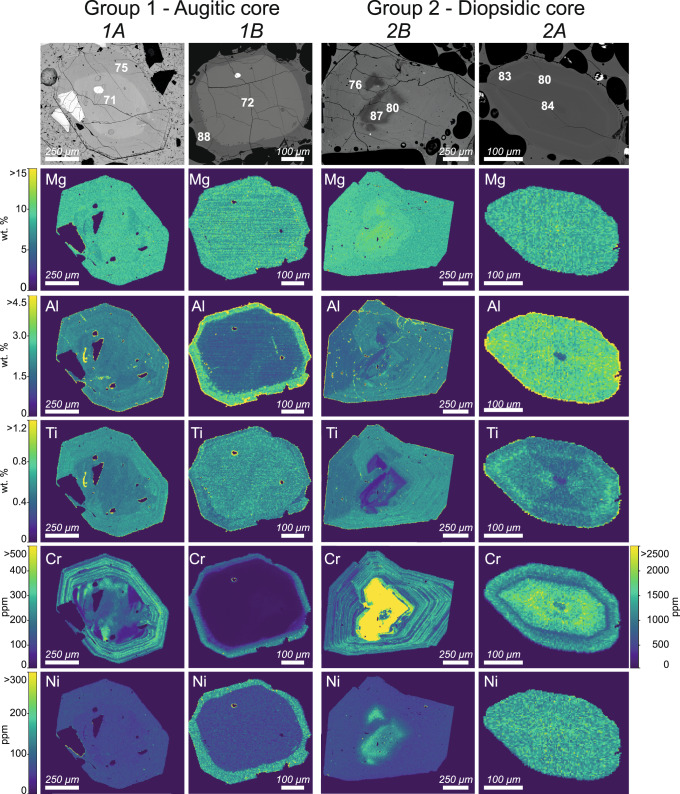
Major and trace elements chemical mapping for 2019 clinopyroxenes. BSE images (top) and quantitative chemical mapping of Mg, Al, Ti (wt%), Cr and Ni (ppm) for the main four subgroups of clinopyroxenes. Subgroup 1A: Px 11 sample P27-1-hp, 3 July paroxysm; subgroup 1B: Px6 sample P30-07-lp, 3 July paroxysm; subgroup 2B: Px1 sample P-21-1-lp, 3 July paroxysm; subgroup 2A: Px13 sample P30-07-lp, 3 July paroxysm. The scale bar for Cr is on the left for subgroup 1A, 1B and 2B and on the right for subgroup 2A.

Chemical mapping of augite crystals of subgroup 1A and augitic overgrowths of subgroup 2B crystals (Fig. 3, Supplementary Figs. 2–3) shows a finely banded, oscillatory zoning pattern in Cr, Al, and Ti. In subgroup 1A crystals, this oscillatory zoning is superimposed on the main low-amplitude augitic core-rim zoning where ΔMg# (=Mg#_overgrowth_ – Mg#_core_) ranges between + 5 and −5. The increase in Mg# in the zoned augite crystals of subgroup 1A may or may not correspond to a modest increase in Cr content (Fig. 3, Supplementary Figs. 2–3). By contrast, augitic cores of subgroup 1B crystals are homogenous (Fig. 3, Supplementary Fig. 2). Group 2 and diopsidic rims of subgroup 1B crystals typically have elevated concentrations of Mg, Al, Ni and Cr (>2500 ppm) (Fig. 3, Supplementary Figs. 2–3).

### 2003-2019 clinopyroxene geochemical evolution and *P*-*T*-H_2_O crystallisation conditions

**Fig. 4:**
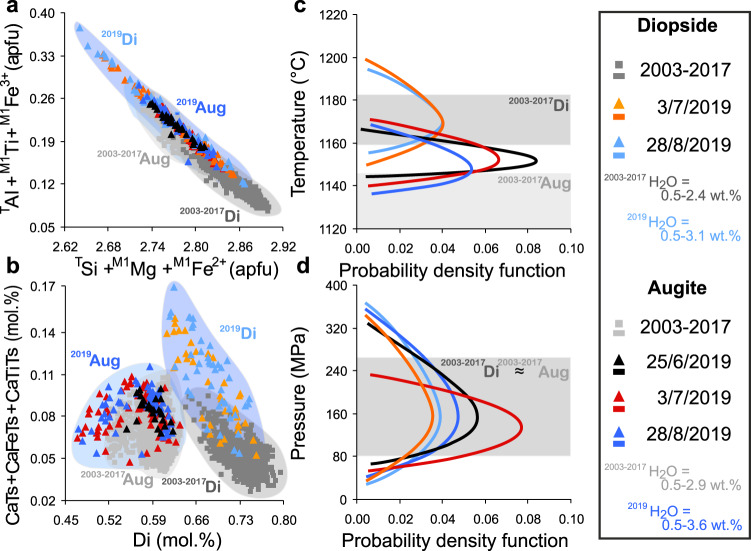
Chemical composition of 2019 clinopyroxenes and *P-T* crystallisation conditions. **a** Al+Ti+Fe^3+^ (apfu) vs. Si+Mg+Fe^2+^ (apfu) and (**b**) CaTs+CaFeTs+CaTiTs (mol.%) vs. Diopside (Di, mol%) diagrams showing the chemical composition and range of variability of augitic and diopsidic portions of the 2019 clinopyroxenes (black triangle: 25 June major explosion; orange and red triangle: 3 July paroxysm; and cyan and blue triangle: 28 August paroxysm) compared with the augitic and diopsidic composition of 2003–2017 clinopyroxenes (grey field and square) from Di Stefano et al.^3^. At the scale of the figure, error bars are smaller than the symbols. CaTs+CaFeTs+CaTiTs (mol.%) show the Tschermak-style cation substitution [^T^Si, ^M1^Mg, ^M1^Fe^2+^] → [^T^Al, ^M1^Ti, ^M1^Fe^3+^]. Probability density function of temperature (**c**) and pressure (**d**) for 2019 augite (black line: 25 June, based on 28 datapoints; red line: 3 July, 82 datapoints; and blue line: 28 august, 56 datapoints) and diopside (orange line: 3 July, 37 datapoints; cyan: 28 August, 44 datapoints) compositions compared with the *P-T* range of 2003-2007 eruptive period (grey band) from Di Stefano et al.^3^. Clinopyroxene-based pressure estimates do not vary significantly among the different diopsidic-augitic crystal portions due to the lack of substantial changes in the *P*-sensitive jadeite-melt exchange reaction at relative low-pressure conditions^68,69^. Estimated H_2_O contents for 2019 (this study) and 2003–2017^3^ are also given. Augite equilibrates at a slightly higher melt-H_2_O content relative to diopside due to the higher portion of solid fraction during the late stage of clinopyroxene growth, before abundant *hp*-magma degassing and plagioclase crystallisation during magma ascent towards the surface^3^. Indeed, the amount of H_2_O dissolved in Stromboli magma reaches ~2.7–3.5 wt.% prior to outgassing^21^, in agreement with the maximum melt-H_2_O content recorded by 2019 clinopyroxene. There is also a positive correlation between the ratio of iron to magnesium in clinopyroxene and the amount of H_2_O dissolved in the melt^67^. In particular, the ^M1^Fe^2+^/^M1^Mg ratio of 2019 clinopyroxene is systematically higher than that of 2003–2017 crystals, which is consistent with their higher melt-H_2_O concentrations.

Composition of diopsidic and augitic clinopyroxenes from the major explosion of 25 June and the paroxysms of 3 July and 28 August 2019 notably differ from those erupted during the 2003–2017 activity^3^ (Fig. 4a, b). In particular, crystal populations from 2019 eruptions are more enriched in ^T^Al + ^M1^Ti + ^M1^Fe^3+^ and depleted in ^T^Si + ^M1^Mg + ^M1^Fe^2+^ compared with those from 2003–2017 eruptions (Fig. 4a) with Tschermak components preferentially incorporated in 2019 crystals at the expense of diopside (Di; Fig. 4b). Nevertheless, both 2019 and 2003–2017 clinopyroxenes align along a single trend of Tschermak component (CaTs + CaTiTs + CaFeTs) substitution (Fig. 4a), suggesting a common geochemical evolution, which is consistent with the relative magma uniformity across 2003-2019 and the entire Present-day activity (Supplementary Fig. 1).

Enhanced Tschermak substitution may reflect higher temperature crystallisation^31,32^ or more hydrous conditions^33^. Probability density functions^3^ for *T* show that 2019 diopsidic and augitic compositions peak at ~1175 ± 16 and ~1155 ± 16 °C, respectively (Fig. 4c, see Methods for *T-P-H*_*2*_*O* calculation schemes). Comparing 2019 diopsidic clinopyroxene with those erupted from the Present-day activity^3,34^ indicates preferential equilibration of diopside within the deep-seated *lp*-magma at *T* > 1170 °C, with near-liquidus mineral phase appearance at ~1200 °C. Conversely, 2019 augitic clinopyroxene saturates the *hp*-magma at temperatures higher than those estimated for the Present-day activity (i.e., *T* < 1145 °C^3,34^), indicating a hotter reservoir in 2019.

Probability density functions for *P* show that both 2019 diopsidic and augitic compositions peak at ~150 ± 100 MPa (Fig. 4d), consistent with a near-invariant pressure range obtained for the 2003–2017 crystals (~180 ± 100 MPa^3^). The stability of the magmatic reservoir, with magma ponding at the same level (also observed in olivine^5^), confirms that Stromboli’s plumbing system is characterised by a shallow reservoir located at ~50–150 MPa (i.e., ~1.5–6 km depth assuming a crustal density of 2700 kg/m^3^;^5,21,25^). In this reservoir, the resident *hp*-magma crystallises augitic clinopyroxene, olivine and abundant plagioclase^22,29,30,35,36^. An additional deeper reservoir, inferred from melt inclusion and water solubility data^21^, is located at ~190–260 MPa (~7–10 km depth^21^), where the feeding *lp*-magma undergoes limited crystallisation of diopsidic clinopyroxene and olivine^3,21,30^. Thus, the steady-state dynamics established since at least ~1.7 ka ago, and sustained by continuous refilling of a shallow *hp-*reservoir by volatile-rich *lp*-magma^30^, continue to control the system’s evolution. Syn-eruptive mingling of compositionally similar *lp-hp* basaltic magmas leads to the observed range in crystallinity^3,22,28–30,36^.

Melt-H_2_O concentrations in equilibrium with 2019 clinopyroxene range from 0.5–3.1 wt.% (diopside) and 0.5–3.6 wt.% (augite), slightly higher than those of the 2003–2017 period (0.5–2.4 and 0.5–2.9 wt.% for diopside and augite, respectively) and consistent with the Fe:Mg ratio in clinopyroxene (Fig. 4).

### Mafic recharge as eruption trigger of the 2019 paroxysms

Diopsidic portions of subgroups 1B, 1C, 2A and 2B exhibit high Cr concentrations, elevated concentrations of Ni, Mg and Al and lower concentrations of Ti (Fig. 3, Supplementary Figs. 2–3). The elevated Cr reflects crystallisation from mafic, Cr-rich *lp*-magma. Diopsidic cores of Group 2 crystals likely equilibrated at depth, whereas diopsidic rims (and diopsidic bands) on augitic clinopyroxene formed because of mafic recharge due to convective mass transfer^37^ from depth of *lp-*magma into *hp-*magma. Thus, subgroup 2A crystals reflect crystallisation entirely within the deep *lp* magmatic domain that is compositionally distinct from the shallow *hp* reservoir, where augitic clinopyroxene crystallises^3,22,29,30,36^. By contrast, resorbed diopsidic and augitic cores of subgroup 2B and some 2C crystals, respectively, are interpreted as disrupted portions of a vertically-extended mush column situated at the base of the *hp*- reservoir^28,36,38^ or encompassing both the shallow and deeper reservoirs^3,39^ and, thus, as antecrysts in line with the previous studies^3,28,30,36,38,39^. The predominance of subgroup 1B crystals in *lp* products and their abundance in *hp* and mingled products erupted during the paroxysms suggest interaction between *hp* and *lp* magmas prior to eruption. This further suggest that new injected mafic *lp-*magma(s) induced mixing and crystallisation (i.e., the diopsidic overgrowth of subgroup 1B) and triggered the 2019 paroxysms. At the same time, the absence of subgroup 1B and group 2 (particularly subgroup 2B) crystals, alongside the scarcity of subgroup 1C in the major explosion of 25 June and the presence of subgroups 1B and group 2 exclusively in paroxysmal products of 3 July and 28 August (Fig. 2c) clearly indicates mush remobilisation only occurred during these powerful events.

**Fig. 5:**
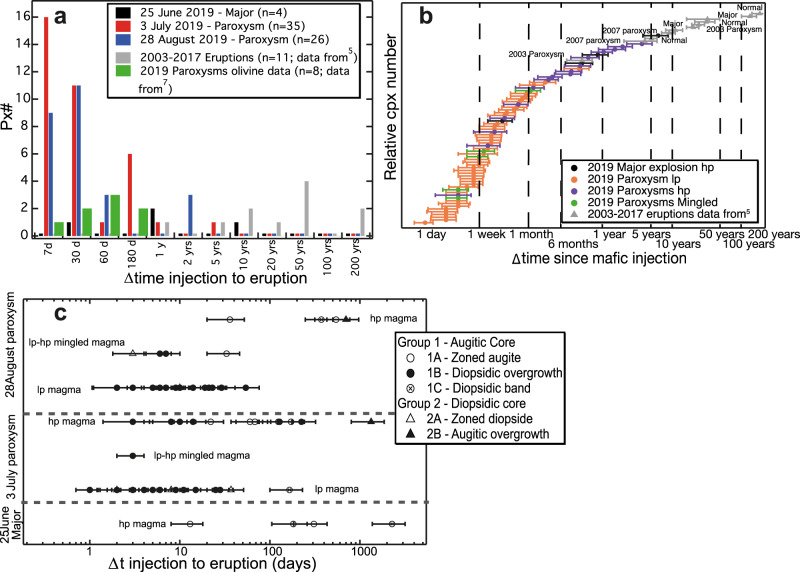
Fe-Mg timescales for the 2019 clinopyroxenes. **a** Histogram showing the number of clinopyroxene *vs* timescales ($\triangle$time injection – eruption, in days and years) from mafic injection to eruption for 25 June (black) major explosion, 3 July (red) and 28 August (blue) paroxysms. Timescales for the 2003–2017 eruptive period from Di Stefano et al.^3^ are also shown (grey), alongside the timescales calculated from olivine crystals by Métrich et al.^5^ for the two 2019 paroxysms (green). *n* = number of timescales estimate. **b** Timescales are presented in order of increasing $\triangle$time and are plotted according to the different type of erupted magmas (filled circle) – black: 25 June major explosion *hp*-magma; orange: 3 July and 28 August paroxysms *lp*-magma; purple: 3 July and 28 August paroxysms *hp*-magma; green: 3 July and 28 August paroxysms *mingled*-magma. Timescales for the 2003–2017 eruptive period from Di Stefano et al.^3^ are also shown (grey triangle). **c** Timescales (in days) for the 2019 major explosion and paroxysms are presented according to different pyroxenes subgroups. The majority of timescales obtained from the 3 July and 28 August (*lp* and mingled composition) paroxysms were calculated from subgroup 1B clinopyroxenes, which therefore represent the time between growth of the diopsidic rim and eruption caused by injection of mafic magma into the shallow reservoir prior to eruption. These short timescales correspond to the eruption-triggering event as discussed in the text.

Fe-Mg diffusive timescales of recharge and magma mixing events were determined on 65 clinopyroxenes from the major explosion of 25 June and the two paroxysms of 3 July and 28 August 2019 through the non-isothermal approach (NIDIS)^40^ (see Methods). Timescales for all three eruptive events range from a few days to 6 years (Table 1, Fig. 5 and Supplementary Figs. 6–64), with 74% of crystals recording timescales $\le{1}$ month pre-eruption (Fig. 5a, b). Clinopyroxenes from *lp* tephra erupted during the two paroxysms return short timescales from a few days to a couple of months (Table 1), with mingled and *hp* products from the two paroxysms indicating timescales in the order of days to ~4 years. The major explosion on 25 June shows longer timescales between 6 ± 2 months and 6 ± 2 years, with only one crystal recording a timescale of a few days (Fig. 5b, Table 1). In stark contrast are the longer timescales (~1–182 years) reported for the 2003–2017 period^3^, although these timescales were determined for clinopyroxenes characterised by diopsidic resorbed cores and diopsidic bands (similar to our subgroup 2B and 1C). In fact, according to Di Stefano et al.^3^ the 2003–2017 period is characterised by a lack of subgroup 1B crystals and a predominance of diopsidic resorbed cores, but clinopyroxenes with diopsidic rims occur in both 2003 *lp*-pumices and 2007 *hp*-lava flows^39,41,42^, although no timescales information is available for these crystals.

**Table 1:** Fe-Mg timescales calculated for clinopyroxenes from 2019 eruptions

Eruption	Sample	Product Type	Cpx n.	Cpx subgroup	T °C ± 16	Δt days	Δt months	Δt years	Absolute eruption date	Timescale type
25-Jun-19	25062019	hp	PX8	1 A	1155	182 ± 77	6 ± 3	0.5 ± 0.2	25/12/2018	Convective mixing
25-Jun-19	25062019	hp	PX16	1 A	1155	308 ± 124	10 ± 4	0.8 ± 0.3	21/08/2018	Convective mixing
25-Jun-19	25062019	hp	PX18	1 A	1155	13 ± 5	0.4 ± 0.2	0.03 ± 0.01	12/06/2019	Convective mixing
25-Jun-19	25062019	hp	PX35	1 C	1155	2274 ± 901	75 ± 30	6 ± 2	03/04/2013	Antecryst remobilisation
03-Jul-19	P30-07	lp	Px6	1B	1175	4 ± 2	0.13 ± 0.06	0.01 ± 0.01	29/06/2019	Mafic trigger
03-Jul-19	P30-07	lp	Px9	1B	1175	3 ± 1	0.08 ± 0.04	0.001 ± 0.003	30/06/2019	Mafic trigger
03-Jul-19	P30-07	lp	Px11	1B	1175	3 ± 1	0.11 ± 0.05	0.01 ± 0.004	30/06/2019	Mafic trigger
03-Jul-19	P10-5	lp	Px4	1B	1175	1 ± 0.3	0.02 ± 0.01	0.002 ± 0.001	02/07/2019	Mafic trigger
03-Jul-19	P01-05	lp	PX2	2 A	1175	2 ± 0.8	0.06 ± 0.03	0.005 ± 0.002	01/07/2019	Convective mixing
03-Jul-19	P01-05	lp	PX3	1B	1175	5 ± 2	0.15 ± 0.06	0.01 ± 0.01	28/06/2019	Mafic trigger
03-Jul-19	P01-05	lp	PX4	1B	1175	9 ± 3	0.29 ± 0.11	0.02 ± 0.01	24/06/2019	Mafic trigger
03-Jul-19	P01-05	lp	PX5	1B	1175	28 ± 11	0.9 ± 0.4	0.08 ± 0.003	05/06/2019	Mafic trigger
03-Jul-19	P01-05	lp	PX6	1B	1175	5 ± 2	0.15 ± 0.06	0.01 ± 0.01	28/06/2019	Mafic trigger
03-Jul-19	P01-05	lp	PX7	1B	1175	25 ± 10	0.8 ± 0.3	0.07 ± 0.03	08/06/2019	Mafic trigger
03-Jul-19	P01-05	lp	PX9	1 C		165 ± 65	5 ± 2	0.4 ± 0.2	19/01/2019	Antecryst remobilisation
03-Jul-19	P01-05	lp	PX11	1B	1175	5 ± 2	0.16 ± 0.06	0.01 ± 0.01	28/06/2019	Mafic trigger
03-Jul-19	P21-1	lp	Px3	1B	1175	15 ± 5	0.5 ± 0.2	0.04 ± 0.02	18/06/2019	Mafic trigger
03-Jul-19	P21-1	lp	Px5	2 A	1175	37 ± 14	1.2 ± 0.4	0.10 ± 0.04	27/05/2019	Convective mixing
03-Jul-19	P21-1	lp	Px6	2 A	1175	8 ± 3	0.28 ± 0.11	0.02 ± 0.001	25/06/2019	Convective mixing
03-Jul-19	P21-1	lp	Px7	1B	1175	5 ± 2	0.16 ± 0.06	0.013 ± 0.005	28/06/2019	Mafic trigger
03-Jul-19	P21-1	lp	Px8	1B	1175	11 ± 4	0.35 ± 0.13	0.03 ± 0.01	22/06/2019	Mafic trigger
03-Jul-19	P24-3	lp	Px3	1B	1175	9 ± 3	0.29 ± 0.11	0.02 ± 0.01	24/06/2019	Mafic trigger
03-Jul-19	P24-3	lp	Px4	1B	1175	6 ± 2	0.18 ± 0.07	0.02 ± 0.01	27/06/2019	Mafic trigger
03-Jul-19	P24-3	lp	Px5	1B	1175	3 ± 1	0.09 ± 0.04	0.01 ± 0.003	30/06/2019	Mafic trigger
03-Jul-19	P24-3	lp	Px6	1B	1175	2 ± 1	0.07 ± 0.003	0.01 ± 0.002	01/07/2019	Mafic trigger
03-Jul-19	P24-3	lp	Px7	1B	1175	6 ± 2	0.21 v0.08	0.02 ± 0.01	27/06/2019	Mafic trigger
03-Jul-19	P24-3	lp	Px8	1B	1175	2 ± 1	0.06 ± 0.03	0.005 ± 0.002	01/07/2019	Mafic trigger
03-Jul-19	P24-3	mingled	Px 07	1B	1175	3 ± 1	0.09 ± 0.03	0.007 ± 0.003	30/06/2019	Mafic trigger
03-Jul-19	P30-07	hp	Px28	1B	1175	3 ± 2	0.10 ± 0.05	0.01 ± 0.004	30/06/2019	Mafic trigger
03-Jul-19	P30-07	hp	Px31	2B	1155	1330 ± 528	44 ± 17	4 ± 1	11/11/2015	Antecryst remobilisation
03-Jul-19	P30-07	hp	Px5	1B	1175	10 ± 5	0.33 ± 0.15	0.03 ± 0.01	23/06/2019	Mafic trigger
03-Jul-19	P30-07	hp	Px10	1B	1175	8 ± 4	0.27 ± 0.12	0.02 ± 0.02	25/06/2019	Mafic trigger
03-Jul-19	P30-07	hp	Px19	1B	1175	128 ± 52	4 ± 2	0.35 ± 0.01	25/02/2019	Mafic trigger
03-Jul-19	P30-07	hp	Px20	1B	1175	224 ± 98	7 ± 3	0.6 ± 0.3	21/11/2018	Mafic trigger
03-Jul-19	P30-09	hp	Px6a	1 A	1155	68 ± 26	2 ± 1	0.19 ± 0.01	26/04/2019	Convective mixing
03-Jul-19	P30-09	hp	Px6b	1 A	1155	60 ± 23	2 ± 1	0.2 ± 0.1	04/05/2019	Convective mixing
03-Jul-19	P30-09	hp	Px9b	1 A	1155	124 ± 51	4 ± 2	0.3 ± 0.1	01/03/2019	Convective mixing
03-Jul-19	P30-09	hp	Px13a	1 A	1155	172 ± 70	6 ± 2	0.5 ± 0.1	12/01/2019	Convective mixing
03-Jul-19	P30-09	hp	PX13 e	1 A	1155	22 ± 9	0.7 ± 0.5	0.06 ± 0.03	11/06/2019	Convective mixing
03-Jul-19	P30-09	hp	Px22	1B	1175	14 ± 5	0.5 ± 0.2	0.04 ± 0.01	19/06/2019	Mafic trigger
28-Aug-19	P44-1	lp	Px1	1B	1175	5 ± 2	0.15 ± 0.06	0.01 ± 0.01	23/08/2019	Mafic trigger
28-Aug-19	P44-1	lp	Px2	1B	1175	5 ± 2	0.18 ± 0.08	0.01 ± 0.01	23/08/2019	Mafic trigger
28-Aug-19	P44-1	lp	Px3	1B	1175	14 ± 6	0.46 ± 0.18	0.04 ± 0.02	14/08/2019	Mafic trigger
28-Aug-19	P44-1	lp	Px4	1B	1175	3 ± 1	0.09 ± 0.04	0.010 ± 0.003	25/08/2019	Mafic trigger
28-Aug-19	P44-1	lp	Px6	2 A	1175	10 ± 4	0.33 ± 0.13	0.03 ± 0.01	18/08/2019	Convective mixing
28-Aug-19	P44-1	lp	Px11	1B	1175	10 ± 4	0.33 ± 0.13	0.03 ± 0.01	18/08/2019	Mafic trigger
28-Aug-19	P47-1	lp	Px2	1B	1155 6	7 ± 4	0.23 ± 0.12	0.02 ± 0.01	21/08/2019	Mafic trigger
28-Aug-19	P47-1	lp	Px6	1B	1175	9 ± 4	0.3 ± 0.11	0.02 ± 0.01	19/08/2019	Mafic trigger
28-Aug-19	P47-1	lp	Px11	1B	1175	54 ± 22	1.8 ± 0.7	0.2 ± 0.1	05/07/2019	Mafic trigger
28-Aug-19	P47-1	lp	Px12	1B	1175	21 ± 8	0.7 ± 0.3	0.06 ± 0.02	07/08/2019	Mafic trigger
28-Aug-19	P47-1	lp	Px13	1B	1175	23 ± 10	0.76 ± 0.33	0.06 ± 0.03	05/08/2019	Mafic trigger
28-Aug-19	P47-1	lp	Px14	1B	1175	6 ± 2	0.19 ± 0.07	0.02 ± 0.01	22/08/2019	Mafic trigger
28-Aug-19	P47-1	lp	Px15	1B	1175	29 ± 12	0.9 ± 0.38	0.08 ± 0.03	30/07/2019	Mafic trigger
28-Aug-19	P47-1	lp	Px16	1B	1175	9 ± 4	0.29 ± 0.12	0.02 ± 0.01	19/08/2019	Mafic trigger
28-Aug-19	P47-1	lp	Px17	1B	1175	2 ± 1	0.08 ± 0.03	0.007 ± 0.003	26/08/2019	Mafic trigger
28-Aug-19	P47-1	lp	Px18	1B	1175	2 ± 1	0.07 ± 0.03	0.006 ± 0.002	26/08/2019	Mafic trigger
28-Aug-19	P47-1	lp	Px19	1B	1175	19 ± 8	0.6 ± 0.3	0.05 ± 0.02	09/08/2019	Mafic trigger
28-Aug-19	P47-1	lp	Px20	1B	1175	5 ± 2	0.16 ± 0.07	0.01 ± 0.01	23/08/2019	Mafic trigger
28-Aug-19	P47-1	hp	Px11	1 A	1155	543 ± 228	18 ± 8	1.5 ± 0.6	03/03/2018	Convective mixing
28-Aug-19	P47-1	hp	Px12	2B	1155	699 ± 270	23 ± 9	1.9 ± 0.7	28/09/2017	Antecryst remobilisation
28-Aug-19	P47-1	hp	Px6	1 C		373 ± 126	12 ± 4	1 ± 0.4	20/08/2018	Mafic recharge + storage
28-Aug-19	P44-1	hp	Px3	1 A	1155	36 ± 16	1.2 ± 0.5	0.01 ± 0.04	23/07/2019	Convective mixing
28-Aug-19	P44-1	mingled	Px3	1 A	1155	33 ± 13	1.1 ± 0.4	0.09 ± 0.04	26/07/2019	Convective mixing
28-Aug-19	P44-1	mingled	Px4	1B	1175	7 ± 3	0.2 ± 0.1	0.02 ± 0.01	21/08/2019	Mafic trigger
28-Aug-19	P44-1	mingled	Px6	2 A	1175	3 ± 1	0.1 ± 0.04	0.008 ± 0.003	25/08/2019	Convective mixing
28-Aug-19	P44-1	mingled	Px8	1B	1175	6 ± 2	0.19 ± 0.07	0.02 ± 0.01	22/08/2019	Mafic trigger

Temperatures refer to those used in the diffusion modelling according to thermobarometric results and considering 2 sigma error of ±16 °C, Fe-Mg diffusion coefficient were calculated at 1155 and 1175 °C for augitic and diopsidic composition, respectively. For two crystals of subgroup 1C, temperature is not reported because in this case T of 1175 ± 16 °C and 1155 ± 16 °C were used to calculate the total timescale across the mafic recharge band (see Supplementary Figs. 57–58 and Methods for further explanation). This timescale according to the NIDIS model^40^ refers to: (1) the timescale from the moment the diopsidic band formed upon arrival of the recharge mafic lp-magma, until the eruption after the augitic overgrowth formed and the crystal was stored in the shallow *hp*-reservoir (mafic recharge + storage); (2) the timescale from the moment the resorbed augitic core was remobilised from the crystal mush by the ascent of the *lp*-magma, until the eruption after the augitic overgrowth formed and the crystal was stored in the shallow *hp*-reservoir (antecryst remobilisation). Timescales type refers to the different types of timescales as discussed in the text (i.e., mafic trigger; convective mixing; mafic recharge + storage; antecryst remobilisation). 25-Jun-19: major explosion; 3-Jul-19 and 18-Aug-19: paroxysm.

The very short timescales from the diopsidic overgrowth of subgroup 1B clinopyroxenes (a few days- to a couple of months, with two outliers of 128 and 224 days, Fig. 5c) indicate the magmatic system responded rapidly to injection of mafic magma(s) prior to the two paroxysms. In contrast to other proposed models^5,17,19^, interaction between the *lp-* and *hp-*magma in the Stromboli’s shallow reservoir extends beyond syn-eruptive mingling with *lp* mafic recharge arriving in the shallow reservoir months before the 2019 paroxysmal events inducing limited and incomplete magma mixing associated with diopsidic overgrowth induced crystallisation. Accordingly, 1B crystals confirm rapid overgrowth of diopsidic portions around augitic cores following injection of *lp*-magma into the shallow *hp*-reservoir (Fig. 3, Supplementary Figs. 2–3). The importance of *lp* mafic recharge actively interacting with the *hp-*magma in the months prior the 2019 paroxysms is emphasised by the prevalence of subgroup 1B crystals in *lp* products and their abundance in *hp* and mingled samples of the paroxysms (Fig. 2). Moreover, the abundance in the *lp* samples of zoned diopsidic 2A clinopyroxene, which formed in the deeper more mafic *lp* recharge magma and lack augitic overgrowths (Figs. 2–3, Supplementary Figs. 2–3 and 59–62), suggests incomplete mixing and limited residence time of the recharge magma(s) in the shallow reservoir. Short crystal residence times (5–110 days for 3 July and 10-115 days for 28 August, albeit without uncertainties) were also derived from olivine by Métrich et al.^5^ (Fig. 5a). Our recorded recharges from month(s) to days before the 3 July and 28 August 2019 events (Fig. 5b, c) substantiate the role of the deeper magmatic system in triggering both paroxysms, well beyond volatile flushing^13,17–19^ by feeding fresh *lp*-magma continuously into the shallow *hp*-reservoir from ~1–2 months prior to each paroxysmal event.

Regarding the major explosion of 25 June, the absence of subgroup 1B clinopyroxenes, and the longer timescales (days to 6 years) measured in subgroup 1A and 1C crystals (see below) demonstrates that *lp*-magma did not directly triggered this eruption.

### Convective mixing regime controlling magma dynamics

**Fig. 6:**
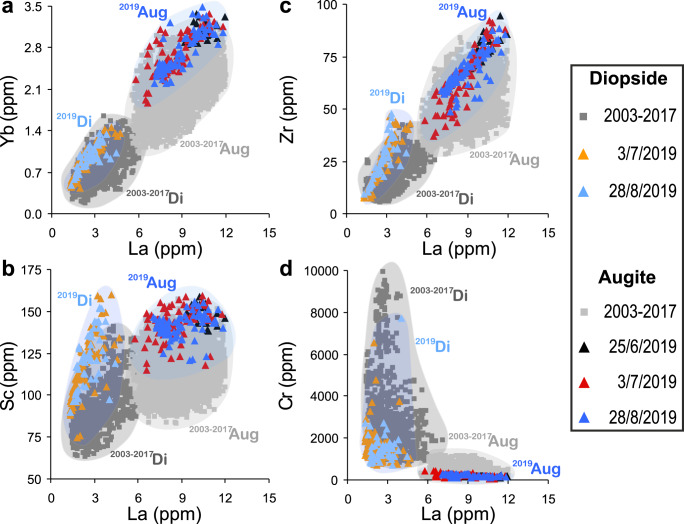
Trace elements of 2019 clinopyroxenes. **a** Yb; **b** Sc; **c** Zr, and **d** Cr vs La (ppm) diagrams showing the trace elements range of variability of augitic (Aug) and diopsidic (Di) portions of the 2019 clinopyroxenes (black triangle: 25 June major explosion; orange and red triangle: 3 July paroxysm; and cyan and blue triangle: 28 August paroxysm) compared with the augitic and diopsidic composition of 2003–2017 clinopyroxenes (grey field) from Di Stefano et al.^3^. At the scale of the figure, error bars are smaller than the symbols.

Both diopsidic and augitic clinopyroxenes from the major explosions and paroxysms in 2019 overlap compositionally with crystals from 2003–2017 (Fig. 6, Supplementary Tab. 4). However, notable differences exist in the uptake of elements in the lattice sites (Fig. 6, Supplementary Fig. 4). Dynamic stirring experiments^37^ indicate that fine low-amplitude concentric zoning may result from kinetic crystallisation under convective stirring. Therefore, Cr-rich concentric zones may not necessarily correlate with other geochemical indices of recharge^37^. Augitic portions and overgrowth of subgroup 1A, 1C, and 2B crystals, and some diopsidic portions of subgroup 2A crystals are characterised by fine oscillatory zoning, sometimes associated with Cr-rich concentric zones (Figs. 2 and 3, Supplementary Figs. 2–3) with limited correlation with other elements. This contrasts with the incorporation of Cr in clinopyroxene resulting from mafic recharge. This fine oscillatory zoning in the augitic portions of 1A, 1C and 2B (and some 2A) crystals may result from convective stirring, hinting that during the 2019 major and paroxysmal activity, the shallow *hp*-reservoir, and possibly also the deeper *lp*-reservoir, underwent continuous convection, promoting crystallisation and minimising kinetic effects on clinopyroxene zoning^37^.

Superimposed on the fine oscillatory zoning, zoned augites and diopsides (subgroups 1A and 2A) show slightly larger but still relatively low-amplitude compositional variations (Figs. 2 and 3). Several subgroup 1A crystals and some subgroups 2A (e.g., Fig. 2, Supplementary Figs. 2–3, 6–14, and 60–61) crystals have rounded cores, indicating dissolution and reaction with the crystallising magma (*hp* or *lp* respectively) but with some minor compositional variation resulting from local stronger convective regime whereby growth kinetics and diffusion boundary layers were not reduced by the homogenising effect of stirring^37^. Modification of these compositional boundaries by diffusion, where the slow diffusing elements Al and Ca show a sharp change that mimics the Mg profile (Supplementary Figs. 6–14 and 59–62) can therefore be used to obtain time constraints of the convective regime.

Fe-Mg diffusion timescales of the larger compositional zoning for zoned augite of subgroups 1A range from 543 ± 228 to a few days, for the major explosions of 25 June and the two paroxysms, whereas those for zoned diopside of subgroup 2A crystals are on average shorter, ranging from days to <2 months (Table 1; Fig. 5c, Supplementary Figs. 6–14 and 59–62). These timescales overlap with those of the mafic triggering events (see above), suggesting magnification of convection by mafic recharge event(s). The presence of zoned augites, alongside diopsidic and augitic overgrowth, indicates that mafic recharge destabilised the shallower parts of the system, thereby establishing a chaotic mixing regime similar to that postulated for Post-Pizzo period^30^ (1.7–1.5 ka^43^), favouring the formation of subgroup 1A (i.e., crystallisation of the oscillatory zoned augitic overgrowth). Similarly, the presence of zoned diopside subgroup 2A crystals with shorter timescales suggest that during June-August 2019, dynamic crystallisation also characterised the deeper *lp*-reservoir, leading to diopsidic overgrowth of 2 A crystals around pre-existing diopsidic cores, shortly before mafic recharges rapidly triggered the paroxysms.

### The lifecycle of basaltic crystal mush

**Fig. 7:**
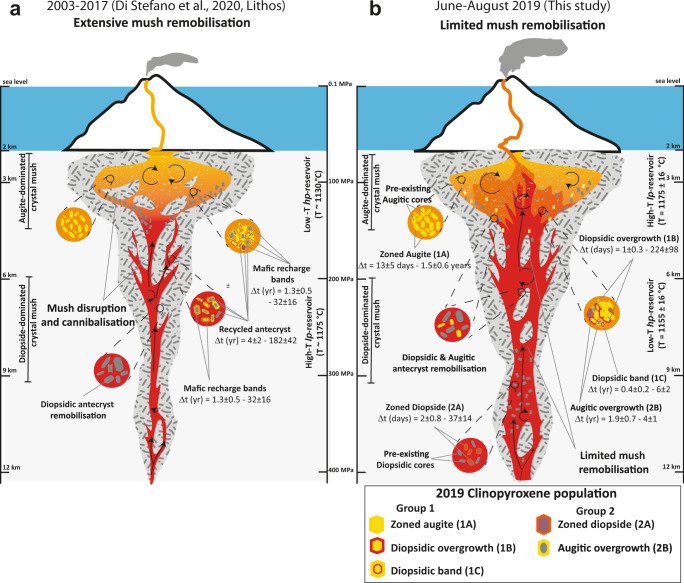
Schematic model of the plumbing system at Stromboli volcano. **a** 2003–2017 eruptive period adapted from Di Stefano et al.;^3^ **b** June–August 2019 (this study). During the 2003–2017 eruptive period extensive mush remobilisation and cannibalisation operated by the uprising deeper undegassed mafic recharge *lp*-magma (in red) characterises magma dynamics. The *lp*-magma is fed by the deeper reservoir as inferred by Métrich et al.^21^. Abundant recycled diopsidic antecrysts are remobilised from the crystal mush and transported by the *lp*-magmas permeating the mush, into the shallower *hp*-reservoir (yellow-orange). The continuous and efficient remobilisation of the mush between 2003 and 2017 led to a rejuvenated plumbing system configuration in 2019, characterised by the *lp*-magma extensively permeating the mush with limited mush remobilisation. A few recycled diopsidic and augitic antecrysts are remobilised and transported by the *lp*-magma into the shallower *hp*-reservoir where the augitic overgrowth forms around resorbed diopsidic core (subgroup 2B) or resorbed augitic core surrounded by diopsidic band (subgroups 2C). Zoned or homogenous diopside (subgroup 2A) and augite (subgroup 1A) formed within the *lp*- and *hp*-reservoirs, respectively. The continuous arrival of *lp-*magma determines a convective mixing (black rounded arrows) in both reservoirs. Such highly dynamic environments originate both the zoned crystals and the low-amplitude zonation of the overgrowth portion. The abundance of diopsidic overgrowth (subgroup 1B) and their short timescales indicate that the 2019 paroxysmal events were triggered by injection of new *lp*-magma from a few months to a few days before the explosion onset. The schematic model has been modified and adapted from Di Stefano et al.^3^.

Despite the steady-state and similar configuration of Stromboli’s plumbing system, the marked textural and compositional differences between the 2019 clinopyroxenes and those from the 2003–2017 period^3,38,39,42^ (Figs. 2 and 4), but also from previous activity (i.e., 1984-1996^28^), require significantly different magma dynamics (Fig. 7). A striking difference is the paucity of crystals with resorbed (antecrystic) diopsidic and augitic cores (subgroup 2B and some subgroup 1C crystals, Fig. 2, Supplementary Figs. 3, 56–57 and 63–64), which are rare in the tephra from the 3 July and 28 August paroxysms (Fig. 2c) and absent (subgroup 2B) from the 25 June ejecta. Abundant resorbed diopsidic and augitic cores texturally and compositionally similar to our subgroup 2B and 1C crystals were reported in 2003–2017 products^3,28,38,39,41,42^, and in ejecta erupted since at least 1900^5,22,26,28^. They were interpreted as portion of a high ^87^Sr/^86^Sr crystal mush formed since 2.5 ka ago located at the base of the *hp*-reservoir^36,38^^,^ (i.e., >3 km depth) or encompassing both reservoirs^21,44^. Clinopyroxene and plagioclase antecrysts are periodically remobilised from the bottom by the ascent *lp*-magma throughout this period, and recycled from the top by sinking of the dense degassed *hp-*magma left in the conduit during the normal Strombolian activity^28,30,36,38^. Accordingly, we interpret the resorbed diopsidic and augitic cores of subgroups 2B and 1C, respectively, as antecrysts remobilised from this long-lived^28,38^ extant crystal mush.

In the 2019 products, about half of the handful of crystals of subgroup 1C (2% of the total, Fig. 2c, Supplementary Figs. 56–57) have resorbed augitic cores with diopsidic overgrowth and augitic rims. Resorbed diopsidic core (i.e., subgroup 2B) are slightly more abundant (4%), bringing at ~5% the total proportion of clinopyroxene with resorbed cores (Fig. 2c). The proportion of diopsidic clinopyroxene antecrysts was estimated at ~33% for the 2003–2017 products^3^, which also include normal and major activity, demonstrating that the proportion of clinopyroxene antecrysts in 2019 ejecta was significantly lower than in the 2003–2017 period. As our focus is on the clinopyroxene population, only a less direct comparison can be drawn from the proportion of clinopyroxene and plagioclase antecrysts in the products of the 5 April 2003 paroxysm estimated at ~17% with an increase of ~8% in a lava erupted after 5 April 2003 paroxysm^38^. However, considering that the total crystallinity of both *hp*-scoriae and *lp*-pumice has remained relatively constant since at least A.D. 776-1350^27,28^ and that resorbed antecryst augitic and diopsidic cores are reported as a common feature of 1984-1996 ejecta^28^, our data suggest that the proportion of clinopyroxene antecrysts in the eruptive products of summer 2019 is also significantly lower than stretching back to at least 1984. Notably, resorbed augitic cores are not reported among the large clinopyroxene antecryst population examined for the 2003–2017 period^3^, although their presence is noted in the pumice of 5 April 2003 paroxysm and 2007 lavas^39,41,42^. Resorbed augitic cores are also less abundant than the diopsidic core in the scarce antecryst population in 2019 products suggesting a greater contribution from the deeper diopsidic-dominated portion of the crystal mush. Thus, at least since 2003 (unfortunately similar quantitative data are unavailable for older periods of activity) clinopyroxene has recorded a higher degree of remobilisation from a diopside-dominated mush which, we suggest, forms the lower portion of the mushy system (Fig. 7).

The overall scarcity of crystals with resorbed cores indicates that the dynamics of Stromboli during June-August 2019 involved limited interaction of ascending magma with extant mush, contrasting with the 2003–2017 period of activity (Fig. 7) and, possibly also, for activity since at least 1984 if not for the entire 20^th^ century, as testified by the abundance of antecrysts^5,22,26,28^. Efficient mush disruption and cannibalism has been proposed for the 2003–2017 period when a large proportions of diopsidic antecrysts were entrained by the *lp*-magma^3^ (Fig. 7a). We postulate that the continuous remobilisation of the mush led to a rejuvenated plumbing system in 2019, at least in the central conduit. The paucity of antecrysts in the 2019 paroxysms and their absence during major explosive activity supports the hypothesis of a mush rejuvenation process, with limited interaction between the ascending *lp-*recharge magma and the mush (Fig. 7b).

Furthermore, such a model is supported by timescales from subgroup 2B crystals and those with resorbed cores from subgroup 2C (Fig. 5c, Supplementary Figs. 56–57 and 63–64, Table 1), which indicate that the resorbed antecrystic (diopsidic and augitic) cores were remobilized between 5 ± 2 months and 6 ± 2 years before eruption, with the majority of crystals recording timescales between 2 and 6 years. These timescales correspond to approximately mid 2013 to mid-2017, with only one corresponding to early-mid 2019. While acknowledging we have only four estimates, we believe our calculated timescales are significant given the low proportion of antecrysts encountered in the 2019 products. Additionally, these timescales strongly contrast with those calculated for antecryst remobilisation during the 2003–2017 period^3^, which range from 4 to 182 years, including the paroxysmal events of 2003 and 2007 (Fig. 5a, b). Our data indicate that the majority of the 2019 clinopyroxene antecrysts were remobilised from the crystal mush by “older” mafic magma recharge during 2013-2017. Accordingly, there was very limited interaction between ascending *lp*-magma and crystal mush, or at least with the portion of the mush producing resorbed diopsidic and augitic antecrystic cores. This contrast sharply with the documented significant remobilisation of diopsidic antecryst cores in 2003–2017^3^, suggesting that cannibalisation of the deeper diopsidic-dominated portion of the mushy system, generated a rejuvenated plumbing system where *lp-*magma is efficiently permeating the mush without significant clinopyroxene antecryst remobilisation (Fig. 7).

As discussed above, zoned 2 A diopsidic crystals indicate timescales from a few months to a few days (Fig. 5c, Supplementary Figs. 59–62, Table 1) and their cores may have higher Cr and Ni concentrations (Fig. 3), resorption textures (Supplementary Figs. 3 and 60–61) or normal compositional zoning (Supplementary Figs. 59–60). Rarely observed homogenous or slightly zoned diopside crystals (subgroup 2A) at Stromboli^42,45^, are interpreted to crystallise in the *lp* magmatic environment, highlighting active crystallisation in a deeper reservoir where new crystal mush may build (Fig. 7).

### Implications for forecasting changes in eruptive style at basaltic volcanoes

Our data indicate an active role for the deeper mushy magmatic system in triggering the paroxysms at Stromboli in July–August 2019, not only through volatile supply^19^, but also through mafic magma ascent and recharge(s). Remarkable agreement exists between our calculated timescales for *lp* mafic magma injections into the shallow system and precursory monitoring signals before the two 2019 paroxysms.

**Fig. 8:**
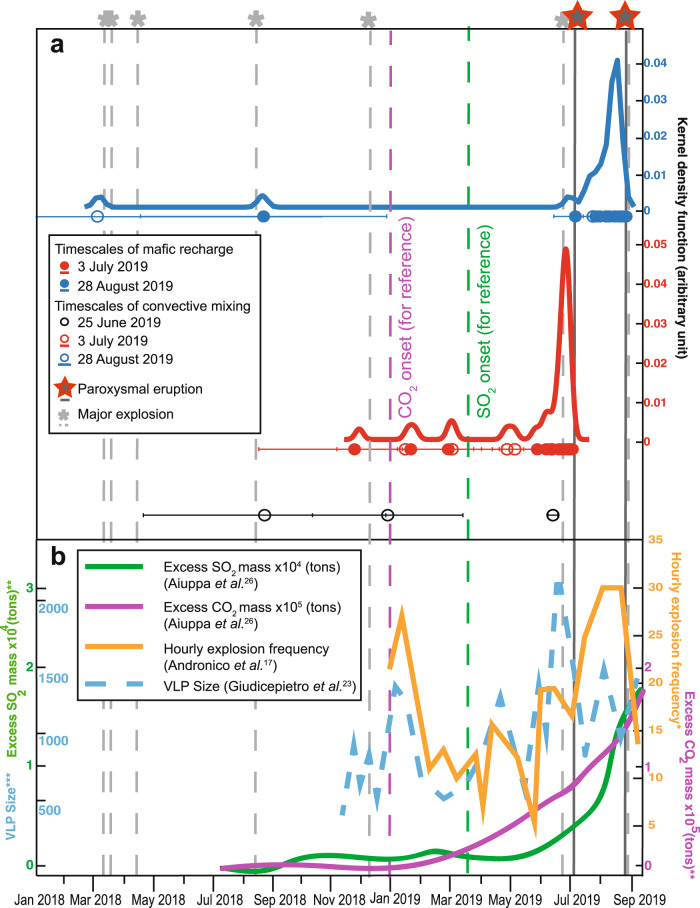
Relationship between timescales of mafic recharge events and monitoring data for the 2018- 2019 period. **a** Kernel density estimates of timescales of magma recharge (subgroups 1B, 1C and Group 2; filled circles) and convective mixing in the shallow *hp-*reservoir (subgroup 1A; open circles) during the January 2018—August 2019 period for the two paroxysmal events (3 July, red curve and circles and 28 August blue curve and circles) are shown alongside the timescales determined for the major explosion on 25 June 2019 (black circles). Timescales of convective mixing for the 25 June major explosion are shown as single data (black open circle) due to the low density of data. **b** July 2018—September 2019 variation of excess of SO_2_ and CO_2_ flux (green and purple line respectively) as calculated by Aiuppa et al.^19^. January—September 2019 variation of hourly explosion frequency (orange line) data from Andronico et al.^10^. November 2018-September 2019 VLP size (amplitude in counts) time series (dashed cyan line) data from Giudicepietro et al.^16^. Dashed vertical light grey lines marked with light grey asterisks: major explosions between January 2018 and September 2019 (data from INGV^46^); continuous vertical dark grey lines marked with red and grey starts: 3 July and 28 August 2019 paroxysmal events.

The kernel density function for the 3 July paroxysm (red line and symbols in Fig. 8a) illustrates that mafic recharge started ~7 months pre-eruption with escalating activity from June 2019. Strikingly no corresponding timescales are recorded in the 28 August 2019 samples (blue line and symbols in Fig. 8a). Instead, clinopyroxenes predominantly record mafic recharge event(s) from ~4 July up to a few days before the 28 August paroxysm. This demonstrates complete evacuation of the shallow system, at least with respect to the *lp*-magma, which triggered the first paroxysm on 3 July 2019, before being refilled and re-destabilised by newly injected recharge *lp*-magma(s) soon after the first paroxysm and up to a few days before the 28 August event, eventually triggering the second paroxysm. This new model differs profoundly from previous models, that linked the 2019 paroxysms to gas flux blockage in the shallow conduit occurring a few minutes pre-eruption, with undegassed *lp*-recharge magma merely representing passive upward migration of a gas slug restoring the conduit gas flux^17^. From gas data, a mafic recharge trigger mechanism (bottom-up) was suggested for the 3 July paroxysm while a top-down mechanism (i.e., depressurisation due to the preceding lava flow and passive uprising of *lp*-magma) was proposed for the 28 August paroxysm^19^. By contrast, we provide strong evidence for the arrival of mafic magma(s) up to a few days before both paroxysms. In addition, the lack of overlapping short timescales between the 3 July and 28 August paroxysms suggests the arrival of distinct batches of gas-rich *lp*-magmas before the two events pointing to bottom-up mechanism also for the 28 August paroxysm.

The sequence of events highlighted here coincides well with monitoring data^10,19^ (Fig. 8b). Increased excess SO_2_ and CO_2_ flux^19^ (green and purple lines in Fig. 8b) correspond to an increasing density in the timescales of mafic injection recorded by clinopyroxenes from the 3 July paroxysm. The first peak of the kernel distribution for the 3 July event represents a timescale of 224 ± 98 days and subsequent peaks correspond to timescales of 165 ± 65 and 128 ± 52 days before eruption (Table 1). These latter timescales agree well with the increasing CO_2_ and SO_2_ fluxes ~180 days and ~120 days pre-eruption^19^. The emerging view is that, contrary to the previous model^19^, magma and gas remained coupled during several months in the build-up to the 3 July paroxysm until a few days before eruption. Chaotic mixing in the shallow reservoir, recorded by subgroup 1A crystals, contemporaneously with the mafic recharge, shows the perturbation of the shallow *hp-*reservoir by the arrival of deeper undegassed *lp*-magma. A similar scenario is also envisaged for the 28 August paroxysm (Fig. 8b).

The hourly explosion frequency^10^ (orange line in Fig. 8b), reveals that the sudden increased explosivity from February-March 2019 coincides with an increasing density of recharge timescales. Furthermore, the highest peaks in the kernel distributions recorded by clinopyroxenes match perfectly with the recorded periods of high to very high levels of normal activity both in terms of explosive frequency and intensity observed from 9 June 2019, ~1 month before the 3 July paroxysm, and lasting until 20 September 2019^10^. The size and amplitude of VLP (very-long period) seismic signals (dashed blue line in Fig. 8b) significantly increased between November 2018 and January 2019, with a strong change in the VLP activity also occurring one month before the 3 July paroxysm^16^. The rapid response of the shallow system to the injections of *lp*-magma is interpreted to result from a more direct link between the two reservoirs, allowing the *lp-*magma permeating more efficiently the lower portion of the mush and resulting in increased explosive activity and changes in the VLP activity. Our model also agrees well with CO_2_ soil degassing^18^, which significantly increased since 2016 and culminated with the 2019 paroxysms, and with our observations^46^ of more variable eruptive activity since September 2019, where at least eight major explosions, intense spattering episodes and lava overflows have occurred.

In conclusion, our investigation of Stromboli’s 2019 paroxysms reveals a key role for batches of undegassed *lp*-magma recharge arriving in the shallow reservoir up to a few days before these events. Our data indicate a rejuvenated plumbing system where *lp-*magma permeates more efficiently the lower diopside-dominated portion of the mush allowing a direct linkage between deeper (*lp*) and shallow (*hp*) reservoirs and limited interaction between *lp-*recharge magma and extant crystal mush (Fig. 7). This sustains the current variability of eruptive styles with near immediate eruptive response to mafic magma recharge. The remarkable agreement between our calculated recharge timescales and the observed variation in time of various monitoring signals strongly supports such a model (Fig. 8).

The processes observed at Stromboli are likely common to other persistently active basaltic volcanoes (e.g., Etna, Italy;^47^ Volcan de Fuego, Guatemala;^48^ Kilauea, Hawaii^49^) that show similar sudden shifts from mild Strombolian explosions to violent paroxysms. Stromboli’s post-2019 activity, characterised by an efficient connection between deeper and shallower reservoirs, has important implications for forecasting future paroxysmal eruptions and interpreting volcano monitoring data. We suggest that for well-monitored volcanoes, where detailed knowledge of the tempo and processes governing the plumbing system exists, devising a high-frequency petrological monitoring protocol can help discriminate gas-driven and magma-driven eruption triggering. Such information can be integrated with volcanological, geophysical, and geochemical data for a fast response during a volcanic unrest phase or crisis.

## Methods

### Analytical techniques

***Bulk rock analyses*** for major and trace elements were conducted at Actlabs (Activation Laboratories Ltd., Ancaster, Ontario, Canada). Results are reported in Supplementary Table 1. Major elements were analysed by lithium metaborate/tetraborate fusion—ICP-OES (inductively coupled plasma optical emission spectrometry) and run on a Thermo Jarrell Ash ENVIRO II ICP. FeO was determined through titration, using cold acid digestion of ammonium metavanadate, and hydrofluoric acid in an open system. Ferrous ammonium sulphate was added after digestion and potassium dichromate was the titrating agent. Trace elements were measured by lithium metaborate/tetraborate fusion—ICP-MS (inductively coupled plasma mass spectrometry). Fused samples were diluted and analysed using Perkin Elmer Sciex ELAN 6000, 6100, or 9000 instruments. A bulk rock quality control table measured against certified standards used for bulk rock analyses at the Activation Laboratories Ltd. (Actlabs) is provided in the supplementary material (Supplementary Table 5).

***Automated SEM-EDS maps*** were undertaken at Imaging and Analysis Centre (IAC) at the Natural History Museum in London (NHM) on a Tescan Integrated Mineral Analyser (TIMA) with four EDAX EDS detectors using a field emission source at 25 kV and 19.75 beam intensity. Analyses were carried out on a 5 μm pixel spacing with 1000 X-ray counts collected per pixel amounting to around 12 million analysis points and 12 billion X-ray counts per sample. Mineral classifications were manually assigned using only spectra acquired from this sample set.

***Back Scattered electron (BSE) images*** were acquired with the Jeol IT500 FEG-SEM and the FEI QUANTA 650 FEG-SEM, at IAC-NHM, operating at 15–20 and 15 KeV electron current, and 10–11 and 15 mm WD (working distance), respectively. The spatial resolution of the SEM at our analytical condition was tested imaging the Richardson Test Slide (model 80302, serial n. 10461) for the 53 and 50 nm resolution. This test sample is routinely used to test the spatial resolution of SEM. We performed the test at different voltages (20, 15, 10 and 8 kV), working distances (WD) of 8 and 15 mm and different beam spot sizes. The images were all taken at the FEI Quanta 650 FEG SEM, where the spot size is a numerical value and we typically operate between 3 and 5. The results of the test are reported in Supplementary Figures 65–67, where it is visible that the resolution remains unchanged under all the different tested conditions. Therefore, at our operation conditions of 20–15 kV and 15–10 mm WD we have a resolution better than 50 nm, which is four times smaller than a normal pixel size of 0.2 μm and on average 20–40 times smaller than our smallest diffusion profiles (~1–2 μm).

To obtain the best results for diffusion modelling and minimise the effect of not sectioning perpendicular to the core-rim boundary, careful screening of the crystals in terms of reducing the orientation effect was carried out in the analysed thin sections. Diffusion profiles were performed along the direction perpendicular to the (100) or (010) crystallographic plane, according to ref. 40.

***Electron microprobe*** Chemical composition of clinopyroxenes and matrix glasses alongside high-resolution core-rim compositional profile, ~3–5 µm step size, of major and selected trace elements of clinopyroxene were obtained using a Cameca SX 100 electron microprobe at IAC-NHM. The Cameca SX100 is equipped with five WDS (wavelength dispersive) spectrometers and one EDS (energy dispersive) spectrometer. It was operated at 20KeV and 20 nA with a focused beam for clinopyroxenes and a defocused 10 μm spot size for matrix glasses. Sodium was measured as first elements and counted for 10 s, all the other elements (Si, Mg, Al, Ca, Ti, Cr, Mn, Fe, Ca, and Ni) were measured for 20 s. Matrix effects were corrected using the Cameca PAP built-in protocol^50^. Detection limits for most elements were <0.2 wt.% and standard deviation of the analyses was typically <0.5 wt.%. A quality control table of measured against certified standard used for electron microprobe analyses of clinopyroxene at NHM-IAC laboratories is provided in the Supplementary Material (Supplementary Table 6). From each profile, representative analysis of the core-mantle-rim portion of the crystal were extracted and used to discuss the compositional variability and determine the *P-T-*H_2_O contents of clinopyroxenes. Data are reported in Supplementary Tables 2 and 3.

***Trace element analysis*** Concentrations of trace elements in pyroxenes and glasses were determined by laser ablation inductively coupled plasma mass spectrometry (LA-ICPMS) spot analysis at the IAC-NHM and at the Institute of Geochemistry and Petrology, ETH Zürich. The analyses at NHM were performed using a 193 nm Iridia laser ablation system (Teledyne Photon Machines, Bozeman, MT, USA) coupled to an Agilent 8900 ICP-QQQ-MS. The Iridia is equipped with a 193 nm MLase short pulse (<7 ns) laser and the low-dispersion Cobalt (2-volume) ablation chamber^51^. The laser was operated using an 8 Hz repetition rate, a fluence of ~3.5 J cm^−2^ and a spot diameter of 35 µm. Samples were ablated in a He atmosphere (c. 0.5 l min^−1^) and the ablated aerosol was mixed with Ar (c. 0.8 l min^−1^) before being introduced to the interface of the ICP-MS. The ICP-MS was calibrated for maximum sensitivity, while minimising oxide production (^232^Th^16^O^+^/^232^Th^+^ <0.15 %) and laser induced fractionation (^232^Th^+^/^238^U^+^ > 90%) using a line scan of the NIST SRM 612 at the start of each analytical session. Analyses at ETH were conducted using a 193 nm Resonetics ArF excimer laser coupled to a Thermo Element XR ICPMS within. Analytical spot sizes were 43 µm with the energy of laser output maintained at ~3.5 J/cm^2^.

For all trace element determinations, a set of two NIST SRM 612 reference glass was measured every 25 unknowns to calibrate and monitor for instrumental drift. A further set of two GSD-1g reference glass was measured every 25 unknowns as part of a long-term lab reproducibility. Data reduction was performed using Iolite v. 3.61^52^ (NHM) and MATLAB-based programme SILLS^53^ (ETH) using the Ca concentration determined by electron microprobe as the internal standard. For each analysis a baseline of 25 s was measured prior to 40 s of ablation. The following isotopes were measured: ^24^Mg, ^27^Al, ^43^Ca, ^45^Sc, ^47^Ti, ^51^V, ^52^Cr, ^55^Mn, ^60^Ni, ^66^Zn, ^89^Y, ^90^Zr, ^139^La, ^140^Ce, ^141^Pr, ^146^Nd, ^147^Sm, ^153^Eu, ^157^Gd, ^159^Tb, ^163^Dy, ^165^Ho, ^166^Er, ^169^Tm, ^172^Yb, and ^175^Lu. Dwell times for each element was 10 ms, except for ^24^Mg, ^27^Al (5 ms); ^52^Cr, ^60^Ni, ^66^Zn, ^153^Eu, ^159^Tb, ^165^Ho, ^169^Tm, ^175^Lu (20 ms). All trace elemental determinations are considered to have precision better than 5% relative, based on homogeneous standard reproducibility where abundances are significantly above (typically 3x) the limits of detection. Elements of particular interest to this study (e.g. La, Yb, Zr, Sc, Cr, Fig. 6) all occur at abundances an order of magnitude or greater above typical limits of detection for these analytical conditions. Data are reported in Supplementary Table 4.

***LA-ICP-MS trace element mapping of clinopyroxene*** grains was conducted over six analytical sessions at IAC-NHM (see above for instrument details). Samples were ablated in a He atmosphere (c. 0.8 l min^−1^) and the ablated aerosol was mixed with Ar (c. 0.8 l min^−1^) and introduced to the interface of the ICP-MS using the Aerosol Rapid Introduction System (ARIS; Teledyne Photon Machines, Bozeman, MT, USA; Van Acker et al., 2016). For all mapping experiments aerosol dispersion (washout time) was minimised by optimising the full width of a single laser pulse at 10% of the maximum peak intensity (FW0.1 M) to <5 ms using a live signal monitor (LA Monitor) in the HDIP software^54^ to improve signal sensitivity and reduce imaging time.

Laser ablation compositional maps were acquired through a series of adjacent line scans, obtained by moving the stage at constant speed under a fixed ablation site. The effect of analytical artefacts (aliasing) caused by the interaction of the pulsed laser signal and the total sweep cycle time^55^^,^ was minimised by adjusting the total sweep cycle of the ICP-MS and laser parameters (frequency, scan speed, spot size) using the LA Optimise tool in HDIP (based on equations of Van Marderen et al.^51,55^ and Šala et al.^56^).

All mapping experiments were carried out using a 3 × 3 µm square beam, a fluence of 3.5 J cm^−2^, a repetition rate of 375 Hz and a scan speed of 93.75 µm s^−1^, corresponding to 12 laser pulses per 3 µm pixel. Six isotopes were measured in no-gas mode, with a total ICP-MS sweep cycle of 32 ms. A line scan of a representative pyroxene was used to optimise dwell times for each measured isotope to ensure sufficient signal/noise was achieved on the low abundance isotopes at the small spot size. Isotopes measured in order of increasing dwell time are: ^24^Mg, ^27^Al (0.5 ms); ^47^Ti (3 ms); ^52^Cr (5.2 ms); ^43^Ca (5.4 ms); ^60^Ni (7.4 ms). Each analytical session comprised the acquisition of pyroxene compositional maps during a single ICP-MS acquisition lasting between c. 2 and 9 hours. Individual compositional maps took between c. 30 minutes and c. 3.5 hours to gather. A gas blank was gathered between each successive line scan. NIST SRM 612^57^ was measured as line scans every ~30 minutes for calibration and drift correction. Larger images were separated into multiple blocks by reference materials to better constrain instrumental drift.

Image processing and quantification was carried out using HDIP (v. 1.6). After synchronising the timestamps of the laser with the ICP-MS signal, the background of each isotope was calculated during periods the laser was not firing and subtracted from each isotope measured. Drift correction was applied to the measured counts of each isotope using the NIST SRM 612. Pyroxene grains were isolated from each created image based on measured counts of ^43^Ca using the labelled segments tool in HDIP. Glass reference materials BCR-2g and GSD-1g were used as secondary reference material. Trace element concentrations in pyroxenes were calculated on a pixel-by-pixel basis using the Sum Normalisation tool in HDIP using the Ca concentration obtained from EMPA as an internal standard. For crystals zoned in Ca concentration each zone was isolated based on the BSE image using the labelled segments tool in HDIP and the appropriate Ca concentration was used as the internal standard for each zone.

#### Clinopyroxene calculation scheme and modelling

Following the calculation scheme of Putirka et al.^58^, the total number of oxygens in the formula of clinopyroxene is fixed to six. Then, it is assumed that *X*_*Si*_ + *X*_*TAl*_ = 2.00 in T-site, where *X*_*i*_ is the cation fraction of the element of interest *i*. Jd (jadeite) = *X*_*M1Al*_ if *X*_*M1Al*_ < *X*_*Na*_ or, alternatively, Jd = *X*_*Na*_. CaTs (Ca-Tschermark) = *X*_*M1Al*_ – Jd, CaFeTs = (*X*_*M1Fe3+*_ + *X*_*Cr*_)/2, and CaTiTs = (*X*_*TAl*_ – CaTs)/2 (if *X*_*TAl*_ > CaTs). Di + Hd (diopside + hedenbergite) = *X*_*Ca*_ – CaTs – CaFeTs – CaTiTs and En + Fs (enstatite + ferrosilite) = [(*X*_*TOTMg*_ + *X*_*TOTFe*_) – (Di + Hd)]/2. A charge balance equation is applied to all the analyses for determining *X*_*Fe3+*_ [i.e., (*X*_*M1Al*_ + *X*_*M1Fe3+*_ + *X*_*Cr*_ + 2*X*_*Ti*_) = (*X*_*TAl*_ + *X*_*Na*_)]. *X*_*M1Mg*_ and *X*_*M1Fe2+*_ are calculated as: *X*_*M1Mg*_ = *X*_*TOTMg*_/(*X*_*TOTMg*_ + *X*_*TOTFe*_) × (1 – *X*_*M1Al*_ – *X*_*Cr*_ – *X*_*Ti*_) and *X*_*M1Fe2+*_ = *X*_*TOTFe*_ – *X*_*M2Fe2+*_, where *X*_*M2Fe2+*_ = 1 – (*X*_*Ca*_ + *X*_*M2Mg*_) and *X*_*M2Mg*_ = *X*_*TOTMg*_ – *X*_*M1Mg*_, respectively.

The error associated with estimates of clinopyroxene-based thermometer (*P*-H_2_O-independent equation with error of ±6 °C^59^), barometer (*T*- H_2_O-independent equation with error of ±136 MPa^60^), and hygrometer (*P-T*-dependent equation with error of 0.5 wt.%^61^) is minimised by testing for equilibrium between clinopyroxene and melt (Supplementary Fig. 5). Poorly differentiated matrix glasses of *lp*-products and their bulk rock analyses that are compositionally similar to these matrix glasses are equilibrated with diopside. Conversely, more evolved matrix glass compositions of *hp*-products are equilibrated with augite. The use of bulk rock compositions is restricted to *lp*-products, in agreement with the textural observation that their crystal content is only ~5 vol.% and implying that bulk rock and matrix glass compositions are almost identical. The most likely *P*-*T*-H_2_O conditions used for probability density functions are determined by a mathematical procedure based on minimisation of crystal-melt equilibrium parameters (Supplementary Fig. 5). Thus, barometric, thermometric, and hygrometric estimates are adjusted within the calibration errors of the predictive equations in order to minimise (1) the difference between measured vs. predicted diopside + hedenbergite components (*Δ*DiHd from Mollo et al.^62^) and (2) the difference between measured vs. predicted clinopyroxene-melt partition coefficients for Na and Ti (*Δ*D_Na_ and *Δ*D_Ti_ from Blundy et al.^63^ and Mollo et al.^33^, respectively). By integrating these thermodynamic-based models (Supplementary Fig. 5), the near-equilibrium state of clinopyroxene is verified when values of *Δ*DiHd, *Δ*D_Na_, and *Δ*D_Ti_ approaches the ideal equilibrium condition of *Δ* = 0 (cf. Mollo et al.^33^).

#### Fe-Mg elemental diffusion modelling

Timescales of mafic recharges, convective mixing and antecrysts remobilisation from the crystal mush were determined using NIDIS (Non-Isothermal Diffusion Incremental Step) model of Petrone et al.^40^. The NIDIS model considers how temperature changes during the growth of a zoned crystal modulate the rate of diffusion, allowing to calculate the storage time in different magmatic environments characterised by different temperatures and chemical compositions. In the case of clinopyroxenes, the model uses grey values extracted from BSE images as proxy for Mg# (i.e., Fe-Mg elemental diffusion) and the diffusive profile for each compositional boundary is modelled at the temperature of the liquid in equilibrium with that specific composition^40^. For crystals like those of subgroup 1C (Fig. 2, Supplementary Figs. 57–58), the NIDIS model calculates the total residence time (*Δt* = *Δt*_*1*_ + *Δt*_*2*_, labelled as diopsidic band in Fig. 5c) by estimating the temporal interval between the formation of the diopsidic band (*Δt*_*1*_) and the eruption (*Δt*_*2*_). The partial timescale *Δt*_*1*_ corresponds to the partial diffusive time after the formation of the internal compositional boundary (i.e., augitic core—diopsidic band) at the higher *T* of diopsidic band. *Δt*_*2*_ corresponds to the partial diffusive time after the formation of the external compositional boundary (i.e., diopsidic band —augitic overgrowth) until eruption, and it is calculated at the *T* of augitic overgrowth. According to NIDIS model, *Δt* is calculated using a backward approach starting from the last formed compositional boundary (*Δt*_*2*_) and proceeding inward towards the more internal boundary (*Δt*_*1*_, see Petrone et al.^30,40^ for further details). Residence time in the last magmatic environment before eruption was calculated for subgroups 1B and 2B crystals (Figs. 2 and 5, Supplementary 15–55 and 63–64, Table 1), adapting the NIDIS model^30,40^. The overgrowth of crystals records the last magmatic event prior to eruption and in the case of subgroup 1B crystals the diopsidic overgrowth suggests a mafic recharge prior to eruption. It has been argued^30^ that if the mafic recharge event occurred shortly (i.e., from a few days to a few months) prior to eruption, it represents the immediate eruption trigger, pointing to a bottom-up mechanism (i.e., an active role of the deeper system as opposed to a passive role driven by the shallow conduit dynamics^19^). The diopsidic overgrowths of subgroup 1B clinopyroxenes record the timescales of mafic recharge event(s), as confirmed by the core-rim compositional profiles (Supplementary Figs. 15–55) where the compositional changes of Ca and Al, the slow diffusive elements, preserve sharp variations suggestive of diffusion profiles due to magma mixing events as opposed to chemical variation due to crystal fractionation (see § *Mafic recharge as eruption trigger of 2019 paroxysms*). The augitic overgrowth of 2B events provided time constraints on remobilisation of resorbed diopsidic antecryst cores from the crystal mush and their subsequent storage in the shallow *hp* magmatic environment (see § *The lifecycle of basaltic crystal mush*). Time constraint on antecryst remobilisation form the crystal mush have been also obtained from resorbed augitic cores surrounded by diopsidic band of some subgroup 1C crystals (Supplementary Figs. 56–57). As discussed in the text (see § *Convective mixing regime controlling magma dynamics*), we have calculated pre-eruptive timescales for subgroups 1A and 2A crystals (Figs. 2 and 5c, Supplementary Figs. 6–14, 59–62). These diffusive timescales set constraints on the convective mixing processes in both the shallow *hp* and the deeper mafic *lp* magmatic environments.

The greyscales values of zoned boundaries of clinopyroxenes were extracted by BSE-photomicrographs using the *greyvalues* script^40^, calibrated against the Fe-Mg (Mg#) chemical profile acquired at the electron microprobe and fitted with the *cratefit* script^40^ to calculate the timescale. Electron beam convolution effect can be important particularly for shorth diffusion profile (i.e., short timescales). To assess if the greyscale profiles were at least in part modified by convolution effect, nine clinopyroxenes with timescales ranging from a few days to years were re-imaged at the FEG-SEM collecting BSE images at 10 kV and 8 kV with the working distance set at 8 mm. These data were compared with data extracted from BSE images taken at higher voltages (15–20 kV) and working distances (10–15 mm) (i.e., the analytical conditions used in this study). Each crystal was imaged under the new conditions (3–6 photos and timescales), but we carefully selected the same area from which the original profile was extracted, reproducing a similar greyscale profile to avoid any further bias. For simplicity we report the calculated timescales at the different conditions (the goodness of the fit of the greyscale profiles measured by the r^2^ were all between 1.00 and 0.99, except for 3 profiles that returned 0.98–0.97). As shown in Supplementary figures 68–69, all timescales calculated for each crystal under different analytical conditions agree within the analytical error of the calculated timescale. Therefore, we can exclude any smoothing of the profile due to convolution effect.

Grey values were acquired along transect normal to the {1 0 0} and {0 1 0} sectors avoiding the *c* axis. According to thermobarometric results and considering 2-sigma standard error of ±16 °C on the estimated temperatures (Fig. 4) Fe-Mg diffusion coefficients were calculated at 1155 and 1175 °C for augitic and diopsidic composition respectively. The values of the pre-exponential factor and activation energy are from Dimanov & Sautter^64^. As the diffusion temperature decreases, the timescale error increases due to variations in activation energy and intra-crystal cation diffusivity^40,65^ (and references therein). In light of these considerations, timescales from this study represent conservative crystal residence times for the magma dynamics at Stromboli. As discussed in Petrone et al.^30^, under the used analytical condition and considering the uncertainty on the fit parameter $\sqrt{4{Dt}}$ (always better than 5% and in most cases better than 2%), the pixel resolution of the BSE-SEM greyscale (better than 0.3 μm) and the values of the diffusion coefficient D, the NIDIS method allows to start detecting change of the diffusion profile from the initial condition after only 1 day (see Supplementary Fig. 6 in Petrone et al.^30^).

## Supplementary information

Supplementary Information

Description of Additional Supplementary files

Supplementary Table 1

Supplementary Table 2

Supplementary Table 3

Supplementary Table 4

Supplementary Table 5

Supplementary Table 6

## Data Availability

The entire set of mineral chemistry data is available at NERC EDS Dataset Petrone CM 2022^66^. All other data are provided in the paper and/or the Supplementary Materials. All clinopyroxenes BSE images, core-rim chemical profiles and calculated timescales are provided in the Supplementary Materials.
