# Supplementary material for: Magma recharge and mush rejuvenation drive paroxysmal activity at Stromboli volcano
Source: Nat Commun. 2022 Dec 13;13:7717. doi: 10.1038/s41467-022-35405-z (PMC9746564; doi:10.1038/s41467-022-35405-z)
Supplement: Supplementary file 1 — Supplementary Information [file 41467_2022_35405_MOESM1_ESM.pdf]

Supplementary Figures for

Magma recharge and mush rejuvenation drive paroxysmal activity at Stromboli volcano.

Chiara Maria Petrone*, Silvio Mollo, Ralf Gertisser, Yannick Buret, Piergiorgio Scarlato, Elisabetta del Bello, Daniele Andronico, Ben Ellis, Alessio Pontesilli, Gianfilippo De Astis, Pierpaolo Giacomoni, Massimo Coltorti, Mark Reagan

*Corresponding author. Email c.petrone@nhm.ac.uk

This PDF file includes:

Supplementary Fig. 1 to 69

Other Supplementary Materials for this manuscript include the following:

Supplementary Tab. 1 to 6 (excel files)

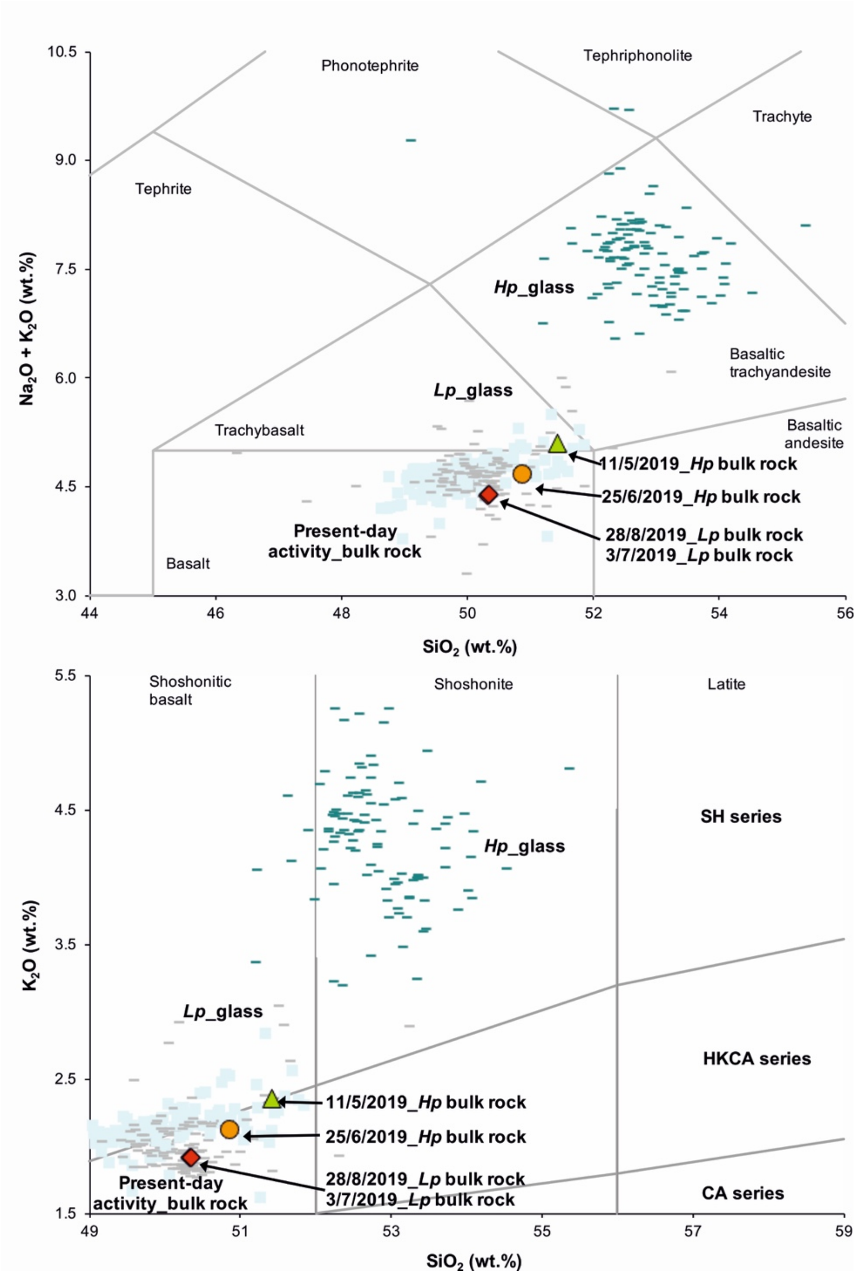

Supplementary Fig. 1.

Classification diagrams for bulk rocks and glassy groundmasses of 2019 Stromboli samples. Bulk rocks: green triangle: *hp*-scoria normal activity 11 May 2019; orange circle: *hp*-scoria major explosion 25 June 2019; red diamond: *lp*-pumices paroxysmal events 3 July and 28 August 2019; cyan square: literature data for the Present-day activity from[1,2]. Glassy groundmasses: grey dash: *lp* glass; green dash: *hp* glass. The compositional bimodality of matrix glasses resulting from the occurrence of less differentiated *lp* glass (i.e., shoshonitic basalt with prevalent high-K calc-alkaline affinity) and slightly more differentiated *hp* glass (i.e., shoshonites with typical shoshonitic affinity) is clearly visible.

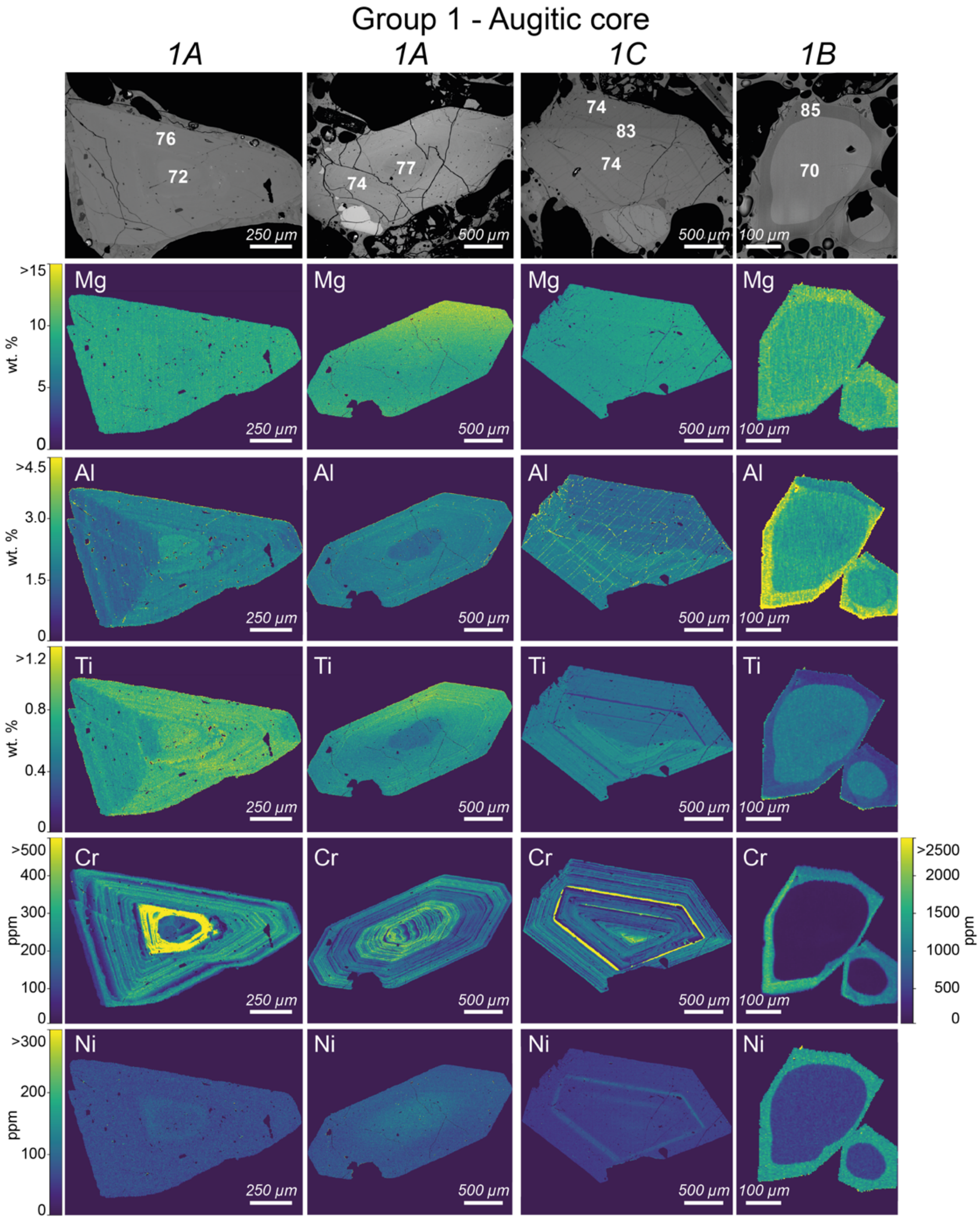

Supplementary Fig. 2.

Major and trace element chemical mapping for 2019 clinopyroxenes. BSE images (top) alongside quantitative chemical mapping of Mg, Al, Ti (wt%), Cr and Ni (ppm) for the 3 subgroups of group 1 clinopyroxenes. Subgroup 1A: first column: Px 30 sample 25062019, 25 June 2019 major explosion; second column: Px12 sample P30-09-02-hp, 3 July paroxysm; subgroup 1C: Px6 sample P47-1, 28 August paroxysm; subgroup 1B: Px11 sample P-44-1-lp, 28 August paroxysm. The scale bar for Cr is on the left for subgroup 1A, 1C and on the right for subgroup 1B.

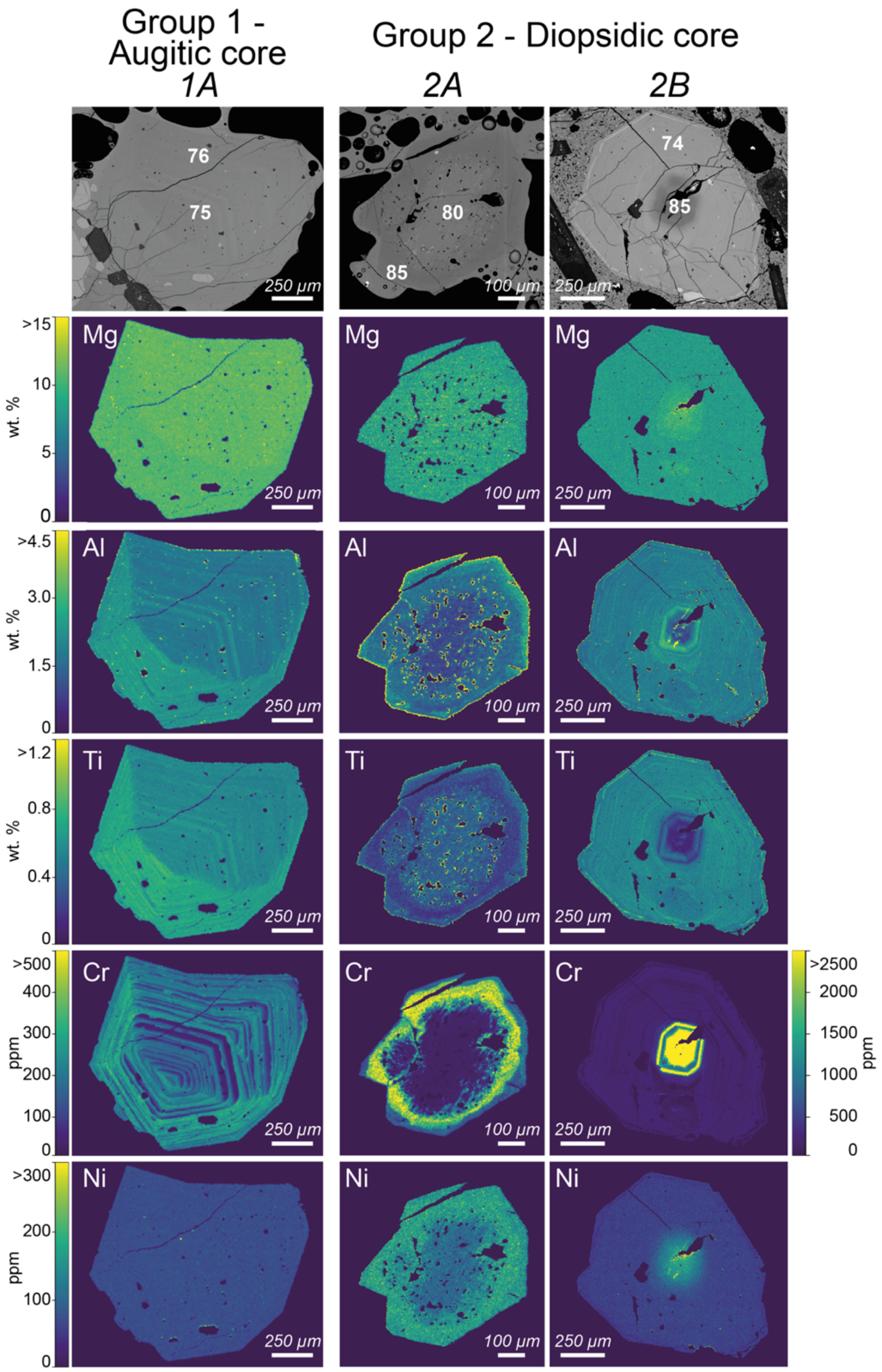

Supplementary Fig. 3.

Major and trace element chemical mapping for 2019 clinopyroxenes. BSE images (top) alongside quantitative chemical mapping of Mg, Al, Ti (wt%), Cr and Ni (ppm) for sub-groups 1A, 2A and 2B clinopyroxenes. Subgroup 1A: Px 2 sample P44-1-hp, 28 August paroxysm; subgroup 2A: Px15 sample P30-07-lp, 3 July paroxysm; subgroup 2B: Px1 sample P27-1-hp, 3 July paroxysm. The scale bar for Cr is on the left for subgroup 1A and 2A and on the right for subgroup 2B.

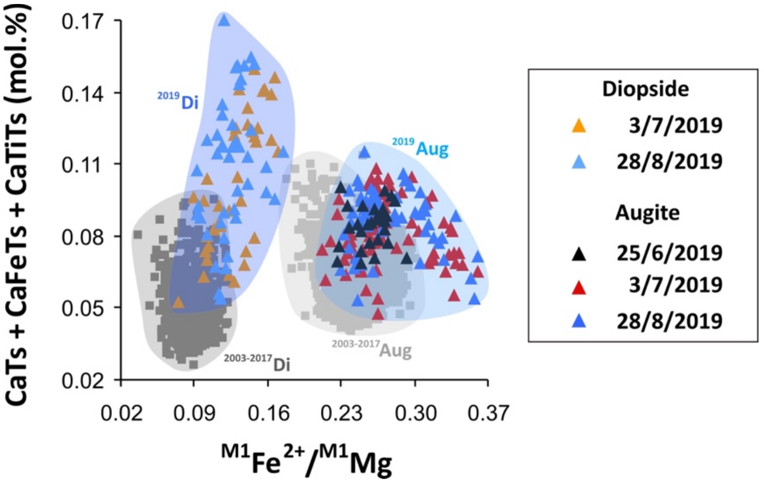

Supplementary Fig. 4.
Site occupancy of clinopyroxene. M1 site occupancy ($^{M1}Fe^{2+}/^{M1}Mg$) versus the sum of Tschermak component [CaTs+CaFeTs+CaTiTs (mol%)] for 2019 Stromboli clinopyroxenes with marked diopside (Di) and augitic (Aug) compositional field for both the 2019 (dark blue sky and cyan respectively) and 2003-2017 (dark and light grey respectively, from Di Stefano *et al.*[2]) clinopyroxene crystals. The entry of highly charged cations into the M1- and M2-sites of 2019 clinopyroxene crystals is due to local charge balance mechanisms in which ^{T}Al replaces for ^{T}Si in T-site, leading to an increasing amount of Tschermak components. The role played by ^{T}Al as charge-balancing cation is to enhance the stability of different local configurations into the clinopyroxene structure, such as $REEMgAlSiO_6$, $CaHFSE^{3+}Al_2O_6$, and $CaScAlSiO_6$ for the $^{T}Si \leftrightarrow {}^{T}Al$ substitution (see Mollo *et al.*[3] and references therein and Fig. 4 in the main text).

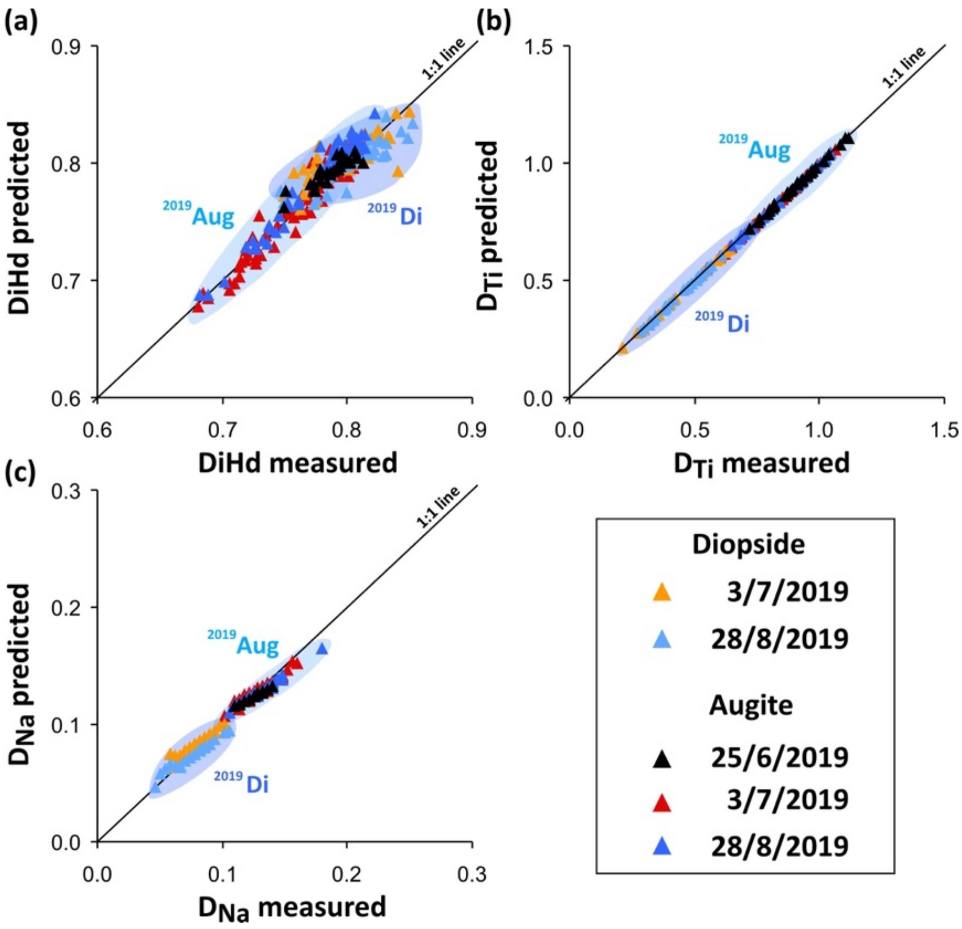

Supplementary Fig. 5.
Predicted and measured (A) diopside - hedenbergite (DiHd) chemical component and clinopyroxene-melt partition coefficients for Ti (B) and Na (C) for the 2019 clinopyroxenes following the scheme of Mollo *et al.*[4] (see Method for further details).

Major 25 June 2019, Sample 25062019 - Px8 – Subgroup 1A

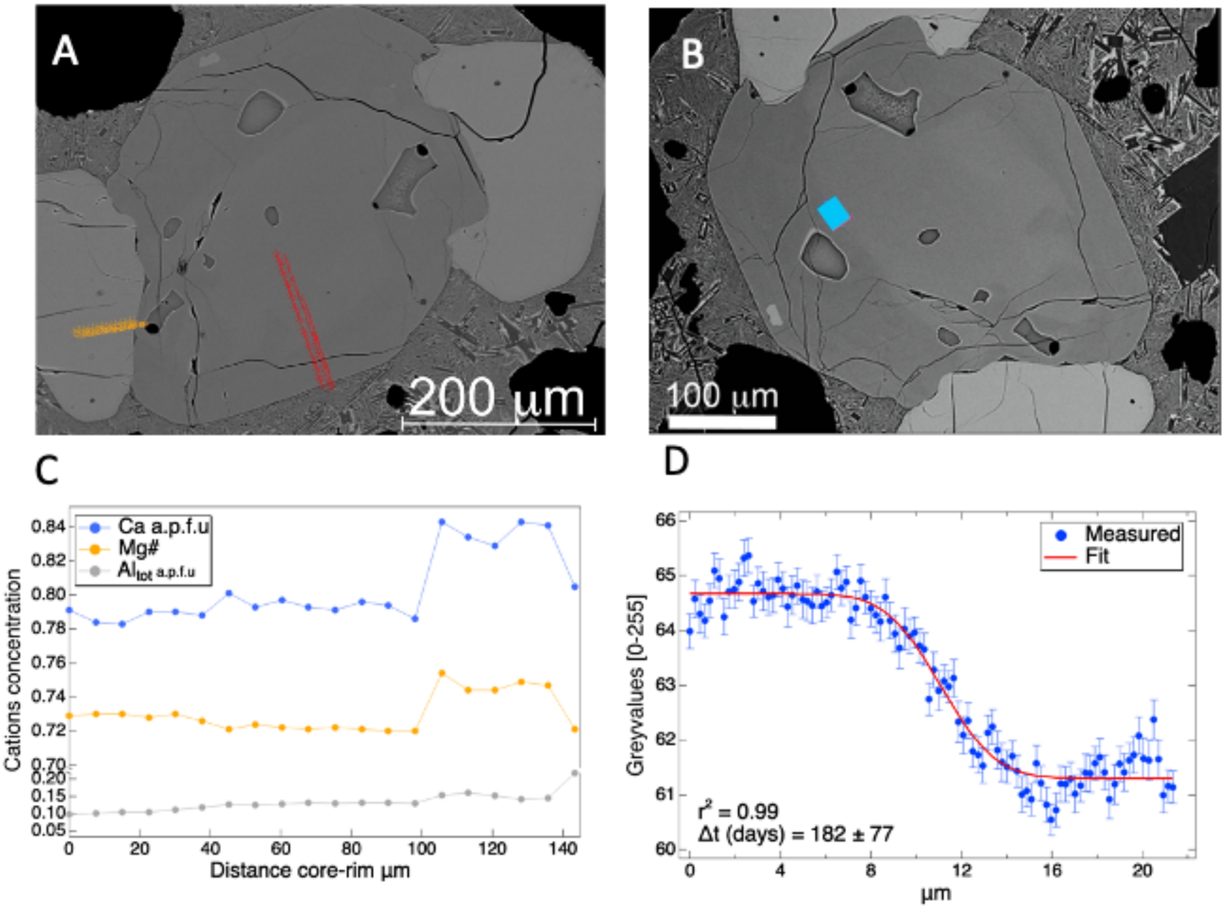

Supplementary Fig. 6.

Clinopyroxene chemical profile and diffusion modeling. (A) SEM-BSE image of the analyzed clinopyroxene. The red line with numbers marks the analyzed chemical profile reported in C. (B) SEM-BSE high resolution image showing the area (light blue area) where the grey scale profile for the diffusion profile has been extracted using the *greyvalues* Matlab script of the NIDIS model from Petrone *et al.*[5]. The red dot on the light blue area marks the initial point of the profile. (C) Variation of Ca (blue dots +line), Al (grey dots + line) (a.p.f.u.) and Mg# (orange dots + line) *vs* distance (µm) along the chemical profile shown in (A). (D) Grey values diffusion profile (blue dots with error bar) *vs* distance (µm) extracted along the light blue area in (B). The red line is the fit of the diffusion modeling calculated using the *createfit* Matlab script of the NIDIS model from Petrone *et al.*[5]. The goodness of the fitting is reported as r^2 and Δt is the calculated timescales (in days or years). The grey values are used as proxy of Mg# in clinopyroxene following Petrone *et al.*[5]. The temperature at which the diffusion has been calculated is reported in Table 1 for each clinopyroxene. See Method and Materials for further details.

Major 25 June 2019, Sample 25062019 – Px16 – Subgroup 1A

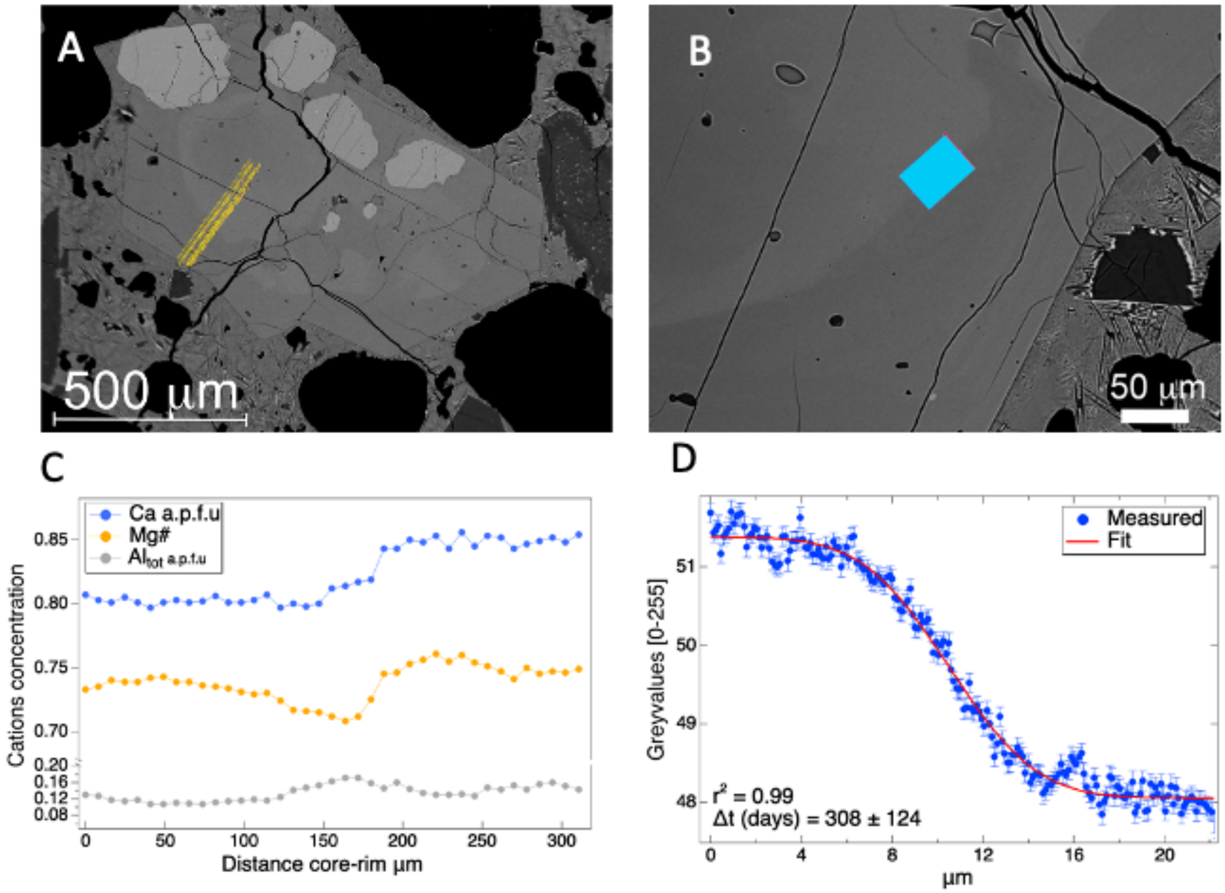

Supplementary Fig. 7.
Clinopyroxene chemical profile and diffusion modeling. (A) SEM-BSE image of the analyzed clinopyroxene. The yellow line with numbers marks the analyzed chemical profile reported in C. (B) SEM-BSE high resolution image showing the area (light blue area) where the grey scale profile for the diffusion profile has been extracted using the *greyvalues* Matlab script of the NIDIS model from Petrone *et al.*[5]. The red dot on the light blue area marks the initial point of the profile. (C) Variation of Ca (blue dots +line), Al (grey dots + line) (a.p.f.u.) and Mg# (orange dots + line) *vs* distance (µm) along the chemical profile shown in (A). (D) Grey values diffusion profile (blue dots with error bar) *vs* distance (µm) extracted along the light blue area in (B). The red line is the fit of the diffusion modeling calculated using the *createfit* Matlab script of the NIDIS model from Petrone *et al.*[5]. The goodness of the fitting is reported as r^2 and Δt is the calculated timescales (in days or years). The grey values are used as proxy of Mg# in clinopyroxene following Petrone *et al.*[5]. The temperature at which the diffusion has been calculated is reported in Table 1 for each clinopyroxene. See Method and Materials for further details.

Major 25 June 2019, Sample 25062019 – Px18 – Subgroup 1A

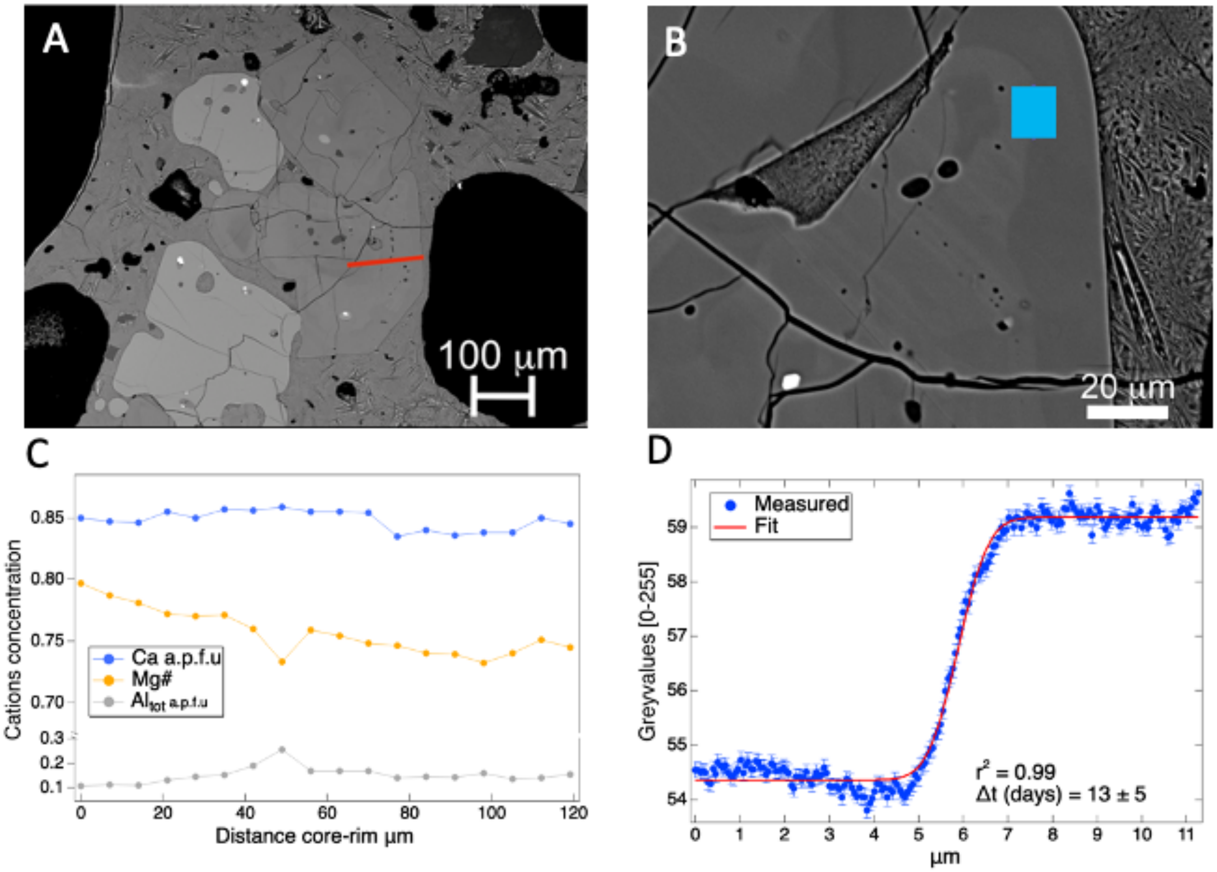

Supplementary Fig. 8.
Clinopyroxene chemical profile and diffusion modeling. (A) SEM-BSE image of the analyzed clinopyroxene. The red line with numbers marks the analyzed chemical profile reported in C. (B) SEM-BSE high resolution image showing the area (light blue area) where the grey scale profile for the diffusion profile has been extracted using the *greyvalues* Matlab script of the NIDIS model from Petrone *et al.*[5]. The red dot on the light blue area marks the initial point of the profile. (C) Variation of Ca (blue dots +line), Al (grey dots + line) (a.p.f.u.) and Mg# (orange dots + line) *vs* distance (µm) along the chemical profile shown in (A). (D) Grey values diffusion profile (blue dots with error bar) *vs* distance (µm) extracted along the light blue area in (B). The red line is the fit of the diffusion modeling calculated using the *createfit* Matlab script of the NIDIS model from Petrone *et al.*[5]. The goodness of the fitting is reported as r^2 and Δt is the calculated timescales (in days or years). The grey values are used as proxy of Mg# in clinopyroxene following Petrone *et al.*[5]. The temperature at which the diffusion has been calculated is reported in Table 1 for each clinopyroxene. See Method and Materials for further details

Paroxysm 3 July 2019, Sample P30-09, hp – Px6a – Subgroup 1A

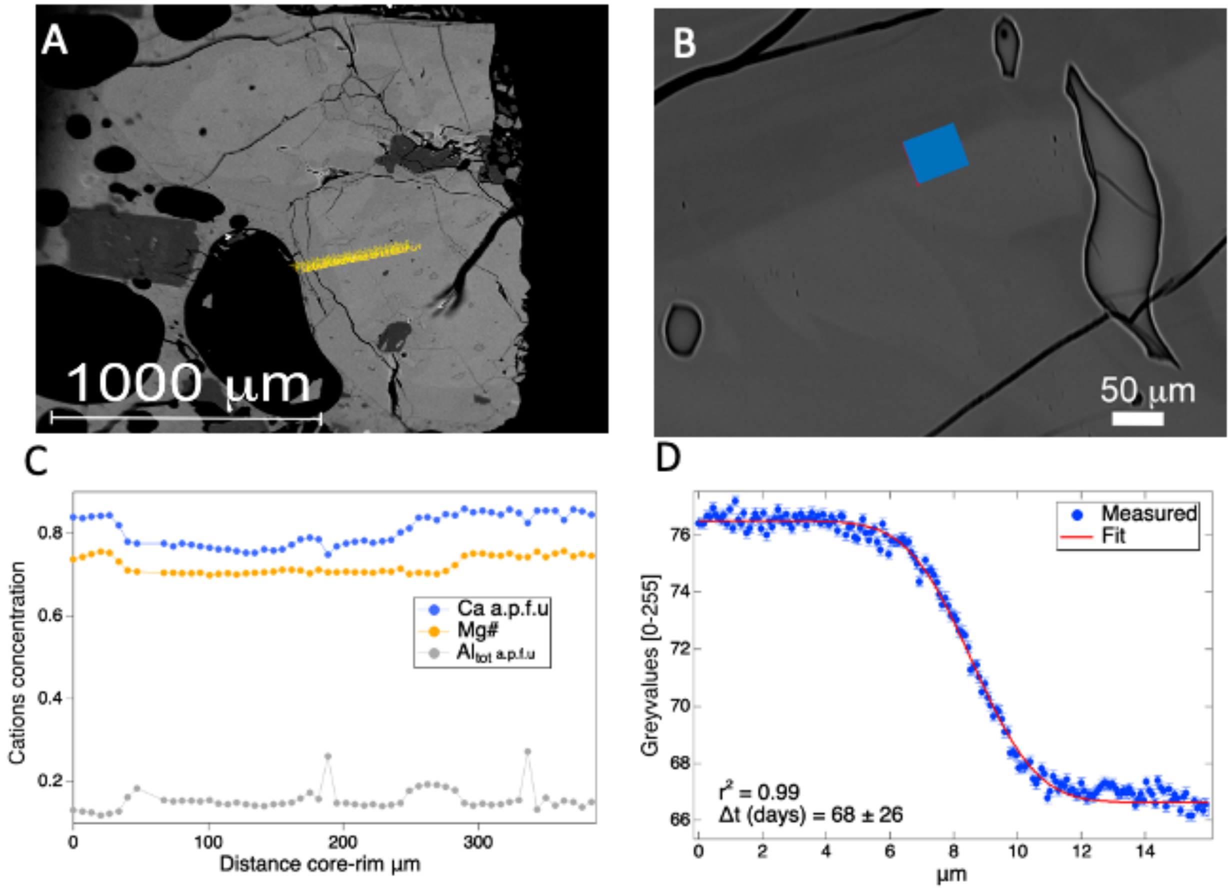

Supplementary Fig. 9.
Clinopyroxene chemical profile and diffusion modeling. (A) SEM-BSE image of the analyzed clinopyroxene. The yellow line with numbers marks the analyzed chemical profile reported in C. (B) SEM-BSE high resolution image showing the area (light blue area) where the grey scale profile for the diffusion profile has been extracted using the *greyvalues* Matlab script of the NIDIS model from Petrone *et al.*[5]. The red dot on the light blue area marks the initial point of the profile. (C) Variation of Ca (blue dots +line), Al (grey dots + line) (a.p.f.u.) and Mg# (orange dots + line) *vs* distance (µm) along the chemical profile shown in (A). (D) Grey values diffusion profile (blue dots with error bar) *vs* distance (µm) extracted along the light blue area in (B). The red line is the fit of the diffusion modeling calculated using the *createfit* Matlab script of the NIDIS model from Petrone *et al.*[5]. The goodness of the fitting is reported as r^2 and Δt is the calculated timescales (in days or years). The grey values are used as proxy of Mg# in clinopyroxene following Petrone *et al.*[5]. The temperature at which the diffusion has been calculated is reported in Table 1 for each clinopyroxene. See Method and Materials for further details

Paroxysm 3 July 2019, Sample P30-09, hp – Px9b – Subgroup 1A

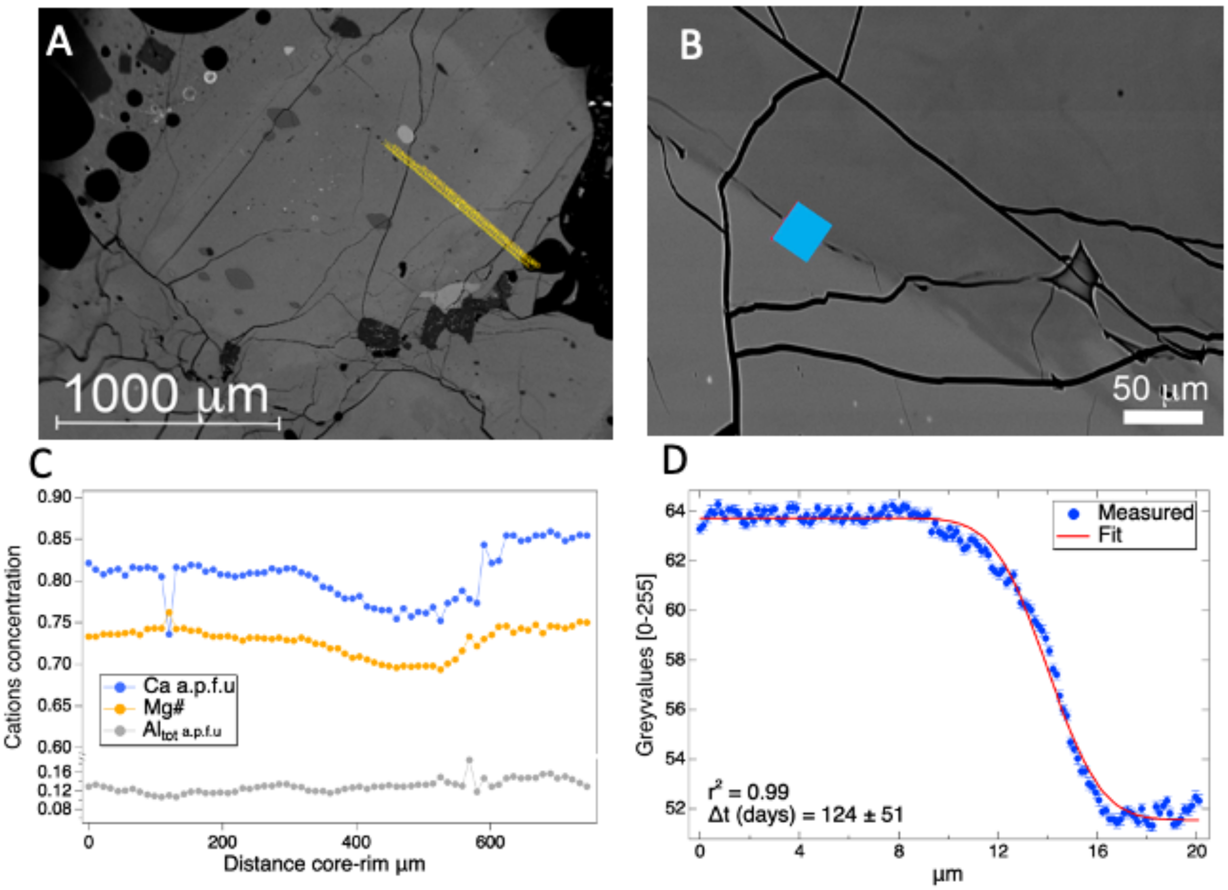

Supplementary Fig. 10.
Clinopyroxene chemical profile and diffusion modeling. (A) SEM-BSE image of the analyzed clinopyroxene. The yellow line with numbers marks the analyzed chemical profile reported in C. (B) SEM-BSE high resolution image showing the area (light blue area) where the grey scale profile for the diffusion profile has been extracted using the *greyvalues* Matlab script of the NIDIS model from Petrone *et al.*[5]. The red dot on the light blue area marks the initial point of the profile. (C) Variation of Ca (blue dots +line), Al (grey dots + line) (a.p.f.u.) and Mg# (orange dots + line) *vs* distance (µm) along the chemical profile shown in (A). (D) Grey values diffusion profile (blue dots with error bar) *vs* distance (µm) extracted along the light blue area in (B). The red line is the fit of the diffusion modeling calculated using the *createfit* Matlab script of the NIDIS model from Petrone *et al.*[5]. The goodness of the fitting is reported as r^2 and Δt is the calculated timescales (in days or years). The grey values are used as proxy of Mg# in clinopyroxene following Petrone *et al.*[5]. The temperature at which the diffusion has been calculated is reported in Table 1 for each clinopyroxene. See Method and Materials for further details

Paroxysm 3 July 2019, Sample P30-09, hp – Px13a – Subgroup 1A

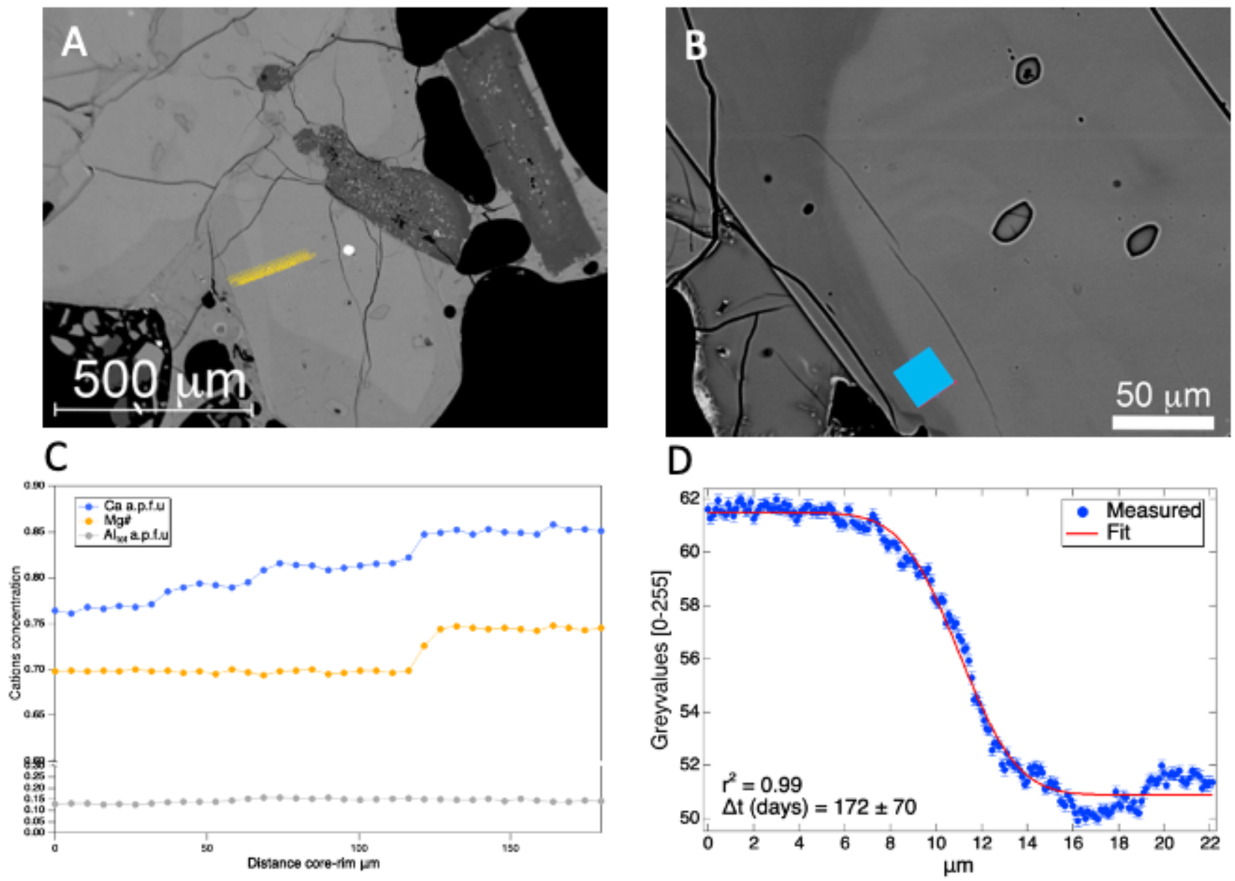

Supplementary Fig. 11.
Clinopyroxene chemical profile and diffusion modeling. (A) SEM-BSE image of the analyzed clinopyroxene. The yellow line with numbers marks the analyzed chemical profile reported in C. (B) SEM-BSE high resolution image showing the area (light blue area) where the grey scale profile for the diffusion profile has been extracted using the *greyvalues* Matlab script of the NIDIS model from Petrone *et al.*[5]. The red dot on the light blue area marks the initial point of the profile. (C) Variation of Ca (blue dots +line), Al (grey dots + line) (a.p.f.u.) and Mg# (orange dots + line) *vs* distance (µm) along the chemical profile shown in (A). (D) Grey values diffusion profile (blue dots with error bar) *vs* distance (µm) extracted along the light blue area in (B). The red line is the fit of the diffusion modeling calculated using the *createfit* Matlab script of the NIDIS model from Petrone *et al.*[5]. The goodness of the fitting is reported as r^2 and Δt is the calculated timescales (in days or years). The grey values are used as proxy of Mg# in clinopyroxene following Petrone *et al.*[5]. The temperature at which the diffusion has been calculated is reported in Table 1 for each clinopyroxene. See Method and Materials for further details

Paroxysm 28 August 2019, Sample P47-1, hp – Px11 – Subgroup 1A

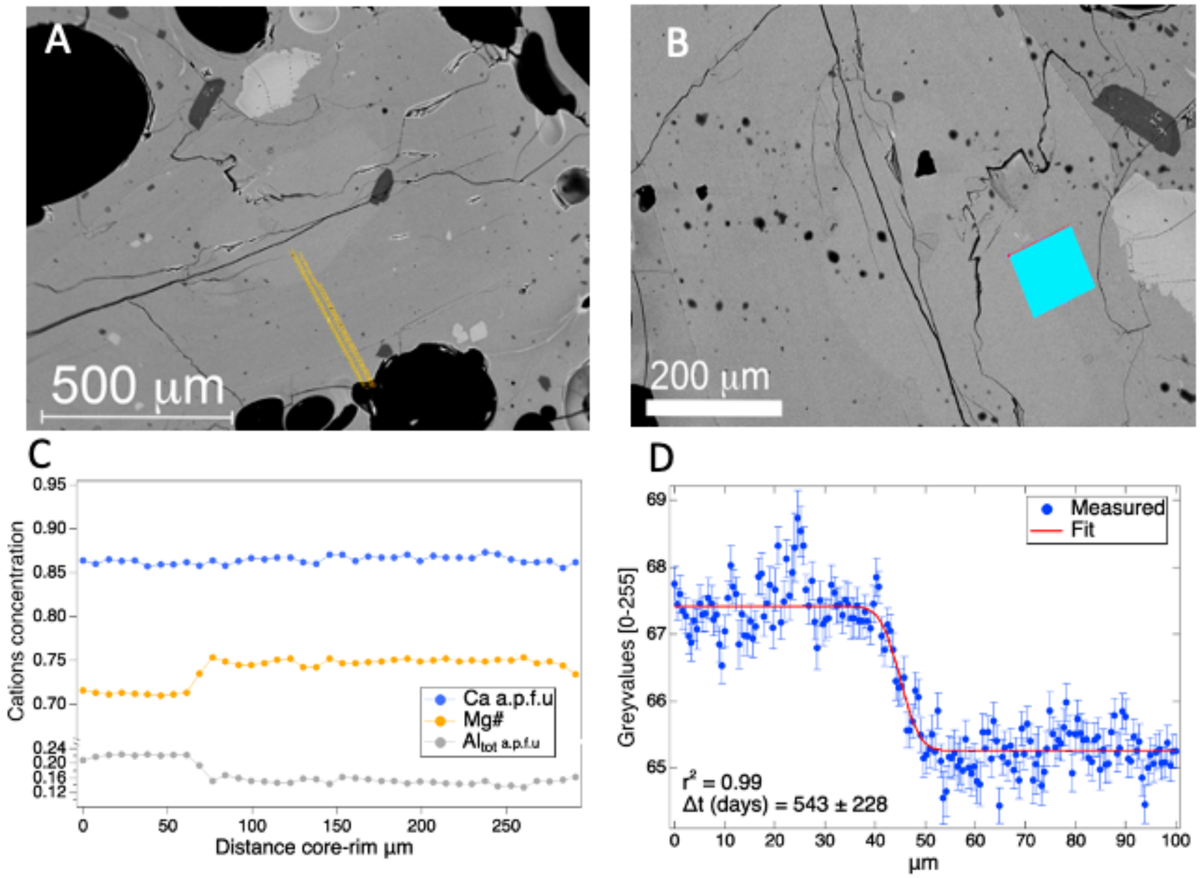

Supplementary Fig. 12.

Clinopyroxene chemical profile and diffusion modeling. (A) SEM-BSE image of the analyzed clinopyroxene. The yellow line with numbers marks the analyzed chemical profile reported in C. (B) SEM-BSE high resolution image showing the area (light blue area) where the grey scale profile for the diffusion profile has been extracted using the *greyvalues* Matlab script of the NIDIS model from Petrone *et al.*[5]. The red dot on the light blue area marks the initial point of the profile. (C) Variation of Ca (blue dots +line), Al (grey dots + line) (a.p.f.u.) and Mg# (orange dots + line) *vs* distance (µm) along the chemical profile shown in (A). (D) Grey values diffusion profile (blue dots with error bar) *vs* distance (µm) extracted along the light blue area in (B). The red line is the fit of the diffusion modeling calculated using the *createfit* Matlab script of the NIDIS model from Petrone *et al.*[5]. The goodness of the fitting is reported as r^2 and Δt is the calculated timescales (in days or years). The grey values are used as proxy of Mg# in clinopyroxene following Petrone *et al.*[5]. The temperature at which the diffusion has been calculated is reported in Table 1 for each clinopyroxene. See Method and Materials for further details

Paroxysm 28 August 2019, Sample P44-1, hp – Px3 – Subgroup 1A

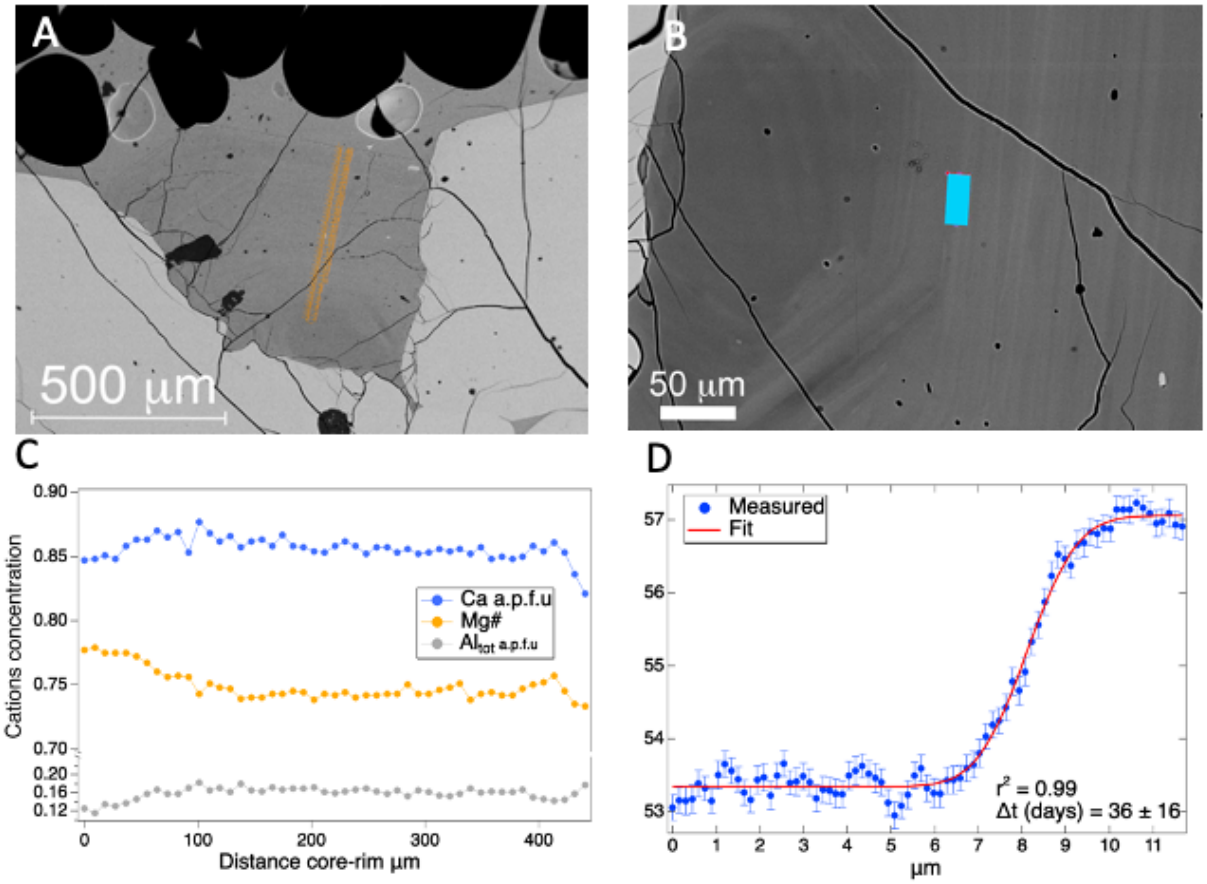

Supplementary Fig. 13.
Clinopyroxene chemical profile and diffusion modeling. (A) SEM-BSE image of the analyzed clinopyroxene. The yellow line with numbers marks the analyzed chemical profile reported in C. (B) SEM-BSE high resolution image showing the area (light blue area) where the grey scale profile for the diffusion profile has been extracted using the *greyvalues* Matlab script of the NIDIS model from Petrone *et al.*[5]. The red dot on the light blue area marks the initial point of the profile. (C) Variation of Ca (blue dots +line), Al (grey dots + line) (a.p.f.u.) and Mg# (orange dots + line) *vs* distance (µm) along the chemical profile shown in (A). (D) Grey values diffusion profile (blue dots with error bar) *vs* distance (µm) extracted along the light blue area in (B). The red line is the fit of the diffusion modeling calculated using the *createfit* Matlab script of the NIDIS model from Petrone *et al.*[5]. The goodness of the fitting is reported as r^2 and Δt is the calculated timescales (in days or years). The grey values are used as proxy of Mg# in clinopyroxene following Petrone *et al.*[5]. The temperature at which the diffusion has been calculated is reported in Table 1 for each clinopyroxene. See Method and Materials for further details

Paroxysm 28 August 2019, Sample P44-1, lp-hp – Px3 – Subgroup 1A

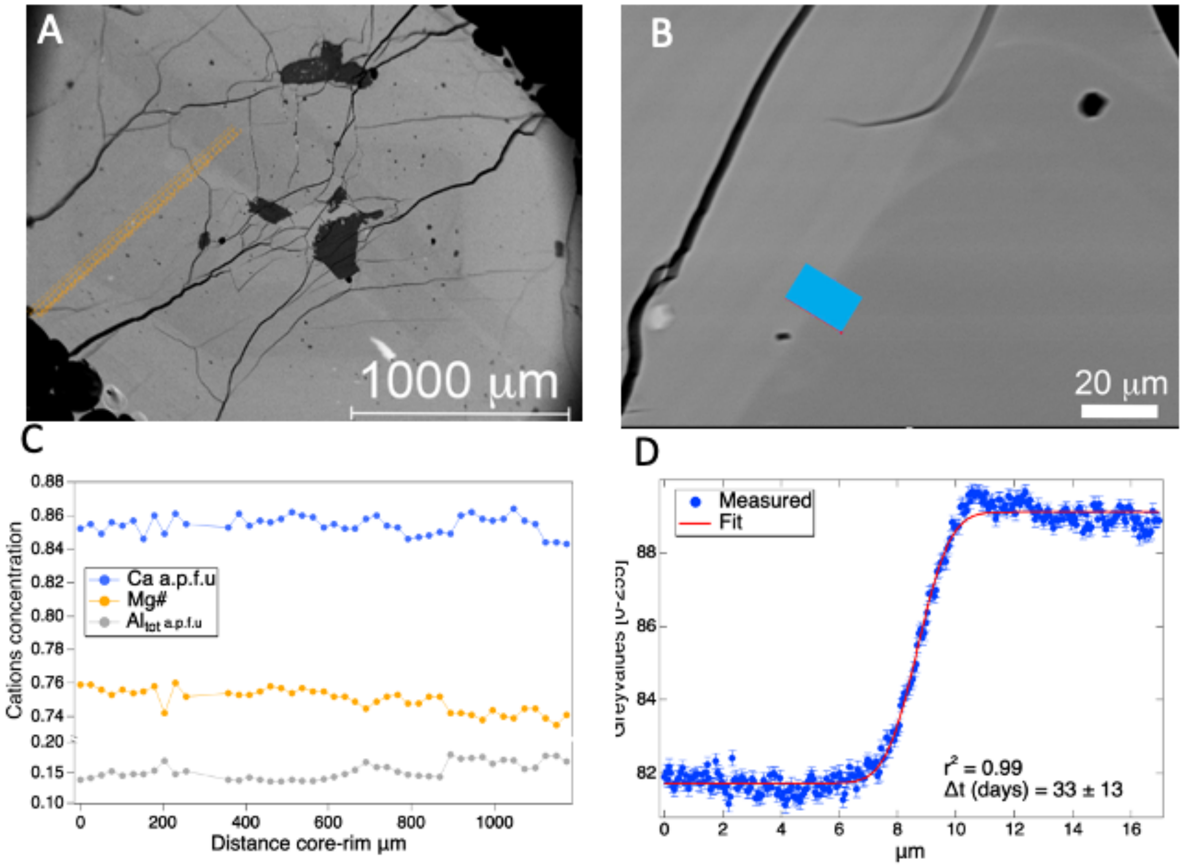

Supplementary Fig. 14.
Clinopyroxene chemical profile and diffusion modeling. (A) SEM-BSE image of the analyzed clinopyroxene. The yellow line with numbers marks the analyzed chemical profile reported in C. (B) SEM-BSE high resolution image showing the area (light blue area) where the grey scale profile for the diffusion profile has been extracted using the *greyvalues* Matlab script of the NIDIS model from Petrone *et al.*[5]. The red dot on the light blue area marks the initial point of the profile. (C) Variation of Ca (blue dots +line), Al (grey dots + line) (a.p.f.u.) and Mg# (orange dots + line) *vs* distance (μm) along the chemical profile shown in (A). (D) Grey values diffusion profile (blue dots with error bar) *vs* distance (μm) extracted along the light blue area in (B). The red line is the fit of the diffusion modeling calculated using the *createfit* Matlab script of the NIDIS model from Petrone *et al.*[5]. The goodness of the fitting is reported as r^2 and Δt is the calculated timescales (in days or years). The grey values are used as proxy of Mg# in clinopyroxene following Petrone *et al.*[5]. The temperature at which the diffusion has been calculated is reported in Table 1 for each clinopyroxene. See Method and Materials for further details

Paroxysm 3 July 2019, Sample P30-07, lp – Px6 – Subgroup 1B

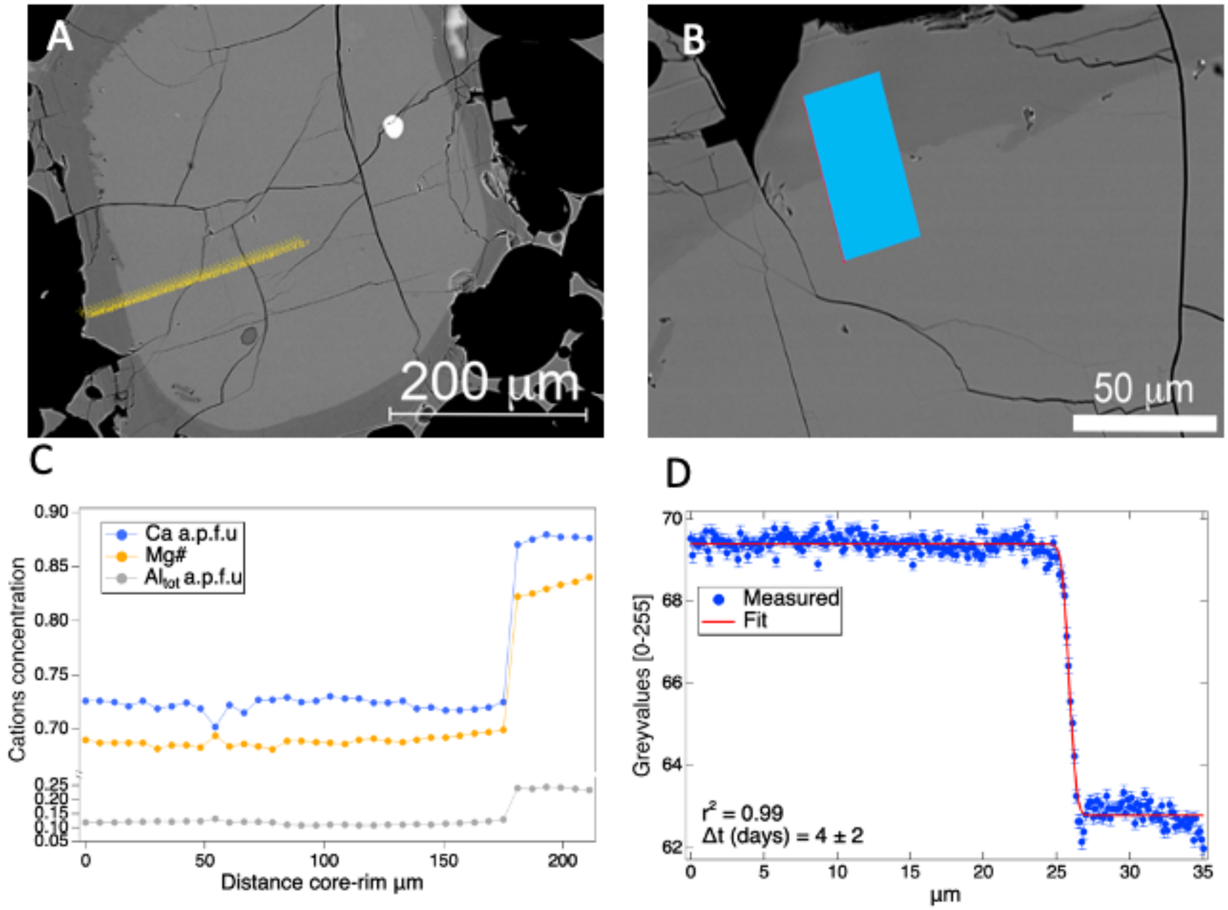

Supplementary Fig. 15.
Clinopyroxene chemical profile and diffusion modeling. (A) SEM-BSE image of the analyzed clinopyroxene. The yellow line with numbers marks the analyzed chemical profile reported in C. (B) SEM-BSE high resolution image showing the area (light blue area) where the grey scale profile for the diffusion profile has been extracted using the *greyvalues* Matlab script of the NIDIS model from Petrone *et al.*[5]. The red dot on the light blue area marks the initial point of the profile. (C) Variation of Ca (blue dots +line), Al (grey dots + line) (a.p.f.u.) and Mg# (orange dots + line) *vs* distance (µm) along the chemical profile shown in (A). (D) Grey values diffusion profile (blue dots with error bar) *vs* distance (µm) extracted along the light blue area in (B). The red line is the fit of the diffusion modeling calculated using the *createfit* Matlab script of the NIDIS model from Petrone *et al.*[5]. The goodness of the fitting is reported as r^2 and Δt is the calculated timescales (in days or years). The grey values are used as proxy of Mg# in clinopyroxene following Petrone *et al.*[5]. The temperature at which the diffusion has been calculated is reported in Table 1 for each clinopyroxene. See Method and Materials for further details

Paroxysm 3 July 2019, Sample P30-07, lp – Px9 – Subgroup 1B

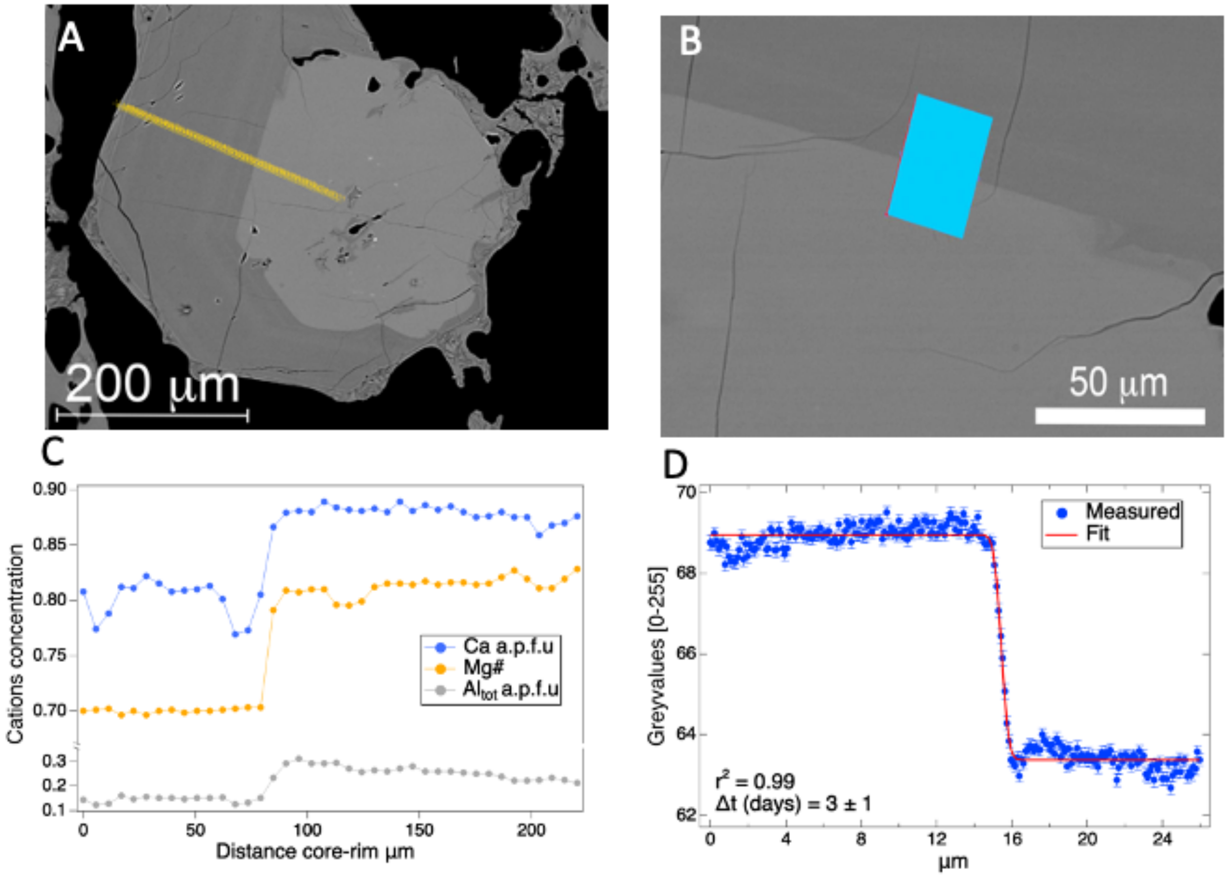

Supplementary Fig. 16.
Clinopyroxene chemical profile and diffusion modeling. (A) SEM-BSE image of the analyzed clinopyroxene. The yellow line with numbers marks the analyzed chemical profile reported in C. (B) SEM-BSE high resolution image showing the area (light blue area) where the grey scale profile for the diffusion profile has been extracted using the *greyvalues* Matlab script of the NIDIS model from Petrone *et al.*[5]. The red dot on the light blue area marks the initial point of the profile. (C) Variation of Ca (blue dots +line), Al (grey dots + line) (a.p.f.u.) and Mg# (orange dots + line) *vs* distance (μm) along the chemical profile shown in (A). (D) Grey values diffusion profile (blue dots with error bar) *vs* distance (μm) extracted along the light blue area in (B). The red line is the fit of the diffusion modeling calculated using the *createfit* Matlab script of the NIDIS model from Petrone *et al.*[5]. The goodness of the fitting is reported as r^2 and Δt is the calculated timescales (in days or years). The grey values are used as proxy of Mg# in clinopyroxene following Petrone *et al.*[5]. The temperature at which the diffusion has been calculated is reported in Table 1 for each clinopyroxene. See Method and Materials for further details

Paroxysm 3 July 2019, Sample P30-07, lp – Px11 – Subgroup 1B

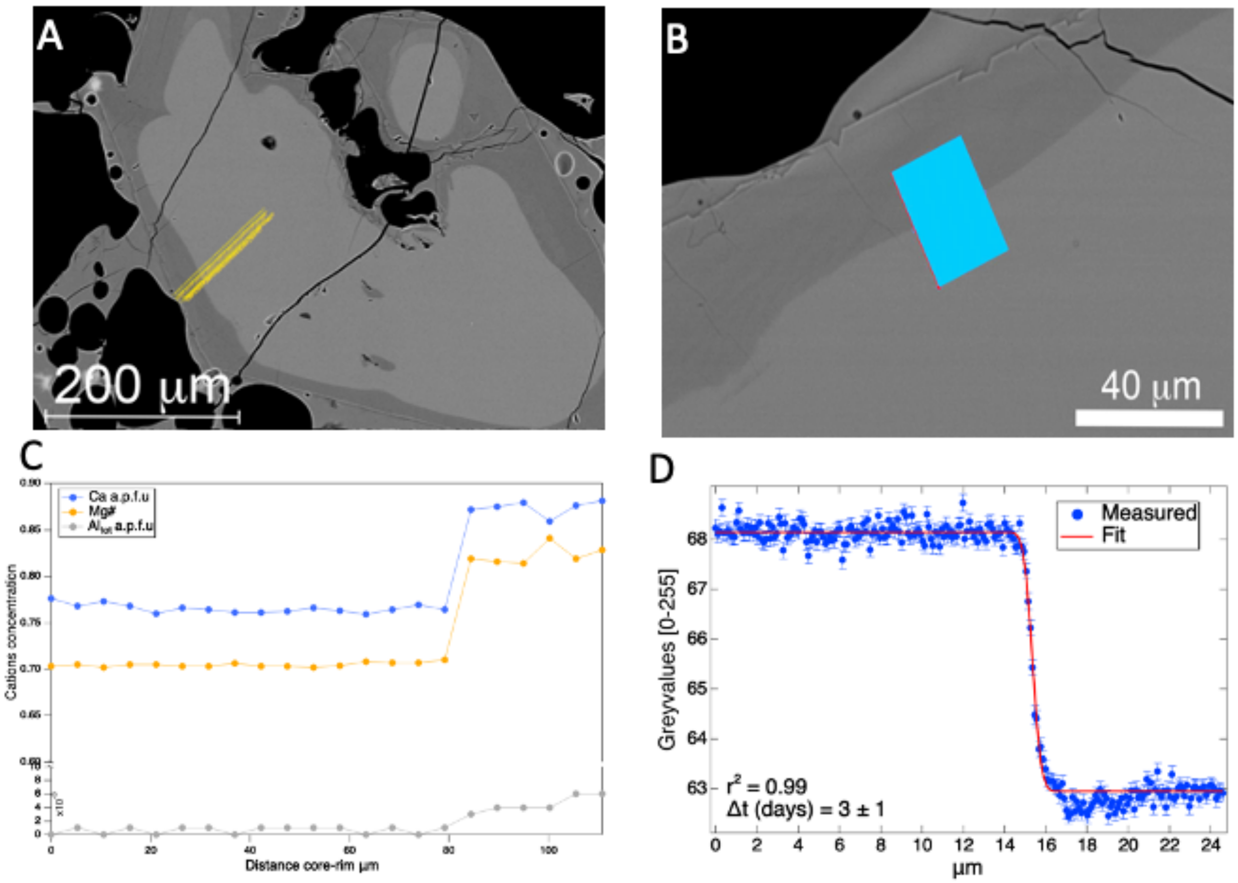

Supplementary Fig. 17.
Clinopyroxene chemical profile and diffusion modeling. (A) SEM-BSE image of the analyzed clinopyroxene. The yellow line with numbers marks the analyzed chemical profile reported in C. (B) SEM-BSE high resolution image showing the area (light blue area) where the grey scale profile for the diffusion profile has been extracted using the *greyvalues* Matlab script of the NIDIS model from Petrone *et al.*[5]. The red dot on the light blue area marks the initial point of the profile. (C) Variation of Ca (blue dots +line), Al (grey dots + line) (a.p.f.u.) and Mg# (orange dots + line) *vs* distance (µm) along the chemical profile shown in (A). (D) Grey values diffusion profile (blue dots with error bar) *vs* distance (µm) extracted along the light blue area in (B). The red line is the fit of the diffusion modeling calculated using the *createfit* Matlab script of the NIDIS model from Petrone *et al.*[5]. The goodness of the fitting is reported as r^2 and Δt is the calculated timescales (in days or years). The grey values are used as proxy of Mg# in clinopyroxene following Petrone *et al.*[5]. The temperature at which the diffusion has been calculated is reported in Table 1 for each clinopyroxene. See Method and Materials for further details

Paroxysm 3 July 2019, Sample P10-05, lp – Px4 – Subgroup 1B

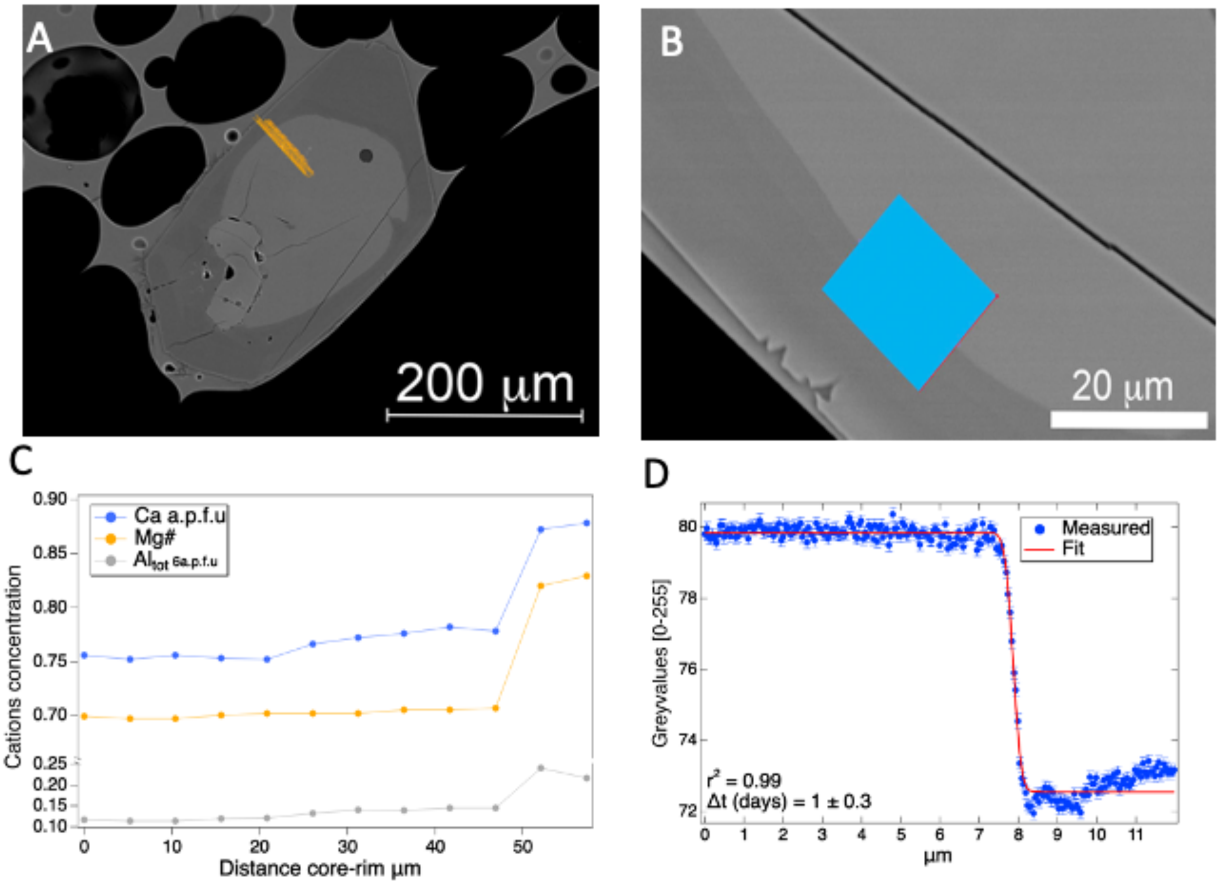

Supplementary Fig. 18.

Clinopyroxene chemical profile and diffusion modeling. (A) SEM-BSE image of the analyzed clinopyroxene. The yellow line with numbers marks the analyzed chemical profile reported in C. (B) SEM-BSE high resolution image showing the area (light blue area) where the grey scale profile for the diffusion profile has been extracted using the *greyvalues* Matlab script of the NIDIS model from Petrone *et al.*[5]. The red dot on the light blue area marks the initial point of the profile. (C) Variation of Ca (blue dots +line), Al (grey dots + line) (a.p.f.u.) and Mg# (orange dots + line) *vs* distance (µm) along the chemical profile shown in (A). (D) Grey values diffusion profile (blue dots with error bar) *vs* distance (µm) extracted along the light blue area in (B). The red line is the fit of the diffusion modeling calculated using the *createfit* Matlab script of the NIDIS model from Petrone *et al.*[5]. The goodness of the fitting is reported as r^2 and Δt is the calculated timescales (in days or years). The grey values are used as proxy of Mg# in clinopyroxene following Petrone *et al.*[5]. The temperature at which the diffusion has been calculated is reported in Table 1 for each clinopyroxene. See Method and Materials for further details

Paroxysm 3 July 2019, Sample P01-05, lp – Px4 – Subgroup 1B

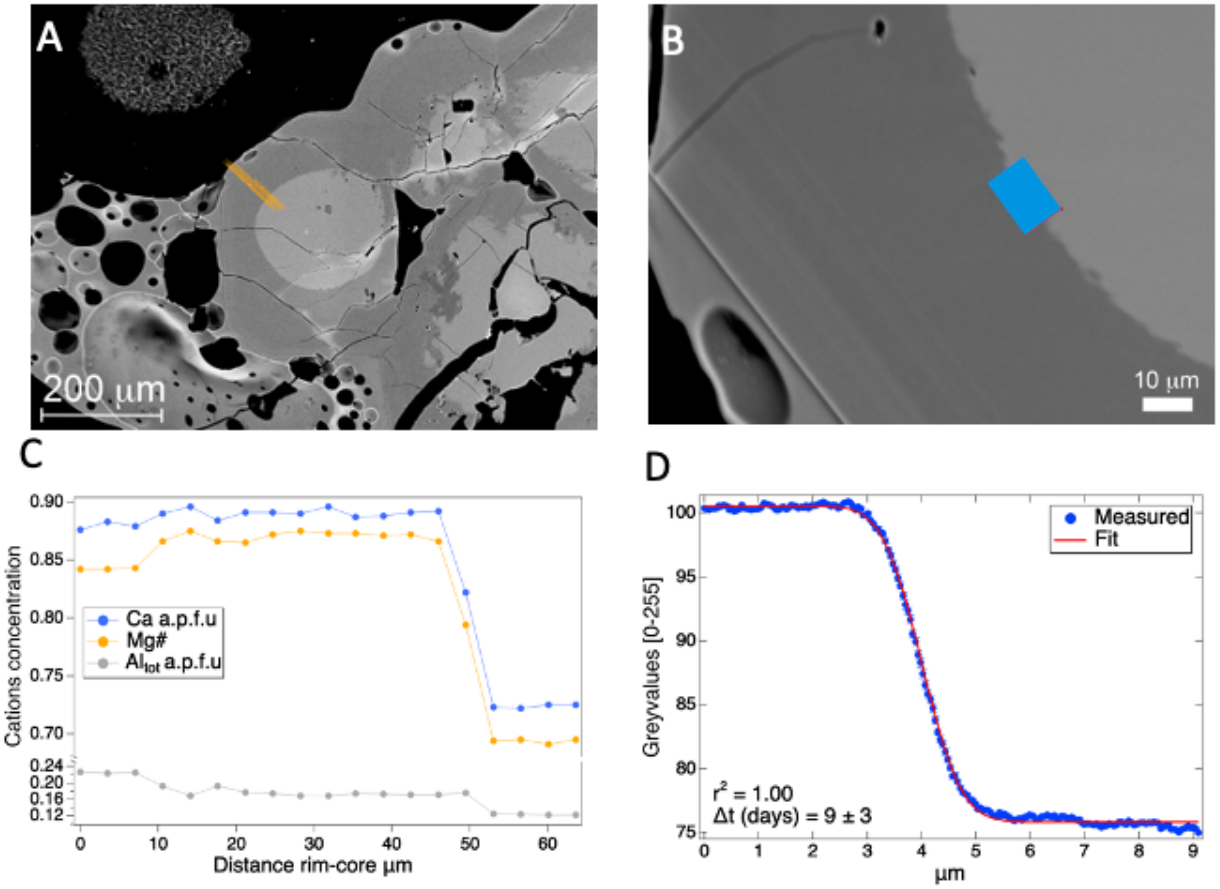

Supplementary Fig. 19.
Clinopyroxene chemical profile and diffusion modeling. (A) SEM-BSE image of the analyzed clinopyroxene. The yellow line with numbers marks the analyzed chemical profile reported in C. (B) SEM-BSE high resolution image showing the area (light blue area) where the grey scale profile for the diffusion profile has been extracted using the *greyvalues* Matlab script of the NIDIS model from Petrone *et al.*[5]. The red dot on the light blue area marks the initial point of the profile. (C) Variation of Ca (blue dots +line), Al (grey dots + line) (a.p.f.u.) and Mg# (orange dots + line) *vs* distance (µm) along the chemical profile shown in (A). (D) Grey values diffusion profile (blue dots with error bar) *vs* distance (µm) extracted along the light blue area in (B). The red line is the fit of the diffusion modeling calculated using the *createfit* Matlab script of the NIDIS model from Petrone *et al.*[5]. The goodness of the fitting is reported as r^2 and Δt is the calculated timescales (in days or years). The grey values are used as proxy of Mg# in clinopyroxene following Petrone *et al.*[5]. The temperature at which the diffusion has been calculated is reported in Table 1 for each clinopyroxene. See Method and Materials for further details

Paroxysm 3 July 2019, Sample P01-05, lp – Px6 – Subgroup 1B

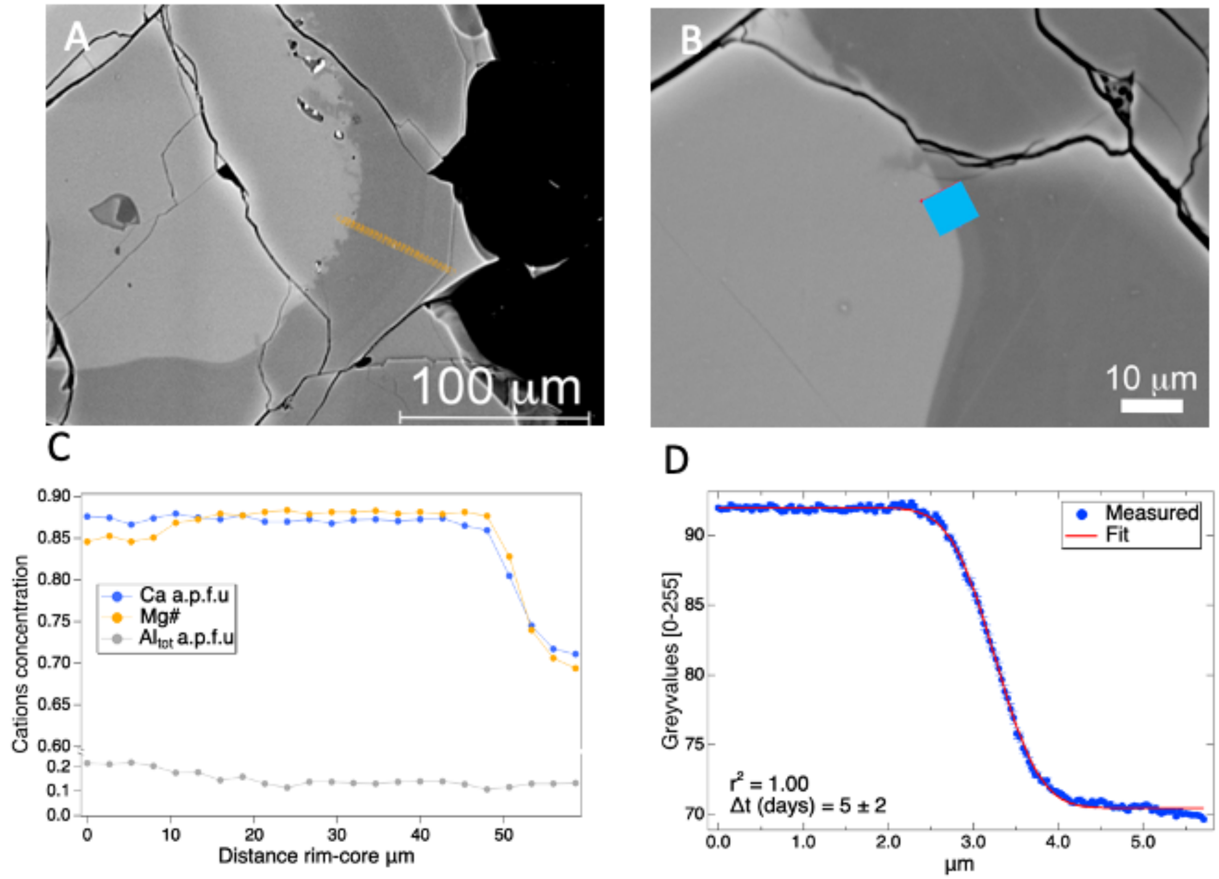

Supplementary Fig. 20.
Clinopyroxene chemical profile and diffusion modeling. (A) SEM-BSE image of the analyzed clinopyroxene. The yellow line with numbers marks the analyzed chemical profile reported in C. (B) SEM-BSE high resolution image showing the area (light blue area) where the grey scale profile for the diffusion profile has been extracted using the *greyvalues* Matlab script of the NIDIS model from Petrone *et al.*[5]. The red dot on the light blue area marks the initial point of the profile. (C) Variation of Ca (blue dots +line), Al (grey dots + line) (a.p.f.u.) and Mg# (orange dots + line) *vs* distance (µm) along the chemical profile shown in (A). (D) Grey values diffusion profile (blue dots with error bar) *vs* distance (µm) extracted along the light blue area in (B). The red line is the fit of the diffusion modeling calculated using the *createfit* Matlab script of the NIDIS model from Petrone *et al.*[5]. The goodness of the fitting is reported as r^2 and Δt is the calculated timescales (in days or years). The grey values are used as proxy of Mg# in clinopyroxene following Petrone *et al.*[5]. The temperature at which the diffusion has been calculated is reported in Table 1 for each clinopyroxene. See Method and Materials for further details

Paroxysm 3 July 2019, Sample P01-05, lp – Px7 – Subgroup 1B

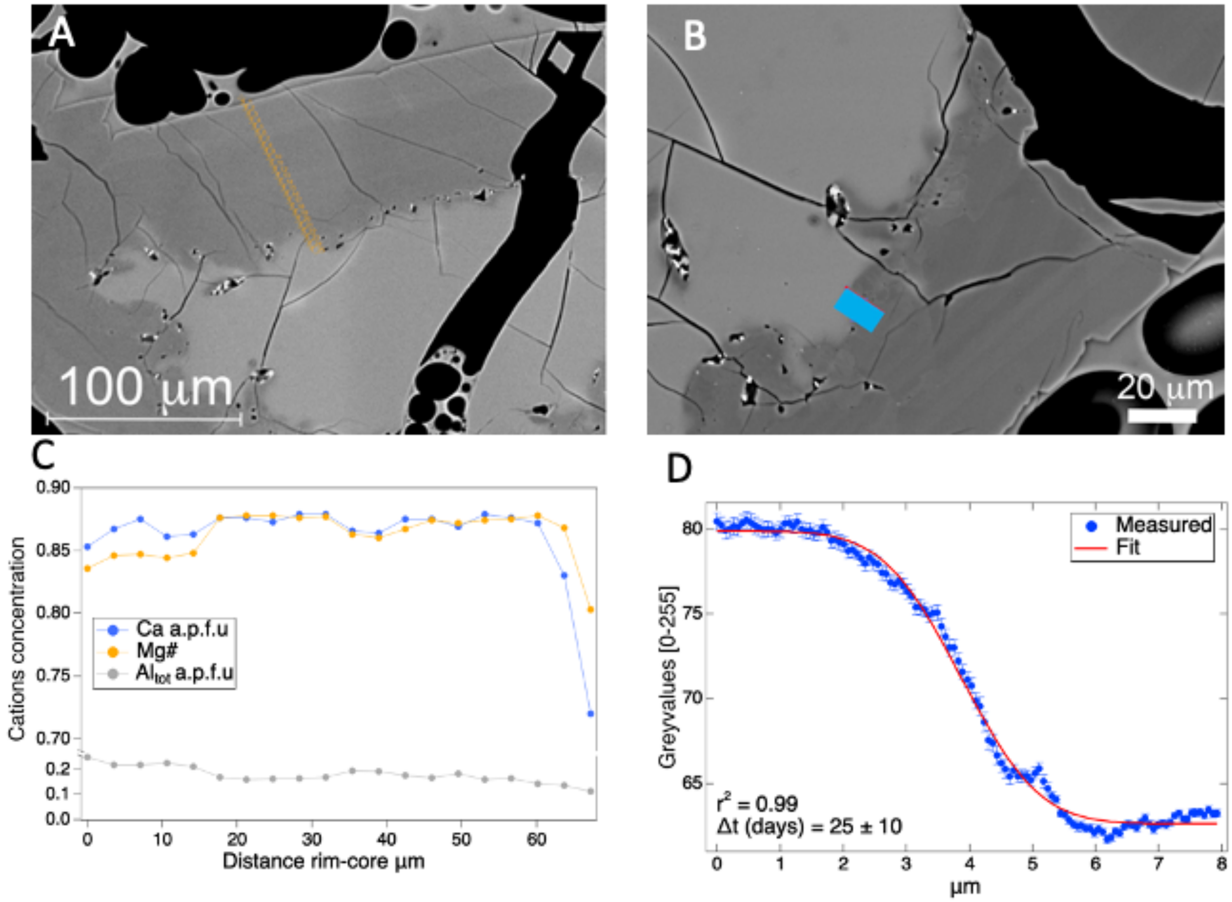

Supplementary Fig. 21.
Clinopyroxene chemical profile and diffusion modeling. (A) SEM-BSE image of the analyzed clinopyroxene. The yellow line with numbers marks the analyzed chemical profile reported in C. (B) SEM-BSE high resolution image showing the area (light blue area) where the grey scale profile for the diffusion profile has been extracted using the *greyvalues* Matlab script of the NIDIS model from Petrone *et al.*[5]. The red dot on the light blue area marks the initial point of the profile. (C) Variation of Ca (blue dots +line), Al (grey dots + line) (a.p.f.u.) and Mg# (orange dots + line) *vs* distance (µm) along the chemical profile shown in (A). (D) Grey values diffusion profile (blue dots with error bar) *vs* distance (µm) extracted along the light blue area in (B). The red line is the fit of the diffusion modeling calculated using the *createfit* Matlab script of the NIDIS model from Petrone *et al.*[5]. The goodness of the fitting is reported as r^2 and Δt is the calculated timescales (in days or years). The grey values are used as proxy of Mg# in clinopyroxene following Petrone *et al.*[5]. The temperature at which the diffusion has been calculated is reported in Table 1 for each clinopyroxene. See Method and Materials for further details

Paroxysm 3 July 2019, Sample P01-05, lp – Px11 – Subgroup 1B

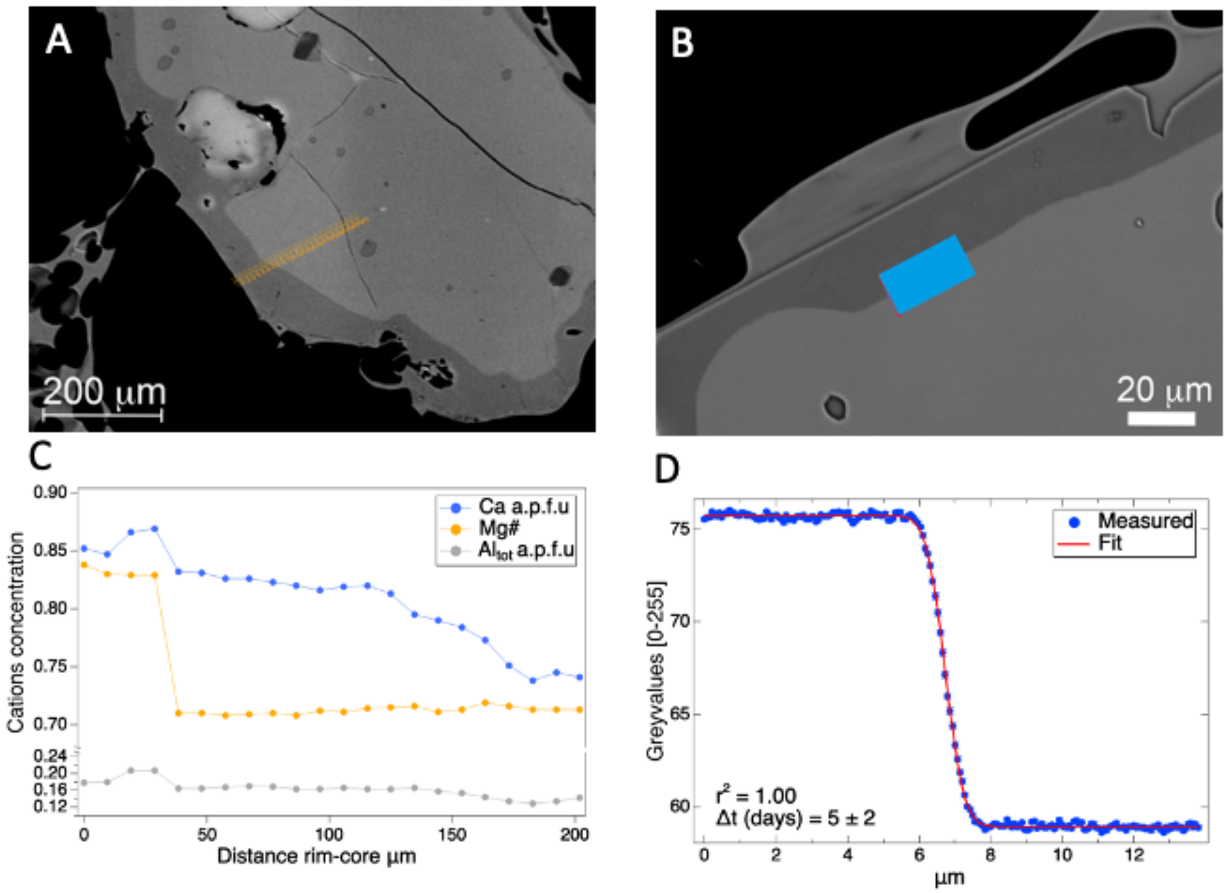

Supplementary Fig. 22.
Clinopyroxene chemical profile and diffusion modeling. (A) SEM-BSE image of the analyzed clinopyroxene. The yellow line with numbers marks the analyzed chemical profile reported in C. (B) SEM-BSE high resolution image showing the area (light blue area) where the grey scale profile for the diffusion profile has been extracted using the *greyvalues* Matlab script of the NIDIS model from Petrone *et al.*[5]. The red dot on the light blue area marks the initial point of the profile. (C) Variation of Ca (blue dots +line), Al (grey dots + line) (a.p.f.u.) and Mg# (orange dots + line) *vs* distance (μm) along the chemical profile shown in (A). (D) Grey values diffusion profile (blue dots with error bar) *vs* distance (μm) extracted along the light blue area in (B). The red line is the fit of the diffusion modeling calculated using the *createfit* Matlab script of the NIDIS model from Petrone *et al.*[5]. The goodness of the fitting is reported as r^2 and Δt is the calculated timescales (in days or years). The grey values are used as proxy of Mg# in clinopyroxene following Petrone *et al.*[5]. The temperature at which the diffusion has been calculated is reported in Table 1 for each clinopyroxene. See Method and Materials for further details

Paroxysm 3 July 2019, Sample P21-1, lp – Px3 – Subgroup 1B

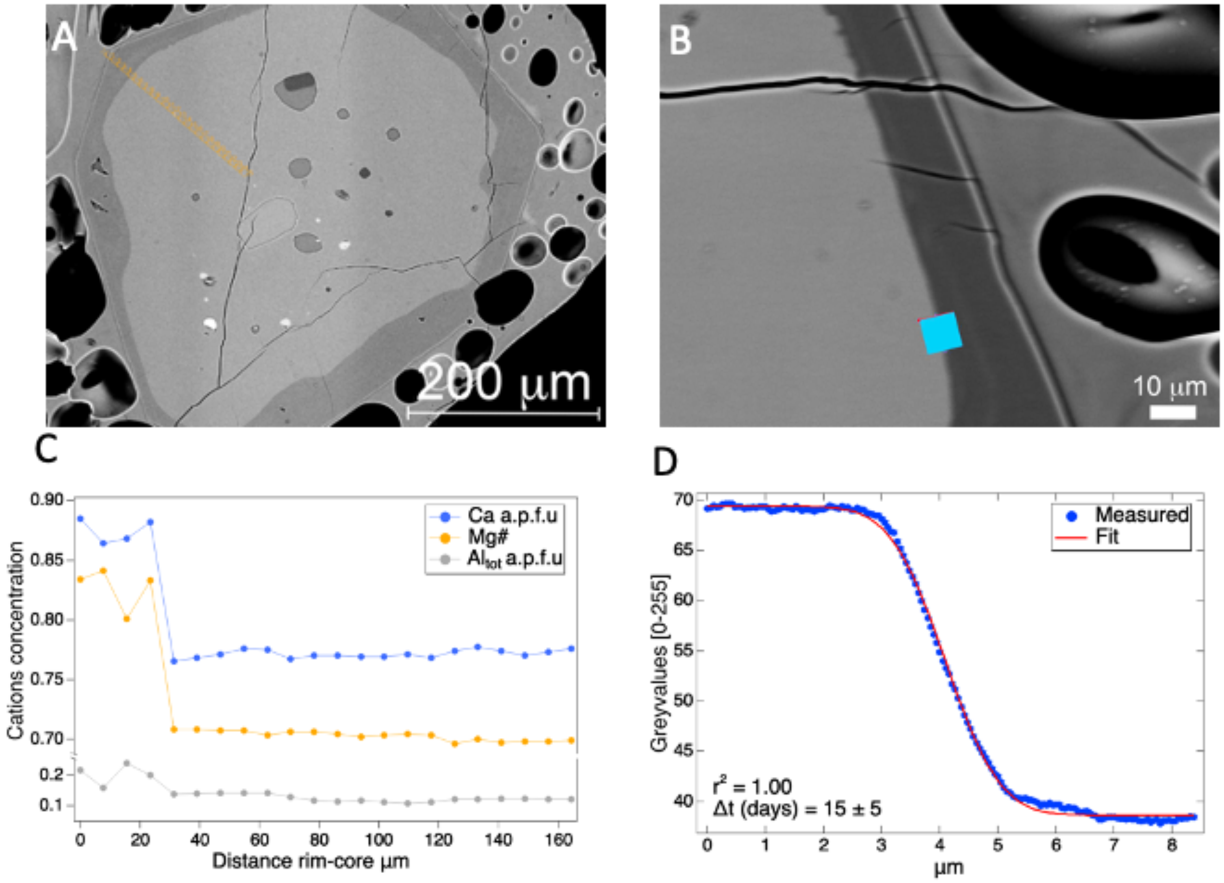

Supplementary Fig. 23.
Clinopyroxene chemical profile and diffusion modeling. (A) SEM-BSE image of the analyzed clinopyroxene. The yellow line with numbers marks the analyzed chemical profile reported in C. (B) SEM-BSE high resolution image showing the area (light blue area) where the grey scale profile for the diffusion profile has been extracted using the *greyvalues* Matlab script of the NIDIS model from Petrone *et al.*[5]. The red dot on the light blue area marks the initial point of the profile. (C) Variation of Ca (blue dots +line), Al (grey dots + line) (a.p.f.u.) and Mg# (orange dots + line) *vs* distance (µm) along the chemical profile shown in (A). (D) Grey values diffusion profile (blue dots with error bar) *vs* distance (µm) extracted along the light blue area in (B). The red line is the fit of the diffusion modeling calculated using the *createfit* Matlab script of the NIDIS model from Petrone *et al.*[5]. The goodness of the fitting is reported as r^2 and Δt is the calculated timescales (in days or years). The grey values are used as proxy of Mg# in clinopyroxene following Petrone *et al.*[5]. The temperature at which the diffusion has been calculated is reported in Table 1 for each clinopyroxene. See Method and Materials for further details

Paroxysm 3 July 2019, Sample P21-1, lp – Px7 – Subgroup 1B

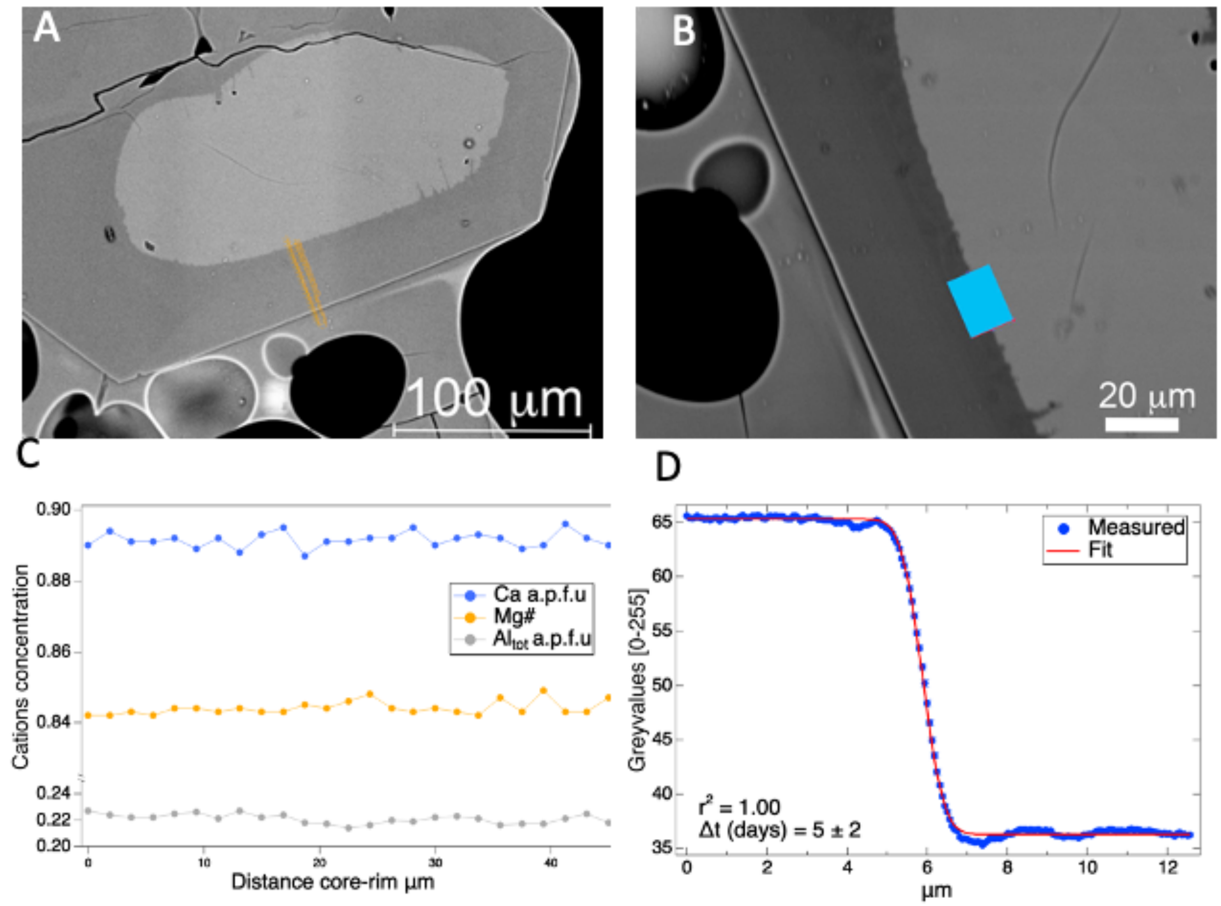

Supplementary Fig. 24.
Clinopyroxene chemical profile and diffusion modeling. (A) SEM-BSE image of the analyzed clinopyroxene. The yellow line with numbers marks the analyzed chemical profile reported in C. (B) SEM-BSE high resolution image showing the area (light blue area) where the grey scale profile for the diffusion profile has been extracted using the *greyvalues* Matlab script of the NIDIS model from Petrone *et al.*[5]. The red dot on the light blue area marks the initial point of the profile. (C) Variation of Ca (blue dots +line), Al (grey dots + line) (a.p.f.u.) and Mg# (orange dots + line) *vs* distance (µm) along the chemical profile shown in (A). (D) Grey values diffusion profile (blue dots with error bar) *vs* distance (µm) extracted along the light blue area in (B). The red line is the fit of the diffusion modeling calculated using the *createfit* Matlab script of the NIDIS model from Petrone *et al.*[5]. The goodness of the fitting is reported as r^2 and Δt is the calculated timescales (in days or years). The grey values are used as proxy of Mg# in clinopyroxene following Petrone *et al.*[5]. The temperature at which the diffusion has been calculated is reported in Table 1 for each clinopyroxene. See Method and Materials for further details

Paroxysm 3 July 2019, Sample P21-1, lp – Px8 – Subgroup 1B

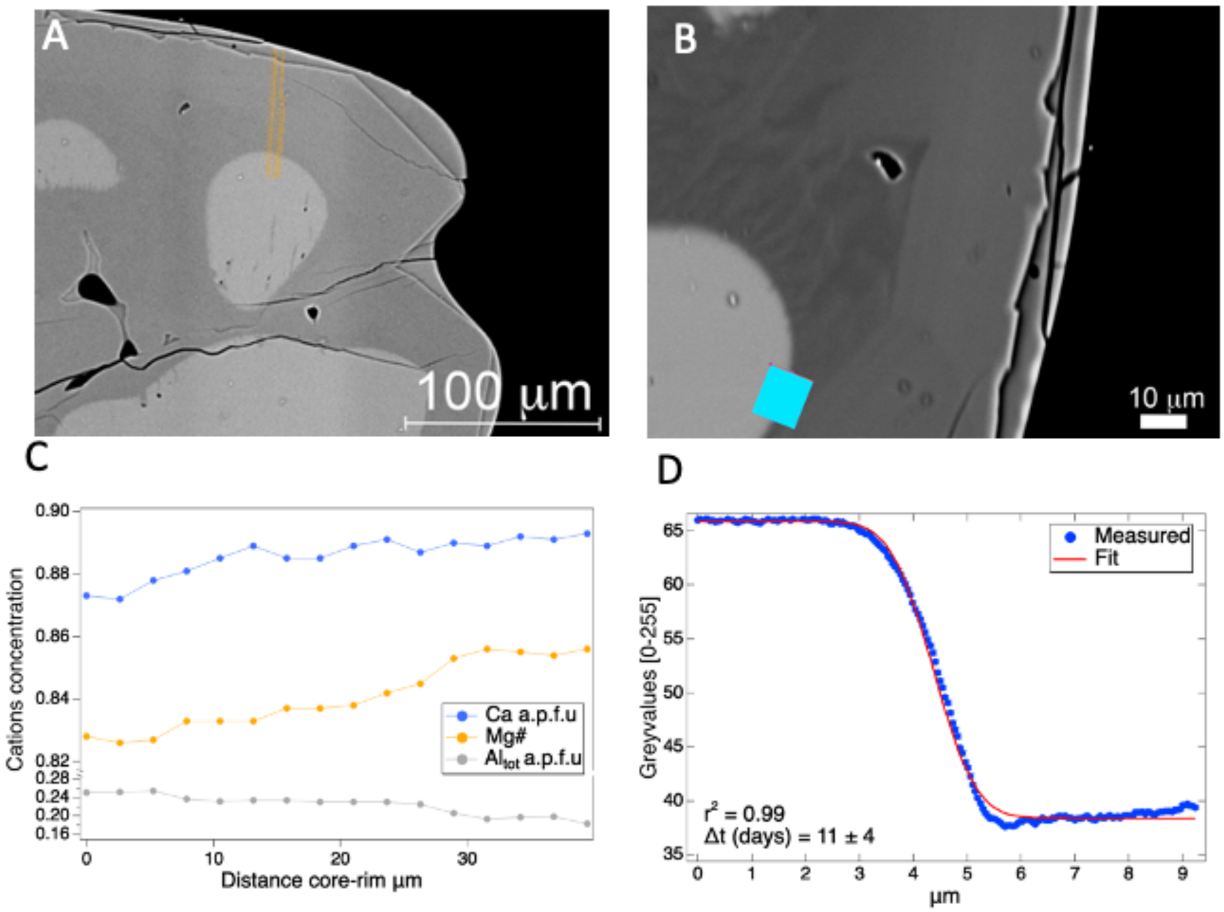

Supplementary Fig. 25.
Clinopyroxene chemical profile and diffusion modeling. (A) SEM-BSE image of the analyzed clinopyroxene. The yellow line with numbers marks the analyzed chemical profile reported in C. (B) SEM-BSE high resolution image showing the area (light blue area) where the grey scale profile for the diffusion profile has been extracted using the *greyvalues* Matlab script of the NIDIS model from Petrone *et al.*[5]. The red dot on the light blue area marks the initial point of the profile. (C) Variation of Ca (blue dots +line), Al (grey dots + line) (a.p.f.u.) and Mg# (orange dots + line) *vs* distance (µm) along the chemical profile shown in (A). (D) Grey values diffusion profile (blue dots with error bar) *vs* distance (µm) extracted along the light blue area in (B). The red line is the fit of the diffusion modeling calculated using the *createfit* Matlab script of the NIDIS model from Petrone *et al.*[5]. The goodness of the fitting is reported as r^2 and Δt is the calculated timescales (in days or years). The grey values are used as proxy of Mg# in clinopyroxene following Petrone *et al.*[5]. The temperature at which the diffusion has been calculated is reported in Table 1 for each clinopyroxene. See Method and Materials for further details

Paroxysm 3 July 2019, Sample P24-3, lp – Px3 – Subgroup 1B

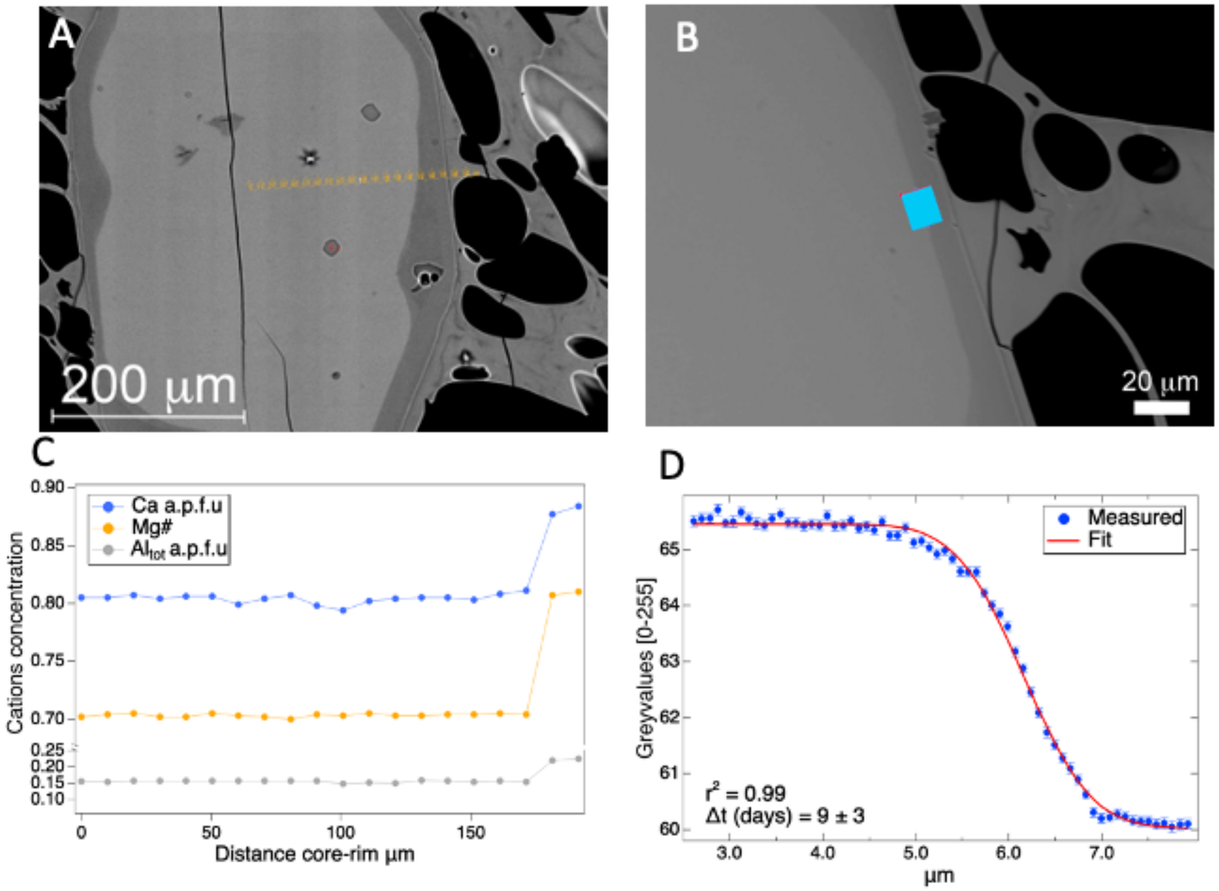

Supplementary Fig. 26.
Clinopyroxene chemical profile and diffusion modeling. (A) SEM-BSE image of the analyzed clinopyroxene. The yellow line with numbers marks the analyzed chemical profile reported in C. (B) SEM-BSE high resolution image showing the area (light blue area) where the grey scale profile for the diffusion profile has been extracted using the *greyvalues* Matlab script of the NIDIS model from Petrone *et al.*[5]. The red dot on the light blue area marks the initial point of the profile. (C) Variation of Ca (blue dots +line), Al (grey dots + line) (a.p.f.u.) and Mg# (orange dots + line) *vs* distance (µm) along the chemical profile shown in (A). (D) Grey values diffusion profile (blue dots with error bar) *vs* distance (µm) extracted along the light blue area in (B). The red line is the fit of the diffusion modeling calculated using the *createfit* Matlab script of the NIDIS model from Petrone *et al.*[5]. The goodness of the fitting is reported as r^2 and Δt is the calculated timescales (in days or years). The grey values are used as proxy of Mg# in clinopyroxene following Petrone *et al.*[5]. The temperature at which the diffusion has been calculated is reported in Table 1 for each clinopyroxene. See Method and Materials for further details

Paroxysm 3 July 2019, Sample P24-3, lp – Px4 – Subgroup 1B

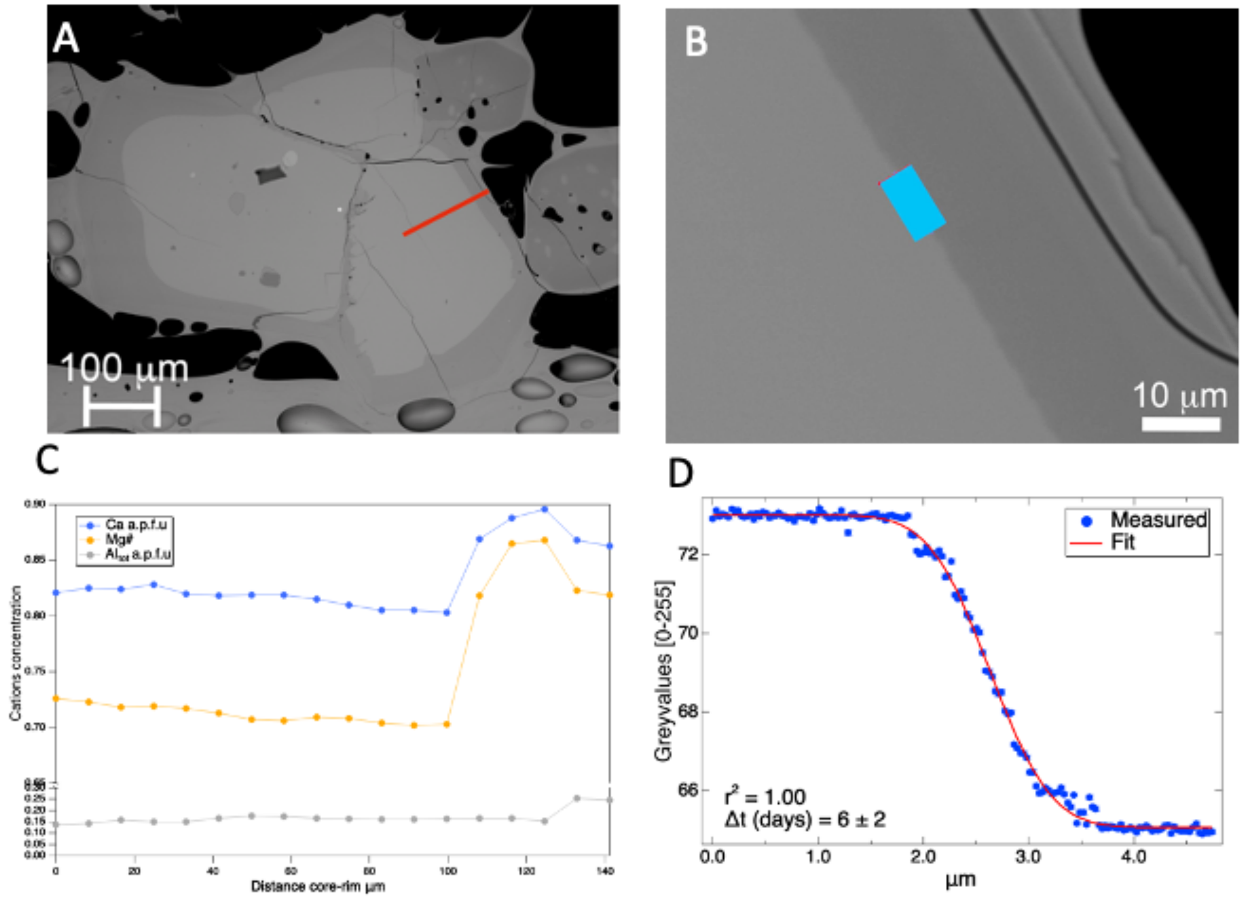

Supplementary Fig. 27.
Clinopyroxene chemical profile and diffusion modeling. (A) SEM-BSE image of the analyzed clinopyroxene. The red line marks the analyzed chemical profile reported in C. (B) SEM-BSE high resolution image showing the area (light blue area) where the grey scale profile for the diffusion profile has been extracted using the *greyvalues* Matlab script of the NIDIS model from Petrone *et al.*[5]. The red dot on the light blue area marks the initial point of the profile. (C) Variation of Ca (blue dots +line), Al (grey dots + line) (a.p.f.u.) and Mg# (orange dots + line) *vs* distance (µm) along the chemical profile shown in (A). (D) Grey values diffusion profile (blue dots with error bar) *vs* distance (µm) extracted along the light blue area in (B). The red line is the fit of the diffusion modeling calculated using the *createfit* Matlab script of the NIDIS model from Petrone *et al.*[5]. The goodness of the fitting is reported as r^2 and Δt is the calculated timescales (in days or years). The grey values are used as proxy of Mg# in clinopyroxene following Petrone *et al.*[5]. The temperature at which the diffusion has been calculated is reported in Table 1 for each clinopyroxene. See Method and Materials for further details.

Paroxysm 3 July 2019, Sample P24-3, lp – Px5 – Subgroup 1B

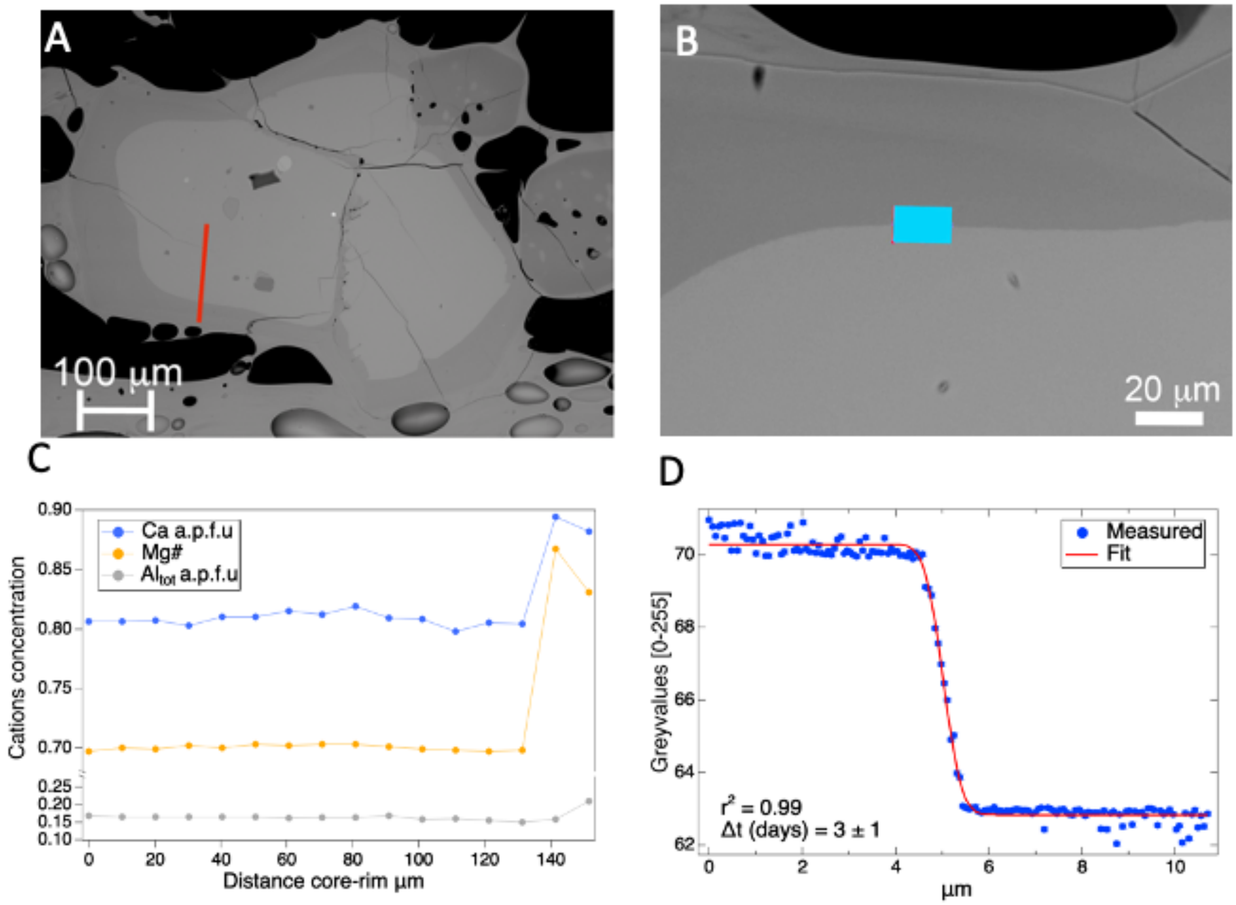

Supplementary Fig. 28.
Clinopyroxene chemical profile and diffusion modeling. (A) SEM-BSE image of the analyzed clinopyroxene. The red line marks the analyzed chemical profile reported in C. (B) SEM-BSE high resolution image showing the area (light blue area) where the grey scale profile for the diffusion profile has been extracted using the *greyvalues* Matlab script of the NIDIS model from Petrone *et al.*[5]. The red dot on the light blue area marks the initial point of the profile. (C) Variation of Ca (blue dots +line), Al (grey dots + line) (a.p.f.u.) and Mg# (orange dots + line) *vs* distance (µm) along the chemical profile shown in (A). (D) Grey values diffusion profile (blue dots with error bar) *vs* distance (µm) extracted along the light blue area in (B). The red line is the fit of the diffusion modeling calculated using the *createfit* Matlab script of the NIDIS model from Petrone *et al.*[5]. The goodness of the fitting is reported as r^2 and Δt is the calculated timescales (in days or years). The grey values are used as proxy of Mg# in clinopyroxene following Petrone *et al.*[5]. The temperature at which the diffusion has been calculated is reported in Table 1 for each clinopyroxene. See Method and Materials for further details.

Paroxysm 3 July 2019, Sample P24-3, lp – Px6 – Subgroup 1B

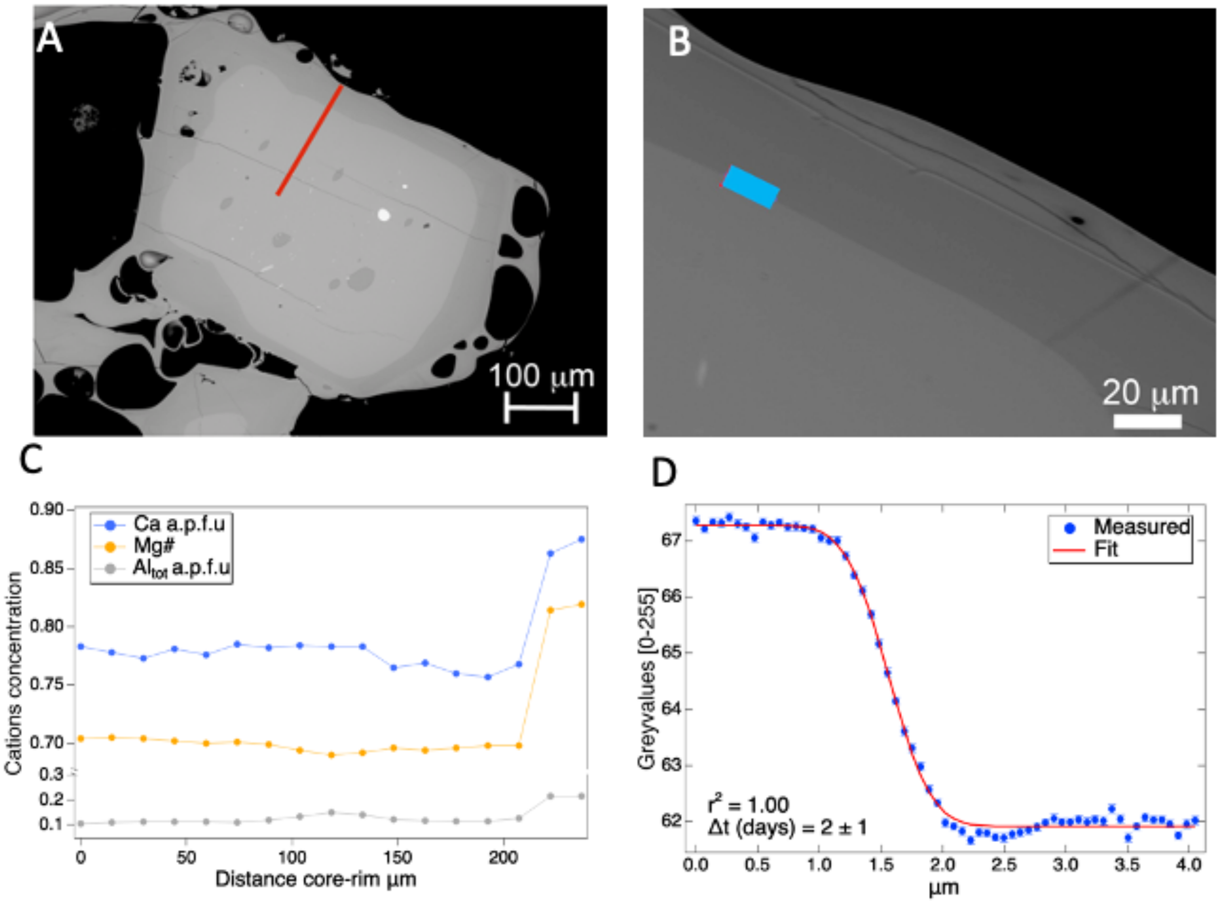

Supplementary Fig. 29.
Clinopyroxene chemical profile and diffusion modeling. (A) SEM-BSE image of the analyzed clinopyroxene. The red line marks the analyzed chemical profile reported in C. (B) SEM-BSE high resolution image showing the area (light blue area) where the grey scale profile for the diffusion profile has been extracted using the *greyvalues* Matlab script of the NIDIS model from Petrone *et al.*[5]. The red dot on the light blue area marks the initial point of the profile. (C) Variation of Ca (blue dots +line), Al (grey dots + line) (a.p.f.u.) and Mg# (orange dots + line) *vs* distance (µm) along the chemical profile shown in (A). (D) Grey values diffusion profile (blue dots with error bar) *vs* distance (µm) extracted along the light blue area in (B). The red line is the fit of the diffusion modeling calculated using the *createfit* Matlab script of the NIDIS model from Petrone *et al.*[5]. The goodness of the fitting is reported as r^2 and Δt is the calculated timescales (in days or years). The grey values are used as proxy of Mg# in clinopyroxene following Petrone *et al.*[5]. The temperature at which the diffusion has been calculated is reported in Table 1 for each clinopyroxene. See Method and Materials for further details.

Paroxysm 3 July 2019, Sample P24-3, lp – Px7 – Subgroup 1B

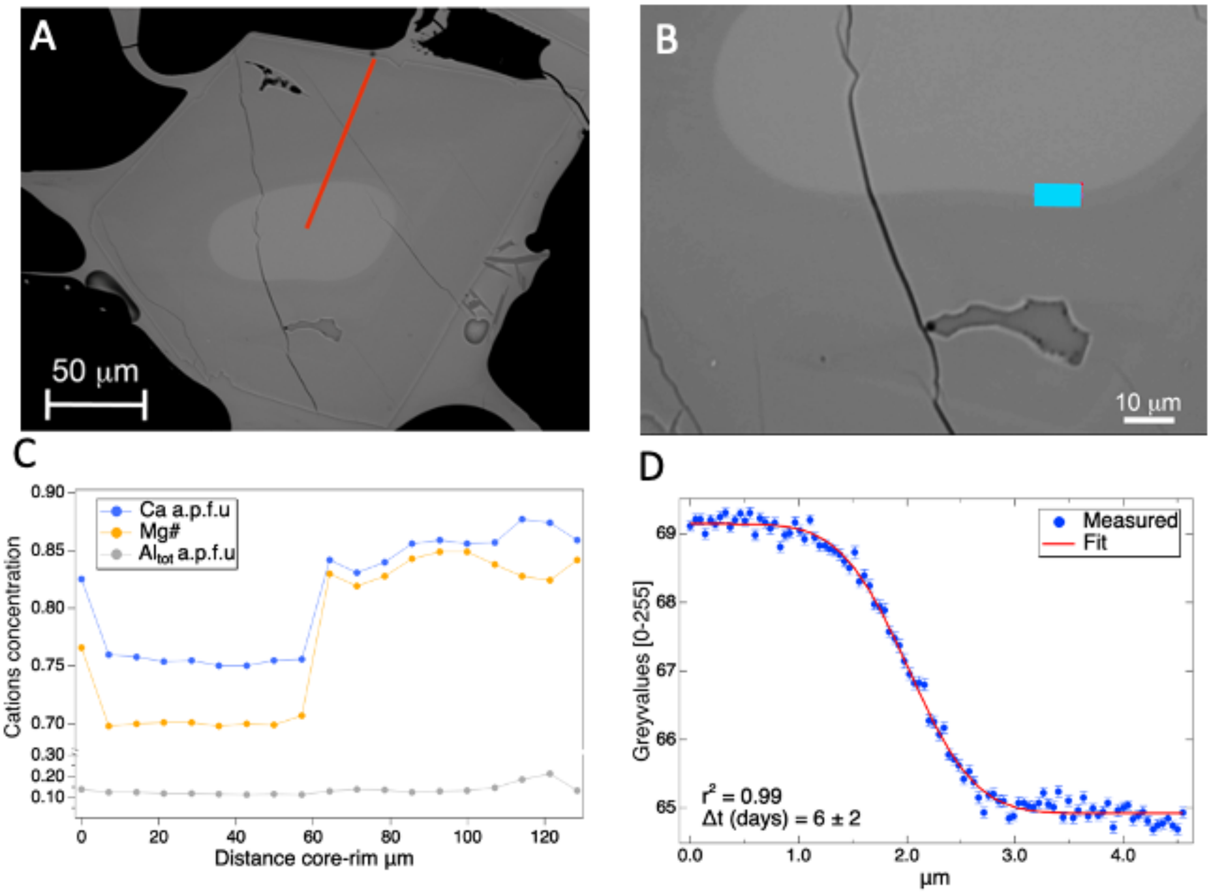

Supplementary Fig. 30.
Clinopyroxene chemical profile and diffusion modeling. (A) SEM-BSE image of the analyzed clinopyroxene. The red line marks the analyzed chemical profile reported in C. (B) SEM-BSE high resolution image showing the area (light blue area) where the grey scale profile for the diffusion profile has been extracted using the *greyvalues* Matlab script of the NIDIS model from Petrone *et al.*[5]. The red dot on the light blue area marks the initial point of the profile. (C) Variation of Ca (blue dots +line), Al (grey dots + line) (a.p.f.u.) and Mg# (orange dots + line) *vs* distance (µm) along the chemical profile shown in (A). (D) Grey values diffusion profile (blue dots with error bar) *vs* distance (µm) extracted along the light blue area in (B). The red line is the fit of the diffusion modeling calculated using the *createfit* Matlab script of the NIDIS model from Petrone *et al.*[5]. The goodness of the fitting is reported as r^2 and Δt is the calculated timescales (in days or years). The grey values are used as proxy of Mg# in clinopyroxene following Petrone *et al.*[5]. The temperature at which the diffusion has been calculated is reported in Table 1 for each clinopyroxene. See Method and Materials for further details.

Paroxysm 3 July 2019, Sample P24-3, lp – Px8 – Subgroup 1B

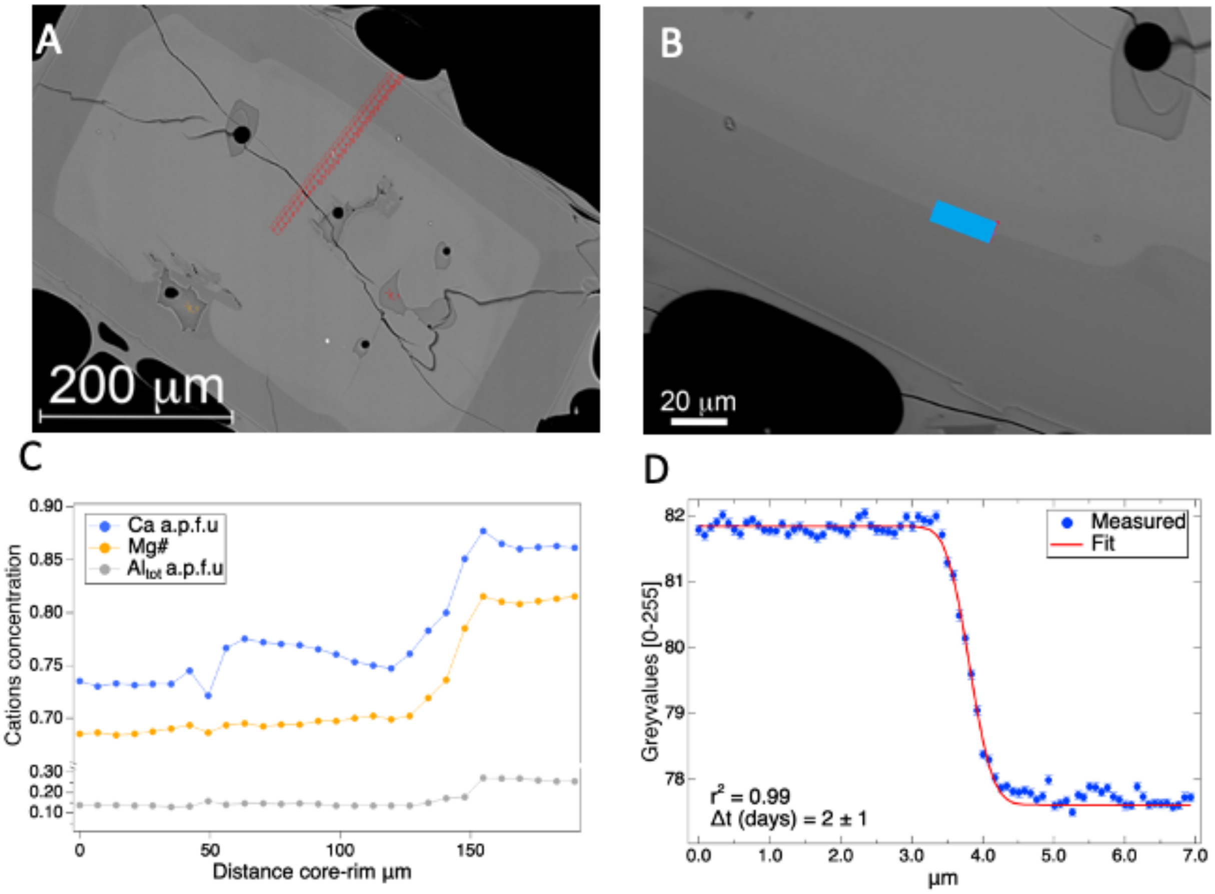

Supplementary Fig. 31.
Clinopyroxene chemical profile and diffusion modeling. (A) SEM-BSE image of the analyzed clinopyroxene. The red line with numbers marks the analyzed chemical profile reported in C. (B) SEM-BSE high resolution image showing the area (light blue area) where the grey scale profile for the diffusion profile has been extracted using the *greyvalues* Matlab script of the NIDIS model from Petrone *et al.*[5]. The red dot on the light blue area marks the initial point of the profile. (C) Variation of Ca (blue dots +line), Al (grey dots + line) (a.p.f.u.) and Mg# (orange dots + line) *vs* distance (µm) along the chemical profile shown in (A). (D) Grey values diffusion profile (blue dots with error bar) *vs* distance (µm) extracted along the light blue area in (B). The red line is the fit of the diffusion modeling calculated using the *createfit* Matlab script of the NIDIS model from Petrone *et al.*[5]. The goodness of the fitting is reported as r^2 and Δt is the calculated timescales (in days or years). The grey values are used as proxy of Mg# in clinopyroxene following Petrone *et al.*[5]. The temperature at which the diffusion has been calculated is reported in Table 1 for each clinopyroxene. See Method and Materials for further details.

Paroxysm 3 July 2019, Sample P30-07, hp – Px28 – Subgroup 1B

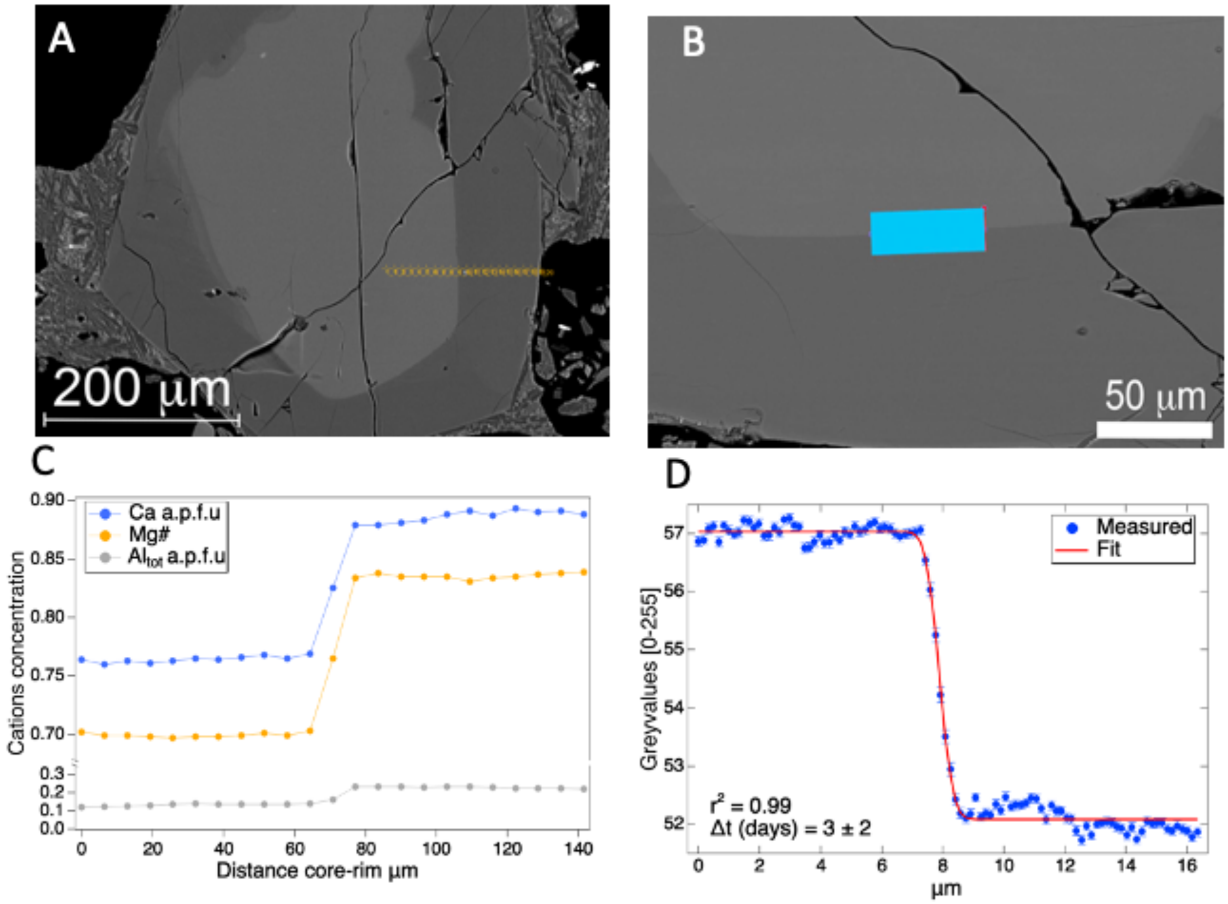

Supplementary Fig. 32.
Clinopyroxene chemical profile and diffusion modeling. (A) SEM-BSE image of the analyzed clinopyroxene. The yellow line with numbers marks the analyzed chemical profile reported in C. (B) SEM-BSE high resolution image showing the area (light blue area) where the grey scale profile for the diffusion profile has been extracted using the *greyvalues* Matlab script of the NIDIS model from Petrone *et al.*[5]. The red dot on the light blue area marks the initial point of the profile. (C) Variation of Ca (blue dots +line), Al (grey dots + line) (a.p.f.u.) and Mg# (orange dots + line) *vs* distance (µm) along the chemical profile shown in (A). (D) Grey values diffusion profile (blue dots with error bar) *vs* distance (µm) extracted along the light blue area in (B). The red line is the fit of the diffusion modeling calculated using the *createfit* Matlab script of the NIDIS model from Petrone *et al.*[5]. The goodness of the fitting is reported as r^2 and Δt is the calculated timescales (in days or years). The grey values are used as proxy of Mg# in clinopyroxene following Petrone *et al.*[5]. The temperature at which the diffusion has been calculated is reported in Table 1 for each clinopyroxene. See Method and Materials for further details.

Paroxysm 3 July 2019, Sample P30-07, hp – Px5 – Subgroup 1B

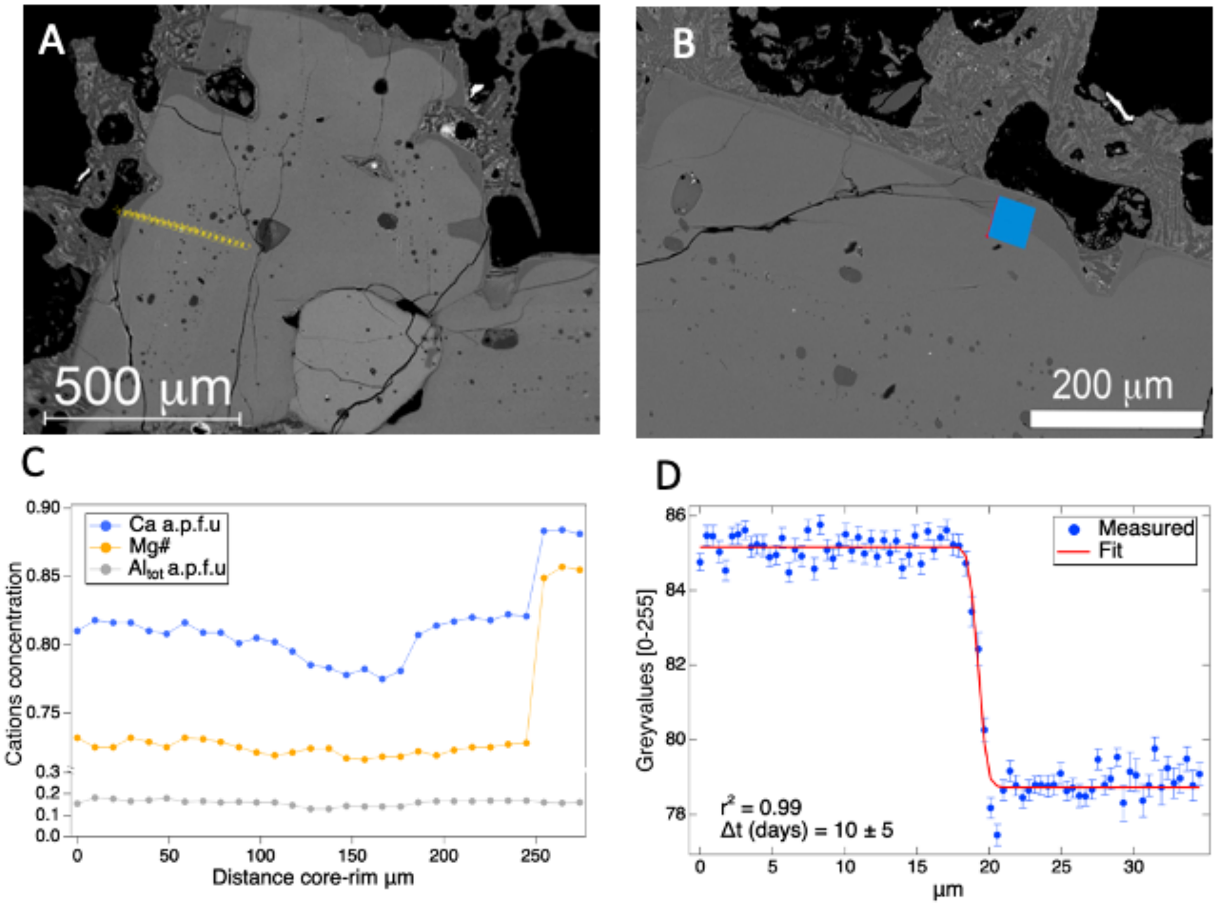

Supplementary Fig. 33.
Clinopyroxene chemical profile and diffusion modeling. (A) SEM-BSE image of the analyzed clinopyroxene. The yellow line with numbers marks the analyzed chemical profile reported in C. (B) SEM-BSE high resolution image showing the area (light blue area) where the grey scale profile for the diffusion profile has been extracted using the *greyvalues* Matlab script of the NIDIS model from Petrone *et al.*[5]. The red dot on the light blue area marks the initial point of the profile. (C) Variation of Ca (blue dots +line), Al (grey dots + line) (a.p.f.u.) and Mg# (orange dots + line) *vs* distance (μm) along the chemical profile shown in (A). (D) Grey values diffusion profile (blue dots with error bar) *vs* distance (μm) extracted along the light blue area in (B). The red line is the fit of the diffusion modeling calculated using the *createfit* Matlab script of the NIDIS model from Petrone *et al.*[5]. The goodness of the fitting is reported as r^2 and Δt is the calculated timescales (in days or years). The grey values are used as proxy of Mg# in clinopyroxene following Petrone *et al.*[5]. The temperature at which the diffusion has been calculated is reported in Table 1 for each clinopyroxene. See Method and Materials for further details.

Paroxysm 3 July 2019, Sample P30-07, hp – Px10 – Subgroup 1B

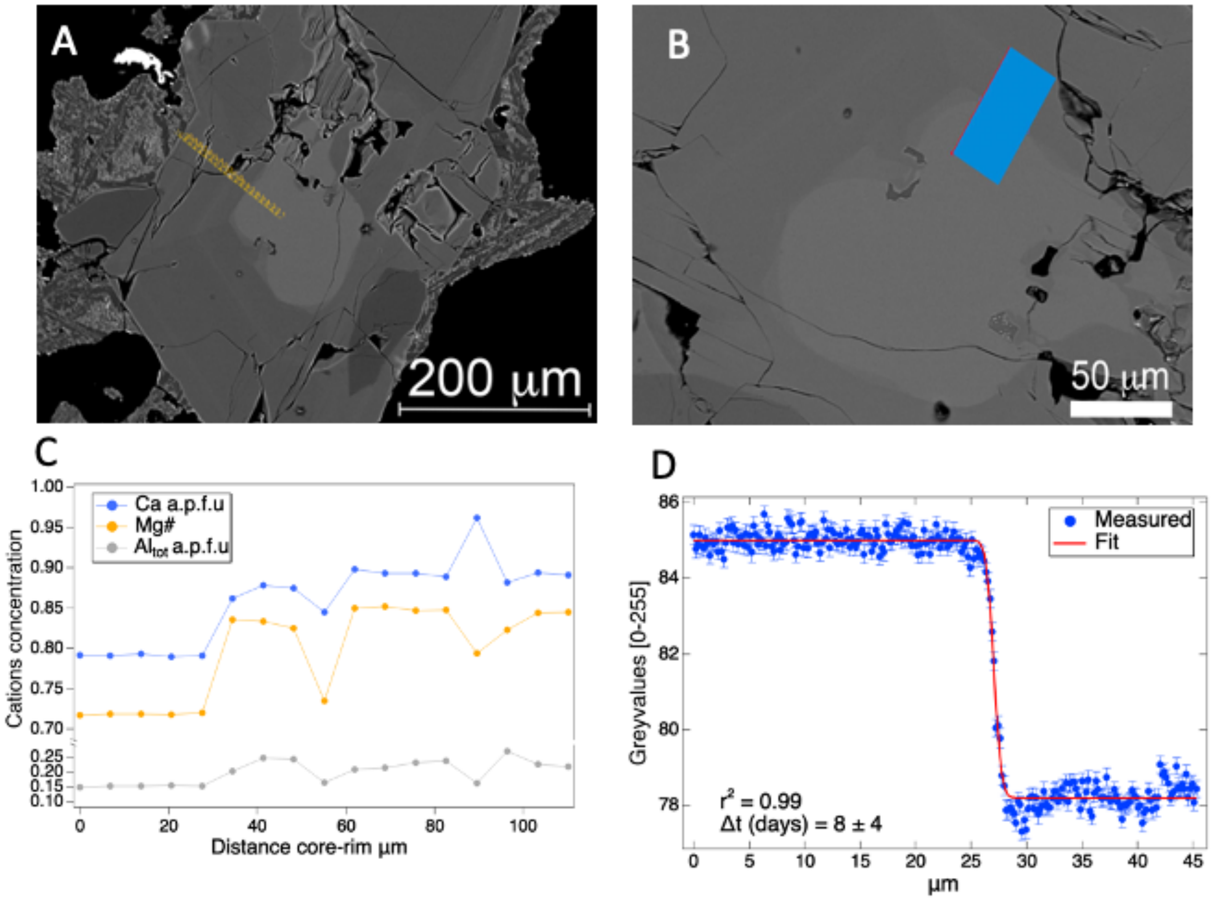

Supplementary Fig. 34.
Clinopyroxene chemical profile and diffusion modeling. (A) SEM-BSE image of the analyzed clinopyroxene. The yellow line with numbers marks the analyzed chemical profile reported in C. (B) SEM-BSE high resolution image showing the area (light blue area) where the grey scale profile for the diffusion profile has been extracted using the *greyvalues* Matlab script of the NIDIS model from Petrone *et al.*[5]. The red dot on the light blue area marks the initial point of the profile. (C) Variation of Ca (blue dots +line), Al (grey dots + line) (a.p.f.u.) and Mg# (orange dots + line) *vs* distance (μm) along the chemical profile shown in (A). (D) Grey values diffusion profile (blue dots with error bar) *vs* distance (μm) extracted along the light blue area in (B). The red line is the fit of the diffusion modeling calculated using the *createfit* Matlab script of the NIDIS model from Petrone *et al.*[5]. The goodness of the fitting is reported as r^2 and Δt is the calculated timescales (in days or years). The grey values are used as proxy of Mg# in clinopyroxene following Petrone *et al.*[5]. The temperature at which the diffusion has been calculated is reported in Table 1 for each clinopyroxene. See Method and Materials for further details.

Paroxysm 3 July 2019, Sample P30-07, hp – Px19 – Subgroup 1B

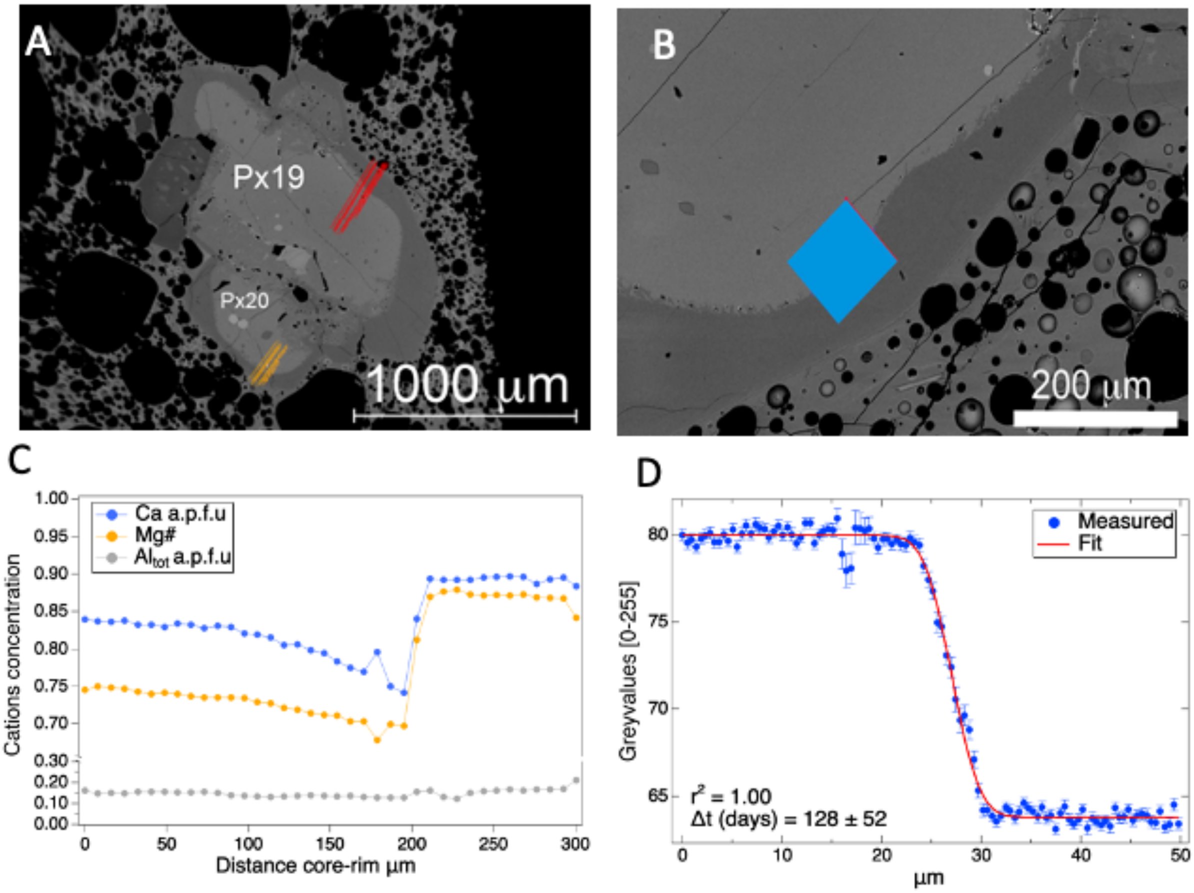

Supplementary Fig. 35.
Clinopyroxene chemical profile and diffusion modeling. (A) SEM-BSE image of the analyzed clinopyroxene. The red line with numbers marks the analyzed chemical profile reported in C. (B) SEM-BSE high resolution image showing the area (light blue area) where the grey scale profile for the diffusion profile has been extracted using the *greyvalues* Matlab script of the NIDIS model from Petrone *et al.*[5]. The red dot on the light blue area marks the initial point of the profile. (C) Variation of Ca (blue dots +line), Al (grey dots + line) (a.p.f.u.) and Mg# (orange dots + line) *vs* distance (µm) along the chemical profile shown in (A). (D) Grey values diffusion profile (blue dots with error bar) *vs* distance (µm) extracted along the light blue area in (B). The red line is the fit of the diffusion modeling calculated using the *createfit* Matlab script of the NIDIS model from Petrone *et al.*[5]. The goodness of the fitting is reported as r^2 and Δt is the calculated timescales (in days or years). The grey values are used as proxy of Mg# in clinopyroxene following Petrone *et al.*[5]. The temperature at which the diffusion has been calculated is reported in Table 1 for each clinopyroxene. See Method and Materials for further details.

Paroxysm 3 July 2019, Sample P30-07, hp – Px20 – Subgroup 1B

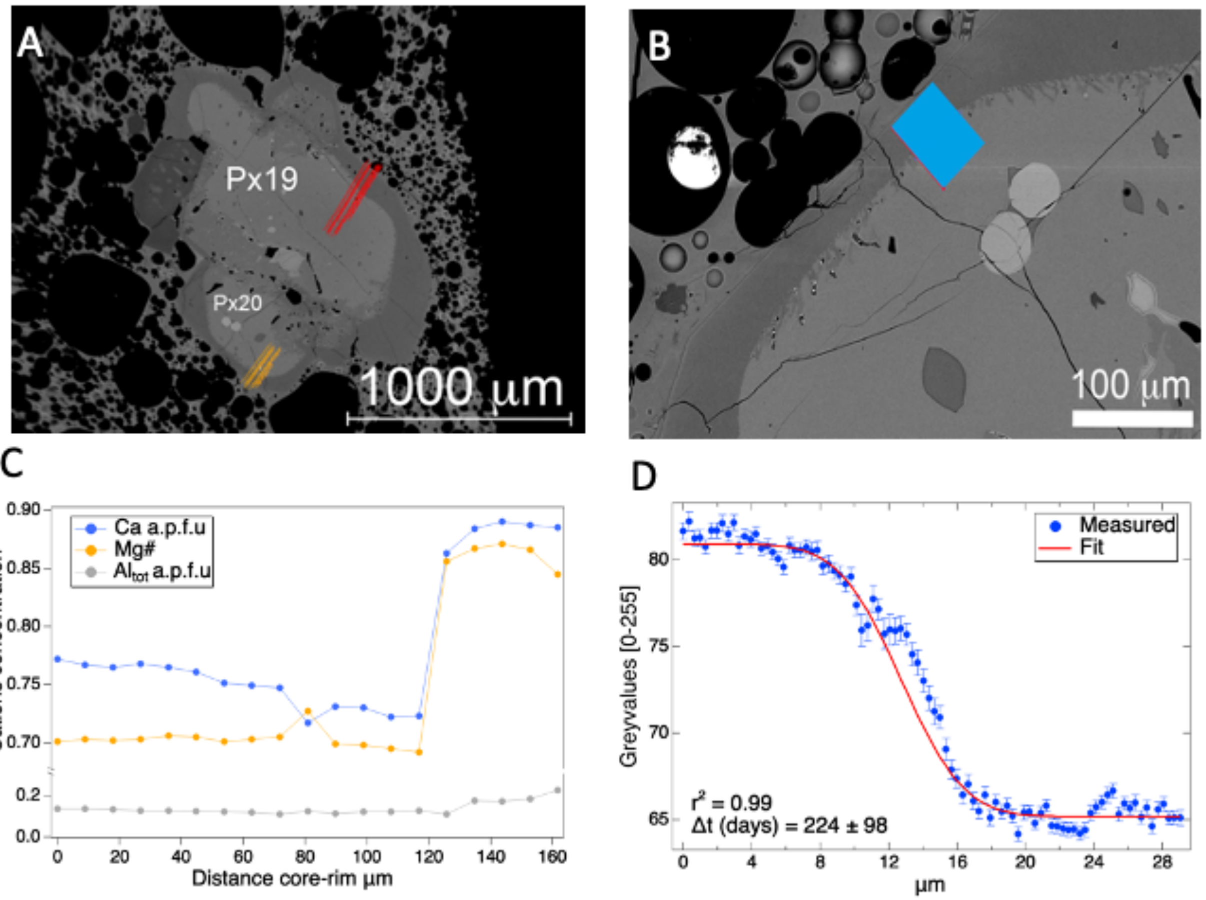

Supplementary Fig. 36.
Clinopyroxene chemical profile and diffusion modeling. (A) SEM-BSE image of the analyzed clinopyroxene. The yellow line with numbers marks the analyzed chemical profile reported in C. (B) SEM-BSE high resolution image showing the area (light blue area) where the grey scale profile for the diffusion profile has been extracted using the *greyvalues* Matlab script of the NIDIS model from Petrone *et al.*[5]. The red dot on the light blue area marks the initial point of the profile. (C) Variation of Ca (blue dots +line), Al (grey dots + line) (a.p.f.u.) and Mg# (orange dots + line) *vs* distance (µm) along the chemical profile shown in (A). (D) Grey values diffusion profile (blue dots with error bar) *vs* distance (µm) extracted along the light blue area in (B). The red line is the fit of the diffusion modeling calculated using the *createfit* Matlab script of the NIDIS model from Petrone *et al.*[5]. The goodness of the fitting is reported as r^2 and Δt is the calculated timescales (in days or years). The grey values are used as proxy of Mg# in clinopyroxene following Petrone *et al.*[5]. The temperature at which the diffusion has been calculated is reported in Table 1 for each clinopyroxene. See Method and Materials for further details.

Paroxysm 28 August 2019, Sample P44-1, lp – Px1 – Subgroup 1B

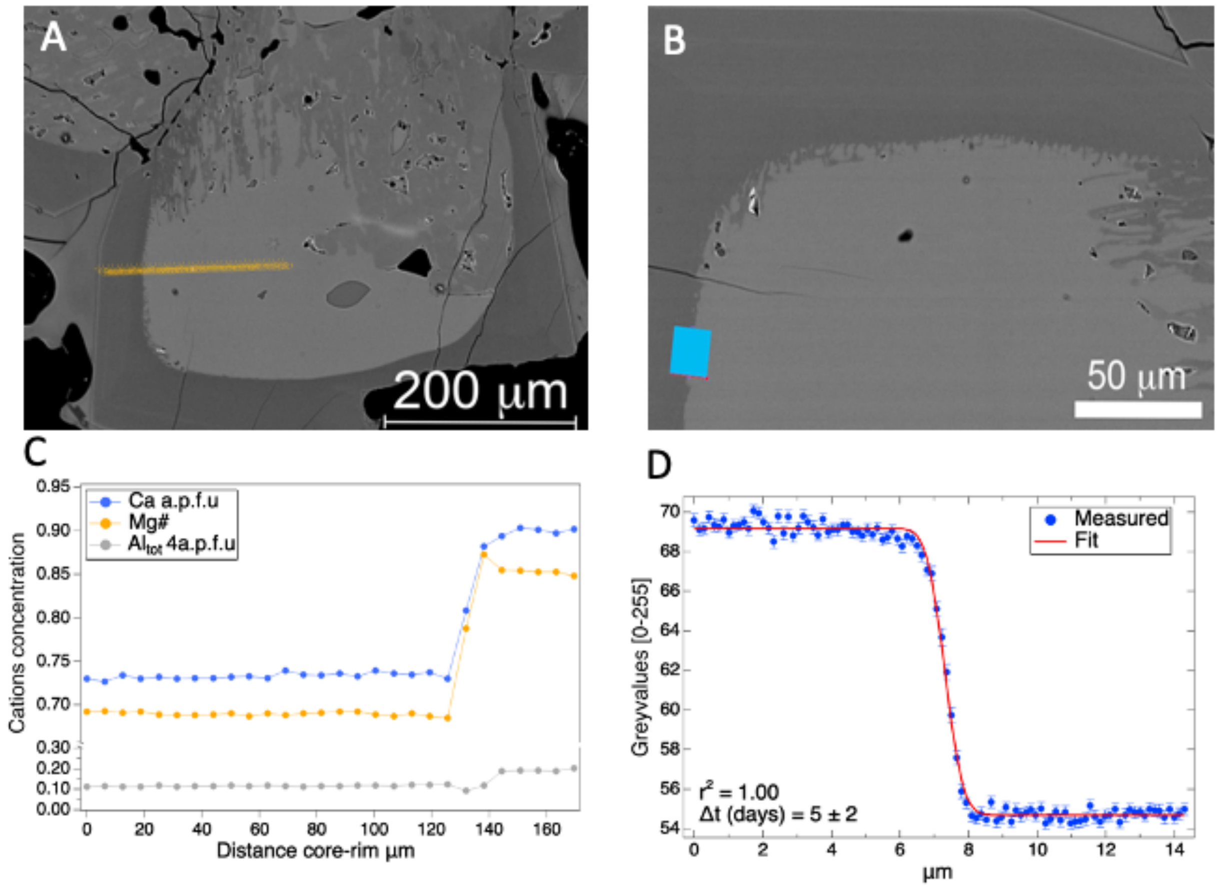

Supplementary Fig. 37.

Clinopyroxene chemical profile and diffusion modeling. (A) SEM-BSE image of the analyzed clinopyroxene. The yellow line with numbers marks the analyzed chemical profile reported in C. (B) SEM-BSE high resolution image showing the area (light blue area) where the grey scale profile for the diffusion profile has been extracted using the *greyvalues* Matlab script of the NIDIS model from Petrone *et al.*[5]. The red dot on the light blue area marks the initial point of the profile. (C) Variation of Ca (blue dots +line), Al (grey dots + line) (a.p.f.u.) and Mg# (orange dots + line) *vs* distance (µm) along the chemical profile shown in (A). (D) Grey values diffusion profile (blue dots with error bar) *vs* distance (µm) extracted along the light blue area in (B). The red line is the fit of the diffusion modeling calculated using the *createfit* Matlab script of the NIDIS model from Petrone *et al.*[5]. The goodness of the fitting is reported as r^2 and Δt is the calculated timescales (in days or years). The grey values are used as proxy of Mg# in clinopyroxene following Petrone *et al.*[5]. The temperature at which the diffusion has been calculated is reported in Table 1 for each clinopyroxene. See Method and Materials for further details.

Paroxysm 28 August 2019, Sample P44-1, lp – Px2 – Subgroup 1B

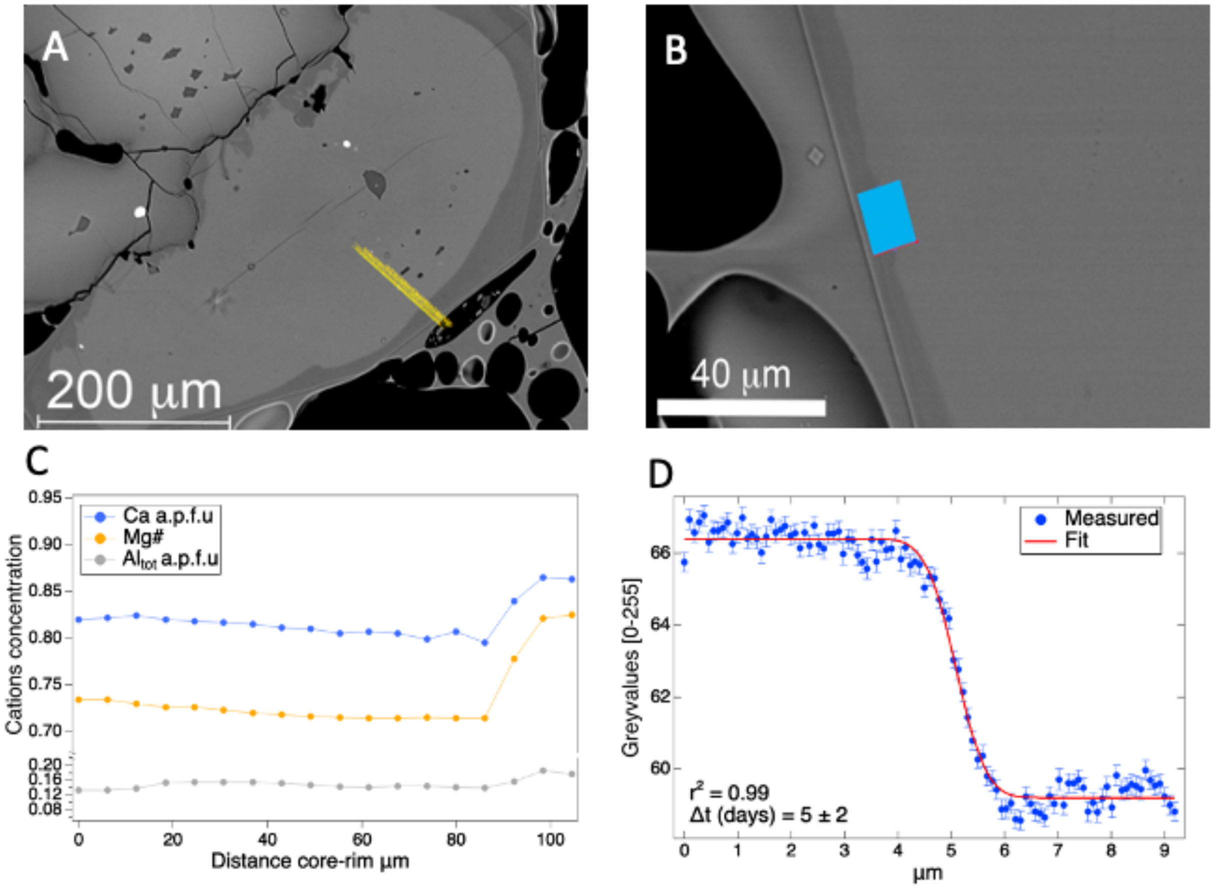

Supplementary Fig. 38.

Clinopyroxene chemical profile and diffusion modeling. (A) SEM-BSE image of the analyzed clinopyroxene. The yellow line with numbers marks the analyzed chemical profile reported in C. (B) SEM-BSE high resolution image showing the area (light blue area) where the grey scale profile for the diffusion profile has been extracted using the *greyvalues* Matlab script of the NIDIS model from Petrone *et al.*[5]. The red dot on the light blue area marks the initial point of the profile. (C) Variation of Ca (blue dots +line), Al (grey dots + line) (a.p.f.u.) and Mg# (orange dots + line) *vs* distance (µm) along the chemical profile shown in (A). (D) Grey values diffusion profile (blue dots with error bar) *vs* distance (µm) extracted along the light blue area in (B). The red line is the fit of the diffusion modeling calculated using the *createfit* Matlab script of the NIDIS model from Petrone *et al.*[5]. The goodness of the fitting is reported as r^2 and Δt is the calculated timescales (in days or years). The grey values are used as proxy of Mg# in clinopyroxene following Petrone *et al.*[5]. The temperature at which the diffusion has been calculated is reported in Table 1 for each clinopyroxene. See Method and Materials for further details.

Paroxysm 28 August 2019, Sample P44-1, lp – Px3 – Subgroup 1B

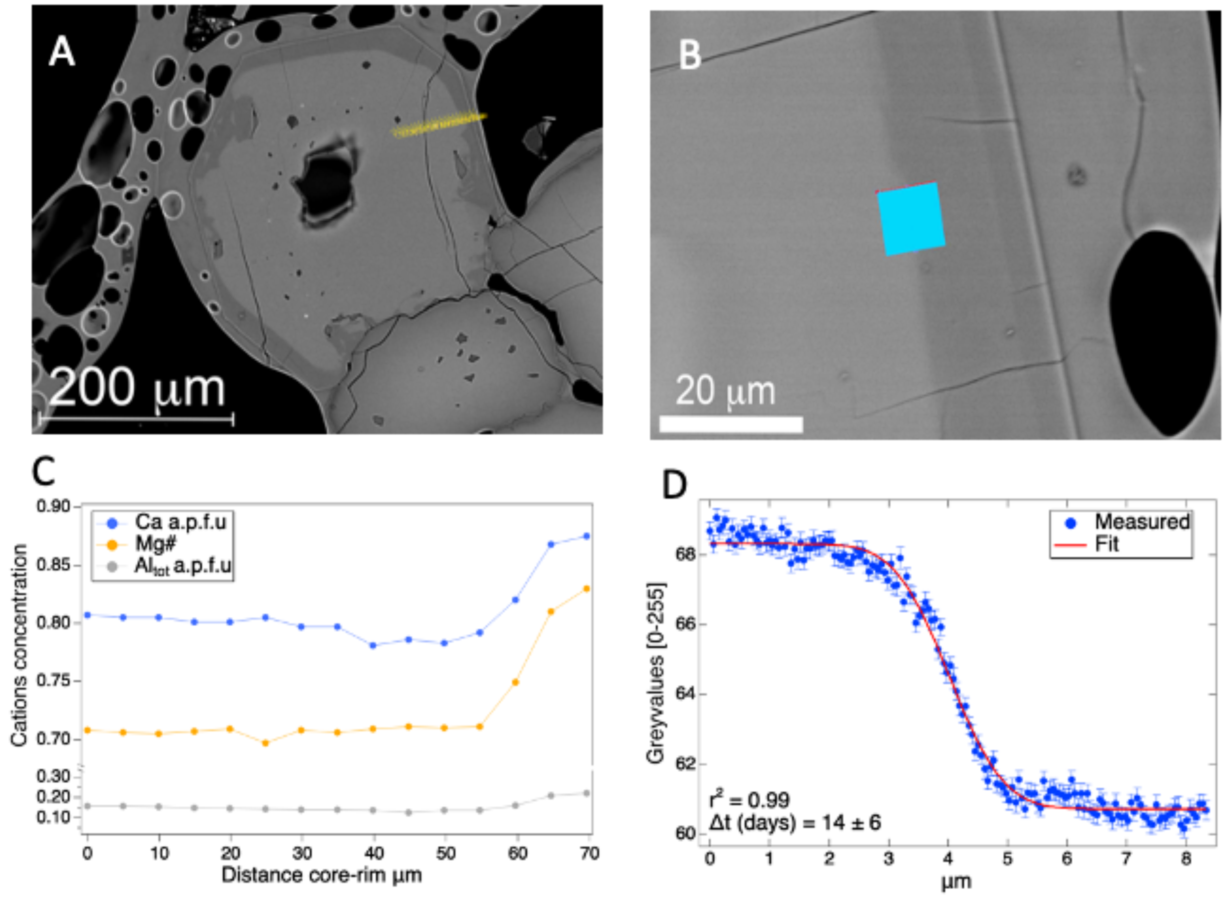

Supplementary Fig. 39.
Clinopyroxene chemical profile and diffusion modeling. (A) SEM-BSE image of the analyzed clinopyroxene. The yellow line with numbers marks the analyzed chemical profile reported in C. (B) SEM-BSE high resolution image showing the area (light blue area) where the grey scale profile for the diffusion profile has been extracted using the *greyvalues* Matlab script of the NIDIS model from Petrone *et al.*[5]. The red dot on the light blue area marks the initial point of the profile. (C) Variation of Ca (blue dots +line), Al (grey dots + line) (a.p.f.u.) and Mg# (orange dots + line) *vs* distance (µm) along the chemical profile shown in (A). (D) Grey values diffusion profile (blue dots with error bar) *vs* distance (µm) extracted along the light blue area in (B). The red line is the fit of the diffusion modeling calculated using the *createfit* Matlab script of the NIDIS model from Petrone *et al.*[5]. The goodness of the fitting is reported as r^2 and Δt is the calculated timescales (in days or years). The grey values are used as proxy of Mg# in clinopyroxene following Petrone *et al.*[5]. The temperature at which the diffusion has been calculated is reported in Table 1 for each clinopyroxene. See Method and Materials for further details.

Paroxysm 28 August 2019, Sample P44-1, lp – Px4 – Subgroup 1B

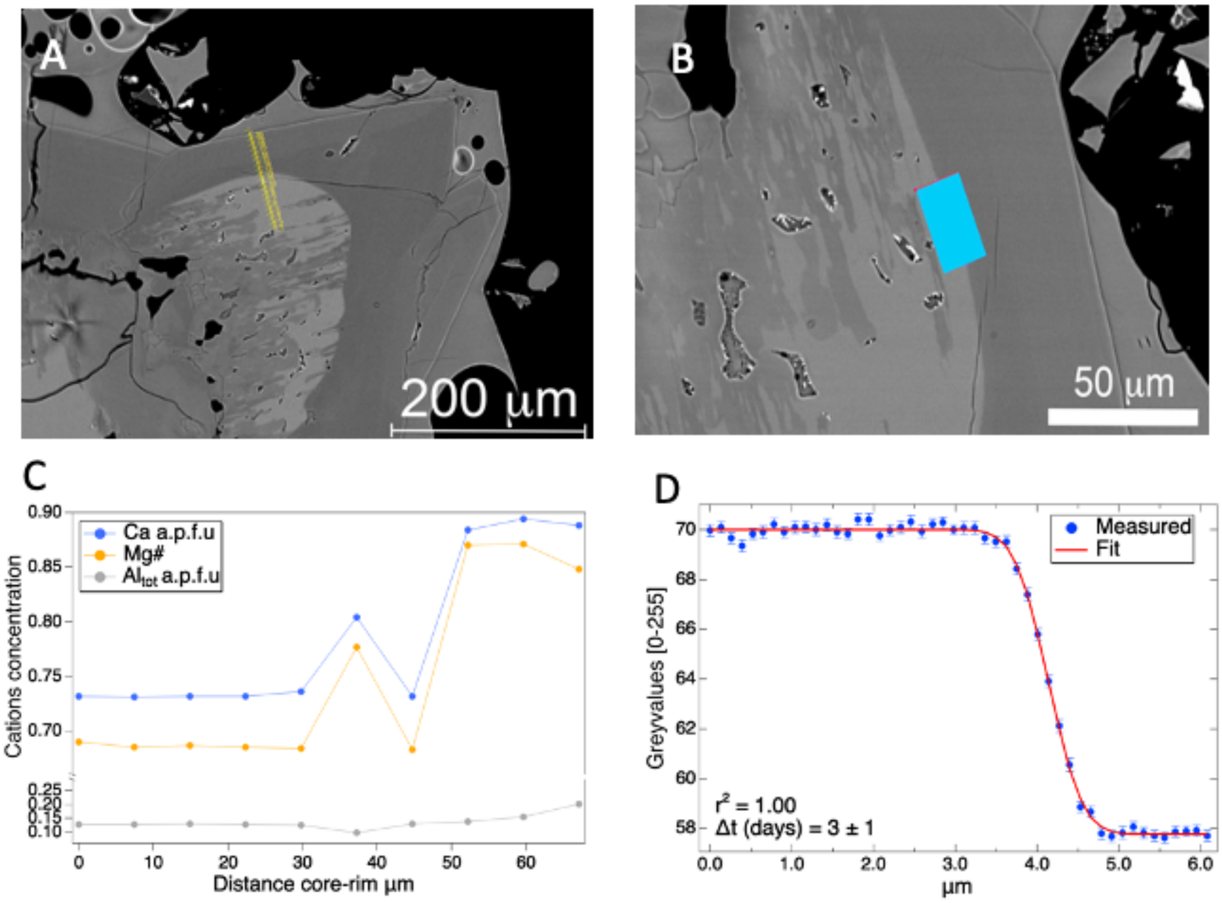

Supplementary Fig. 40.
Clinopyroxene chemical profile and diffusion modeling. (A) SEM-BSE image of the analyzed clinopyroxene. The yellow line with numbers marks the analyzed chemical profile reported in C. (B) SEM-BSE high resolution image showing the area (light blue area) where the grey scale profile for the diffusion profile has been extracted using the *greyvalues* Matlab script of the NIDIS model from Petrone *et al.*[5]. The red dot on the light blue area marks the initial point of the profile. (C) Variation of Ca (blue dots +line), Al (grey dots + line) (a.p.f.u.) and Mg# (orange dots + line) *vs* distance (µm) along the chemical profile shown in (A). (D) Grey values diffusion profile (blue dots with error bar) *vs* distance (µm) extracted along the light blue area in (B). The red line is the fit of the diffusion modeling calculated using the *createfit* Matlab script of the NIDIS model from Petrone *et al.*[5]. The goodness of the fitting is reported as r^2 and Δt is the calculated timescales (in days or years). The grey values are used as proxy of Mg# in clinopyroxene following Petrone *et al.*[5]. The temperature at which the diffusion has been calculated is reported in Table 1 for each clinopyroxene. See Method and Materials for further details.

Paroxysm 28 August 2019, Sample P44-1, lp – Px11 – Subgroup 1B

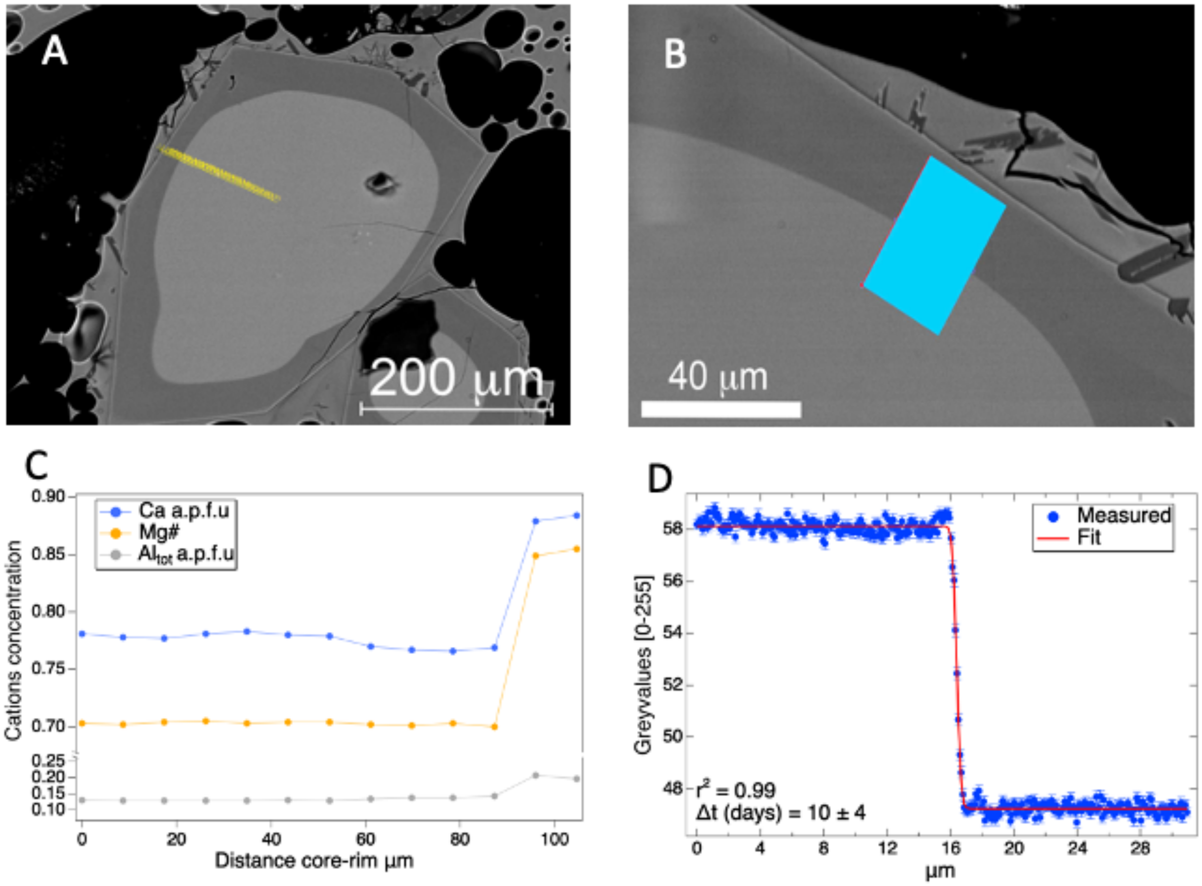

Supplementary Fig. 41.

Clinopyroxene chemical profile and diffusion modeling. (A) SEM-BSE image of the analyzed clinopyroxene. The yellow line with numbers marks the analyzed chemical profile reported in C. (B) SEM-BSE high resolution image showing the area (light blue area) where the grey scale profile for the diffusion profile has been extracted using the *greyvalues* Matlab script of the NIDIS model from Petrone *et al.*[5]. The red dot on the light blue area marks the initial point of the profile. (C) Variation of Ca (blue dots +line), Al (grey dots + line) (a.p.f.u.) and Mg# (orange dots + line) *vs* distance (µm) along the chemical profile shown in (A). (D) Grey values diffusion profile (blue dots with error bar) *vs* distance (µm) extracted along the light blue area in (B). The red line is the fit of the diffusion modeling calculated using the *createfit* Matlab script of the NIDIS model from Petrone *et al.*[5]. The goodness of the fitting is reported as r^2 and Δt is the calculated timescales (in days or years). The grey values are used as proxy of Mg# in clinopyroxene following Petrone *et al.*[5]. The temperature at which the diffusion has been calculated is reported in Table 1 for each clinopyroxene. See Method and Materials for further details.

Paroxysm 28 August 2019, Sample P47-1, lp – Px2 – Subgroup 1B

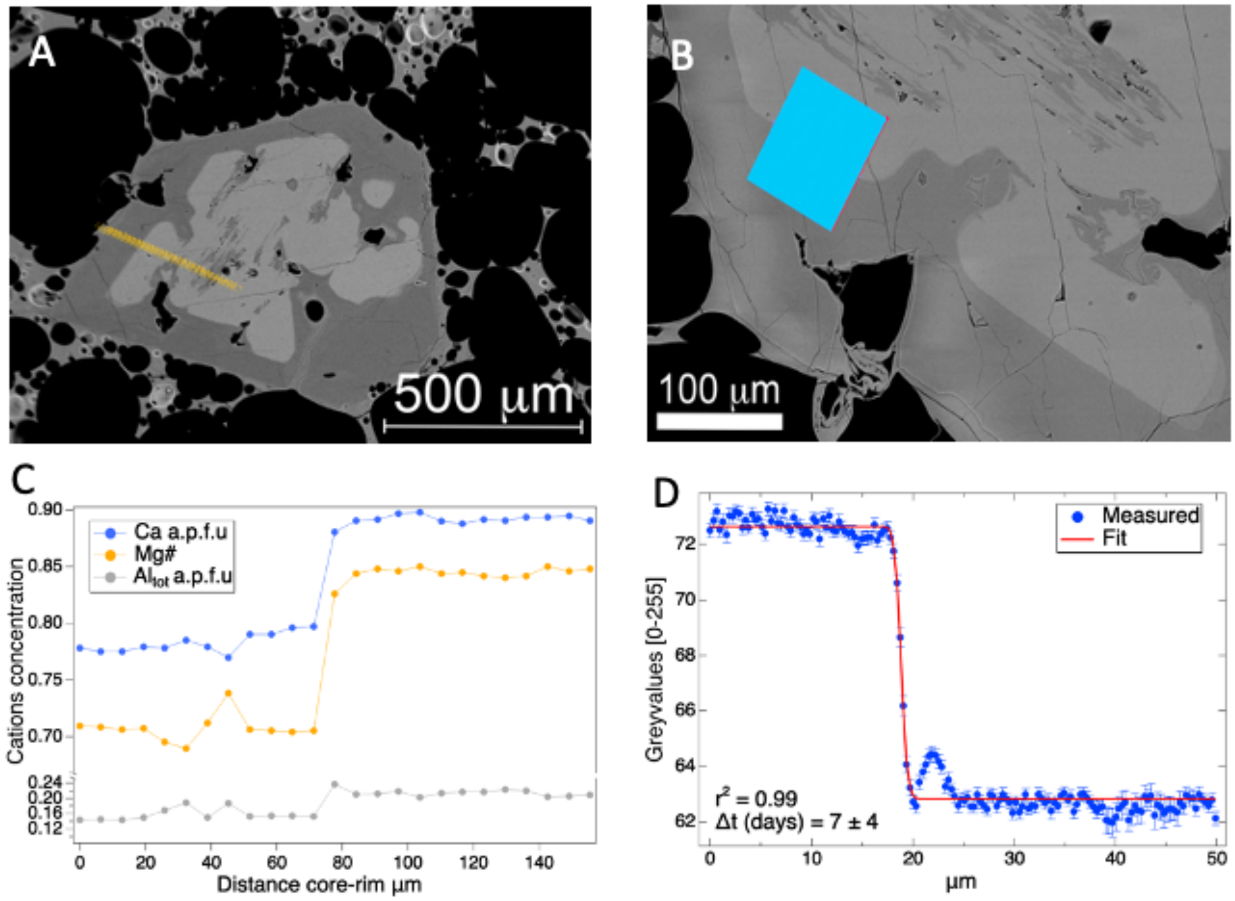

Supplementary Fig. 42.
Clinopyroxene chemical profile and diffusion modeling. (A) SEM-BSE image of the analyzed clinopyroxene. The yellow line with numbers marks the analyzed chemical profile reported in C. (B) SEM-BSE high resolution image showing the area (light blue area) where the grey scale profile for the diffusion profile has been extracted using the *greyvalues* Matlab script of the NIDIS model from Petrone *et al.*[5]. The red dot on the light blue area marks the initial point of the profile. (C) Variation of Ca (blue dots +line), Al (grey dots + line) (a.p.f.u.) and Mg# (orange dots + line) *vs* distance (µm) along the chemical profile shown in (A). (D) Grey values diffusion profile (blue dots with error bar) *vs* distance (µm) extracted along the light blue area in (B). The red line is the fit of the diffusion modeling calculated using the *createfit* Matlab script of the NIDIS model from Petrone *et al.*[5]. The goodness of the fitting is reported as r^2 and Δt is the calculated timescales (in days or years). The grey values are used as proxy of Mg# in clinopyroxene following Petrone *et al.*[5]. The temperature at which the diffusion has been calculated is reported in Table 1 for each clinopyroxene. See Method and Materials for further details.

Paroxysm 28 August 2019, Sample P47-1, lp – Px6 – Subgroup 1B

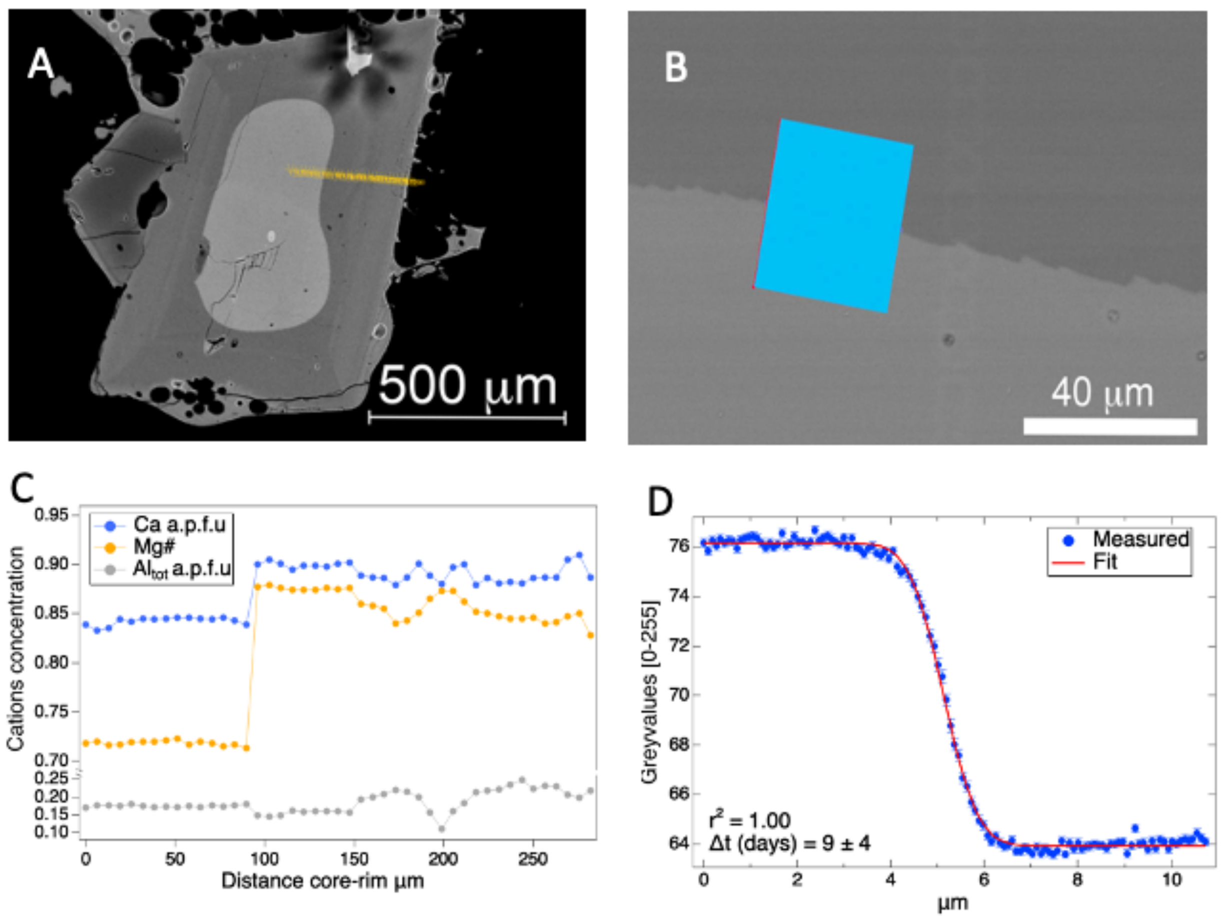

Supplementary Fig. 43.
Clinopyroxene chemical profile and diffusion modeling. (A) SEM-BSE image of the analyzed clinopyroxene. The yellow line with numbers marks the analyzed chemical profile reported in C. (B) SEM-BSE high resolution image showing the area (light blue area) where the grey scale profile for the diffusion profile has been extracted using the *greyvalues* Matlab script of the NIDIS model from Petrone *et al.*[5]. The red dot on the light blue area marks the initial point of the profile. (C) Variation of Ca (blue dots +line), Al (grey dots + line) (a.p.f.u.) and Mg# (orange dots + line) *vs* distance (µm) along the chemical profile shown in (A). (D) Grey values diffusion profile (blue dots with error bar) *vs* distance (µm) extracted along the light blue area in (B). The red line is the fit of the diffusion modeling calculated using the *createfit* Matlab script of the NIDIS model from Petrone *et al.*[5]. The goodness of the fitting is reported as r^2 and Δt is the calculated timescales (in days or years). The grey values are used as proxy of Mg# in clinopyroxene following Petrone *et al.*[5]. The temperature at which the diffusion has been calculated is reported in Table 1 for each clinopyroxene. See Method and Materials for further details.

Paroxysm 28 August 2019, Sample P47-1, lp – Px11 – Subgroup 1B

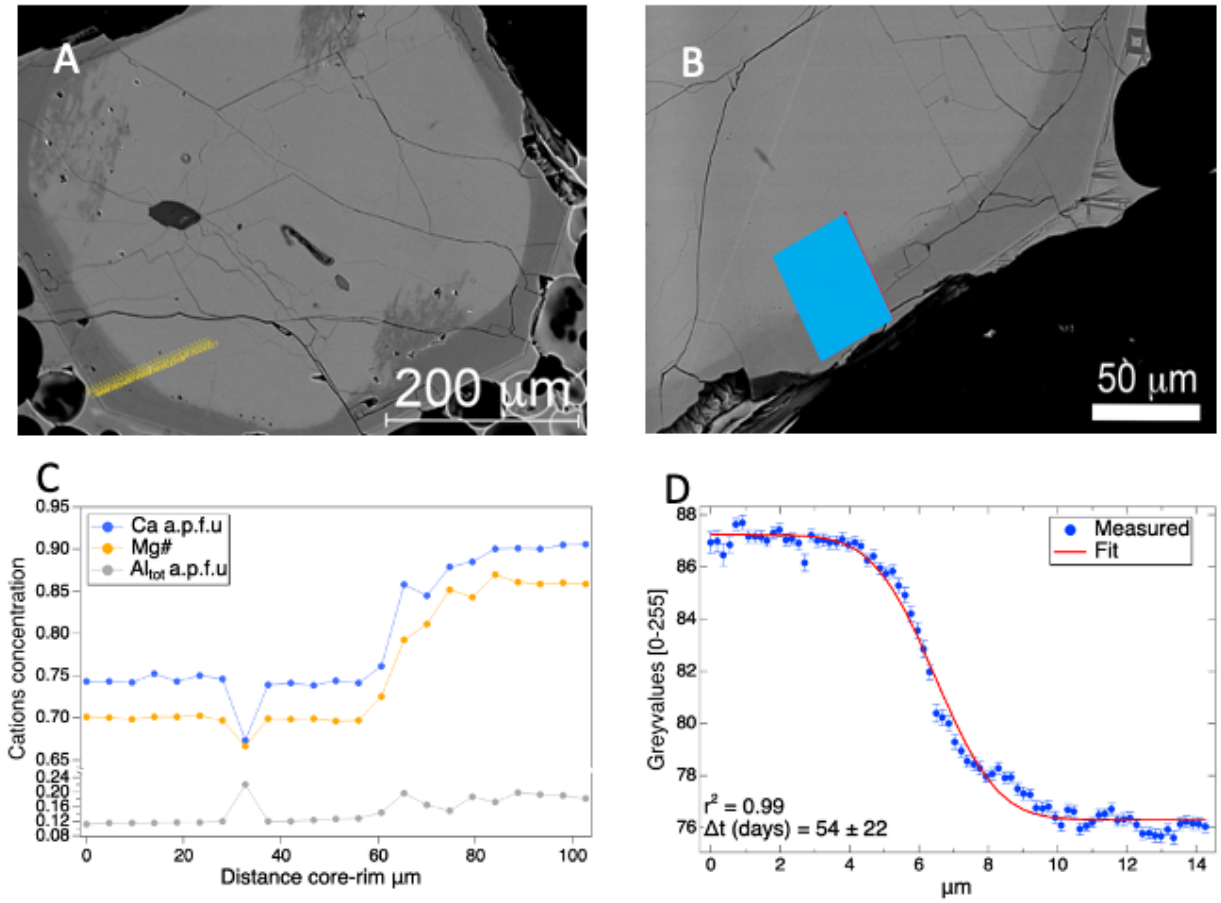

Supplementary Fig. 44.
Clinopyroxene chemical profile and diffusion modeling. (A) SEM-BSE image of the analyzed clinopyroxene. The yellow line with numbers marks the analyzed chemical profile reported in C. (B) SEM-BSE high resolution image showing the area (light blue area) where the grey scale profile for the diffusion profile has been extracted using the *greyvalues* Matlab script of the NIDIS model from Petrone *et al.*[5]. The red dot on the light blue area marks the initial point of the profile. (C) Variation of Ca (blue dots +line), Al (grey dots + line) (a.p.f.u.) and Mg# (orange dots + line) *vs* distance (µm) along the chemical profile shown in (A). (D) Grey values diffusion profile (blue dots with error bar) *vs* distance (µm) extracted along the light blue area in (B). The red line is the fit of the diffusion modeling calculated using the *createfit* Matlab script of the NIDIS model from Petrone *et al.*[5]. The goodness of the fitting is reported as r^2 and Δt is the calculated timescales (in days or years). The grey values are used as proxy of Mg# in clinopyroxene following Petrone *et al.*[5]. The temperature at which the diffusion has been calculated is reported in Table 1 for each clinopyroxene. See Method and Materials for further details.

Paroxysm 28 August 2019, Sample P47-1, lp – Px12 – Subgroup 1B

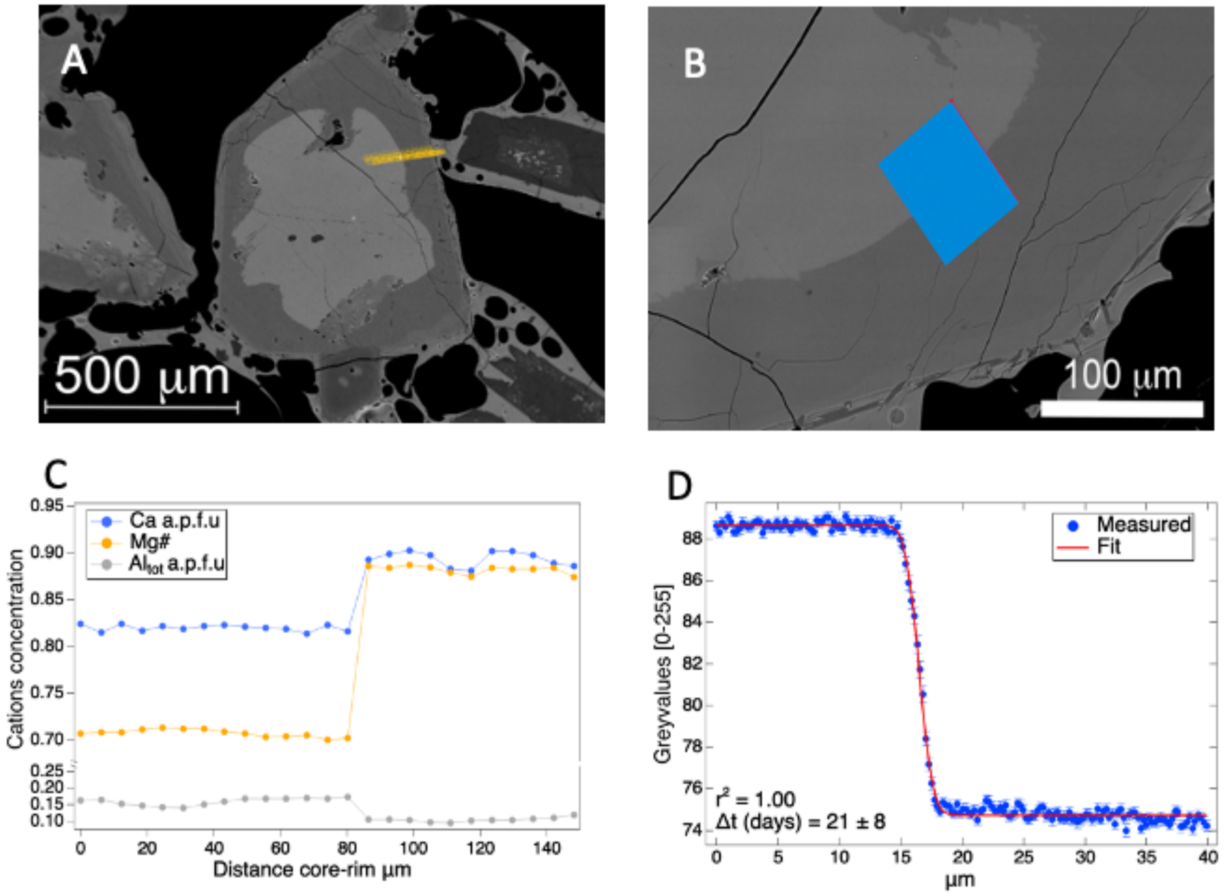

Supplementary Fig. 45.
Clinopyroxene chemical profile and diffusion modeling. (A) SEM-BSE image of the analyzed clinopyroxene. The yellow line with numbers marks the analyzed chemical profile reported in C. (B) SEM-BSE high resolution image showing the area (light blue area) where the grey scale profile for the diffusion profile has been extracted using the *greyvalues* Matlab script of the NIDIS model from Petrone *et al.*[5]. The red dot on the light blue area marks the initial point of the profile. (C) Variation of Ca (blue dots +line), Al (grey dots + line) (a.p.f.u.) and Mg# (orange dots + line) *vs* distance (μm) along the chemical profile shown in (A). (D) Grey values diffusion profile (blue dots with error bar) *vs* distance (μm) extracted along the light blue area in (B). The red line is the fit of the diffusion modeling calculated using the *createfit* Matlab script of the NIDIS model from Petrone *et al.*[5]. The goodness of the fitting is reported as r^2 and Δt is the calculated timescales (in days or years). The grey values are used as proxy of Mg# in clinopyroxene following Petrone *et al.*[5]. The temperature at which the diffusion has been calculated is reported in Table 1 for each clinopyroxene. See Method and Materials for further details.

Paroxysm 28 August 2019, Sample P47-1, lp – Px13 – Subgroup 1B

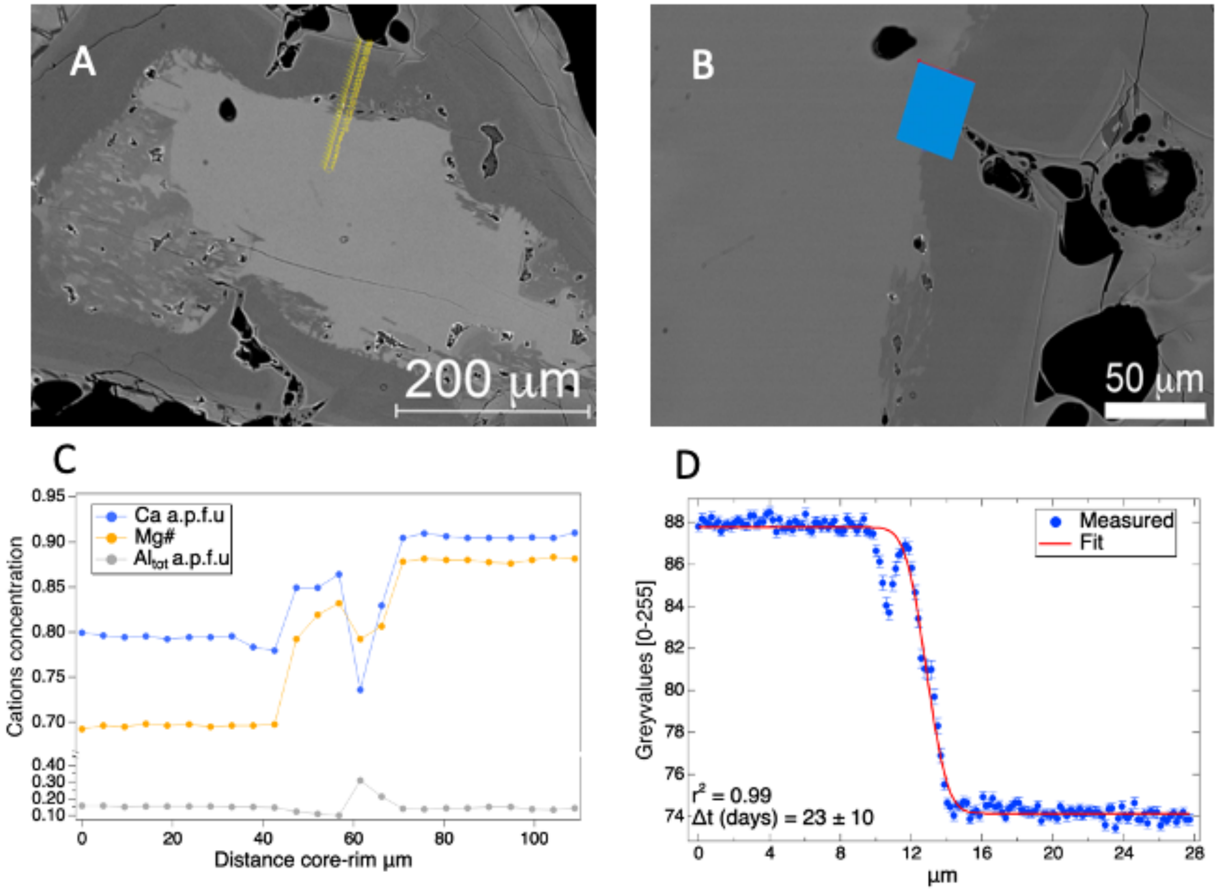

Supplementary Fig. 46.
Clinopyroxene chemical profile and diffusion modeling. (A) SEM-BSE image of the analyzed clinopyroxene. The yellow line with numbers marks the analyzed chemical profile reported in C. (B) SEM-BSE high resolution image showing the area (light blue area) where the grey scale profile for the diffusion profile has been extracted using the *greyvalues* Matlab script of the NIDIS model from Petrone *et al.*[5]. The red dot on the light blue area marks the initial point of the profile. (C) Variation of Ca (blue dots +line), Al (grey dots + line) (a.p.f.u.) and Mg# (orange dots + line) *vs* distance (µm) along the chemical profile shown in (A). (D) Grey values diffusion profile (blue dots with error bar) *vs* distance (µm) extracted along the light blue area in (B). The red line is the fit of the diffusion modeling calculated using the *createfit* Matlab script of the NIDIS model from Petrone *et al.*[5]. The goodness of the fitting is reported as r^2 and Δt is the calculated timescales (in days or years). The grey values are used as proxy of Mg# in clinopyroxene following Petrone *et al.*[5]. The temperature at which the diffusion has been calculated is reported in Table 1 for each clinopyroxene. See Method and Materials for further details.

Paroxysm 28 August 2019, Sample P47-1, lp – Px14 – Subgroup 1B

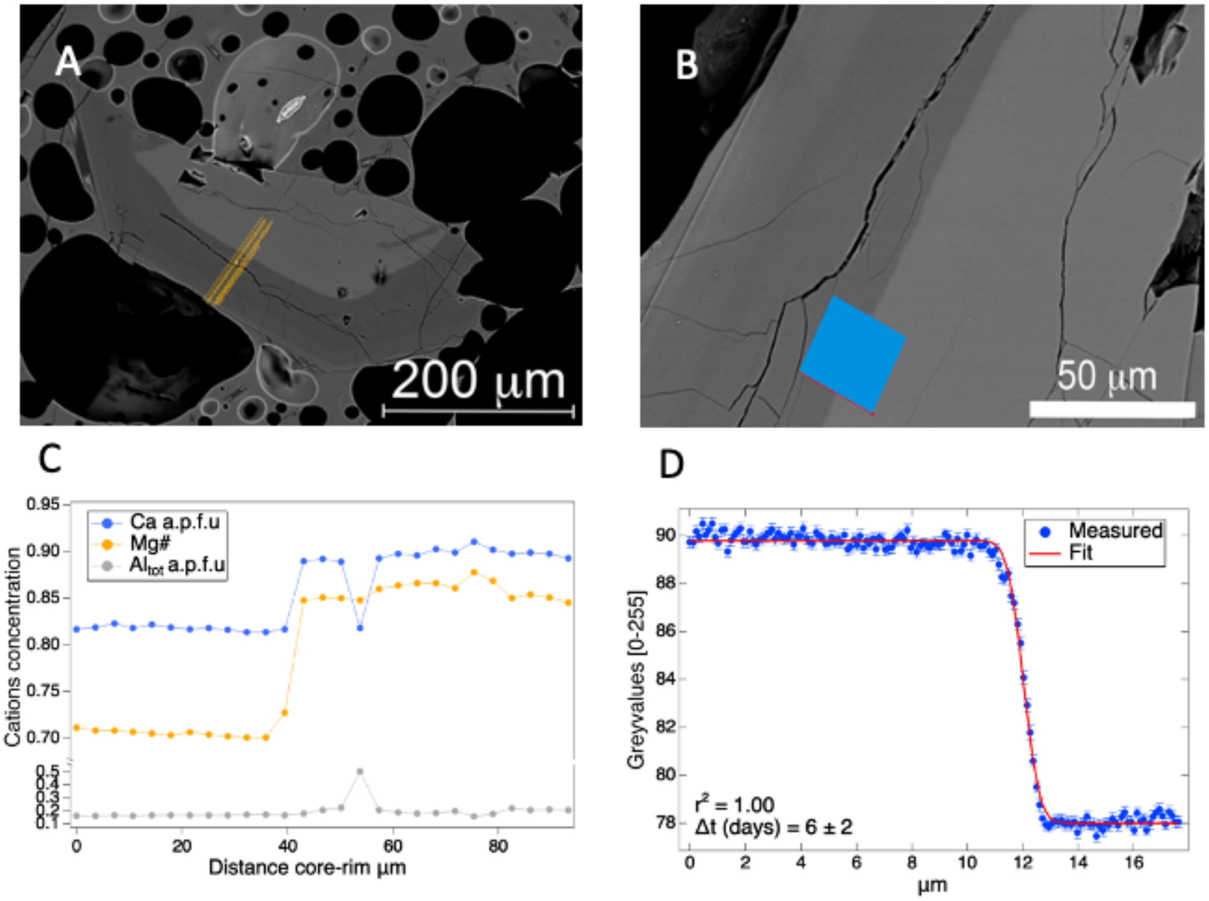

Supplementary Fig. 47.
Clinopyroxene chemical profile and diffusion modeling. (A) SEM-BSE image of the analyzed clinopyroxene. The yellow line with numbers marks the analyzed chemical profile reported in C. (B) SEM-BSE high resolution image showing the area (light blue area) where the grey scale profile for the diffusion profile has been extracted using the *greyvalues* Matlab script of the NIDIS model from Petrone *et al.*[5]. The red dot on the light blue area marks the initial point of the profile. (C) Variation of Ca (blue dots +line), Al (grey dots + line) (a.p.f.u.) and Mg# (orange dots + line) *vs* distance (µm) along the chemical profile shown in (A). (D) Grey values diffusion profile (blue dots with error bar) *vs* distance (µm) extracted along the light blue area in (B). The red line is the fit of the diffusion modeling calculated using the *createfit* Matlab script of the NIDIS model from Petrone *et al.*[5]. The goodness of the fitting is reported as r^2 and Δt is the calculated timescales (in days or years). The grey values are used as proxy of Mg# in clinopyroxene following Petrone *et al.*[5]. The temperature at which the diffusion has been calculated is reported in Table 1 for each clinopyroxene. See Method and Materials for further details.

Paroxysm 28 August 2019, Sample P47-1, lp – Px15 – Subgroup 1B

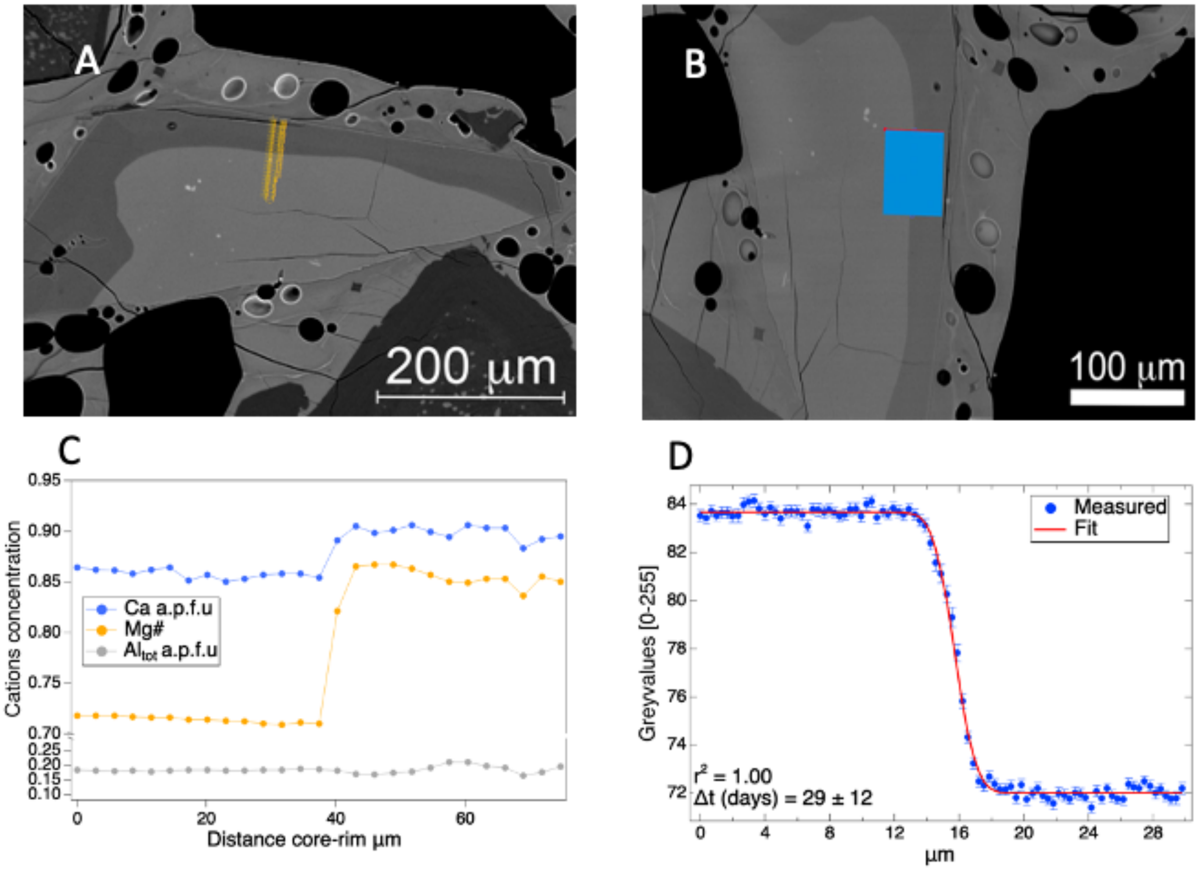

Supplementary Fig. 48.
Clinopyroxene chemical profile and diffusion modeling. (A) SEM-BSE image of the analyzed clinopyroxene. The yellow line with numbers marks the analyzed chemical profile reported in C. (B) SEM-BSE high resolution image showing the area (light blue area) where the grey scale profile for the diffusion profile has been extracted using the *greyvalues* Matlab script of the NIDIS model from Petrone *et al.*[5]. The red dot on the light blue area marks the initial point of the profile. (C) Variation of Ca (blue dots +line), Al (grey dots + line) (a.p.f.u.) and Mg# (orange dots + line) *vs* distance (µm) along the chemical profile shown in (A). (D) Grey values diffusion profile (blue dots with error bar) *vs* distance (µm) extracted along the light blue area in (B). The red line is the fit of the diffusion modeling calculated using the *createfit* Matlab script of the NIDIS model from Petrone *et al.*[5]. The goodness of the fitting is reported as r^2 and Δt is the calculated timescales (in days or years). The grey values are used as proxy of Mg# in clinopyroxene following Petrone *et al.*[5]. The temperature at which the diffusion has been calculated is reported in Table 1 for each clinopyroxene. See Method and Materials for further details.

Paroxysm 28 August 2019, Sample P47-1, lp – Px16 – Subgroup 1B

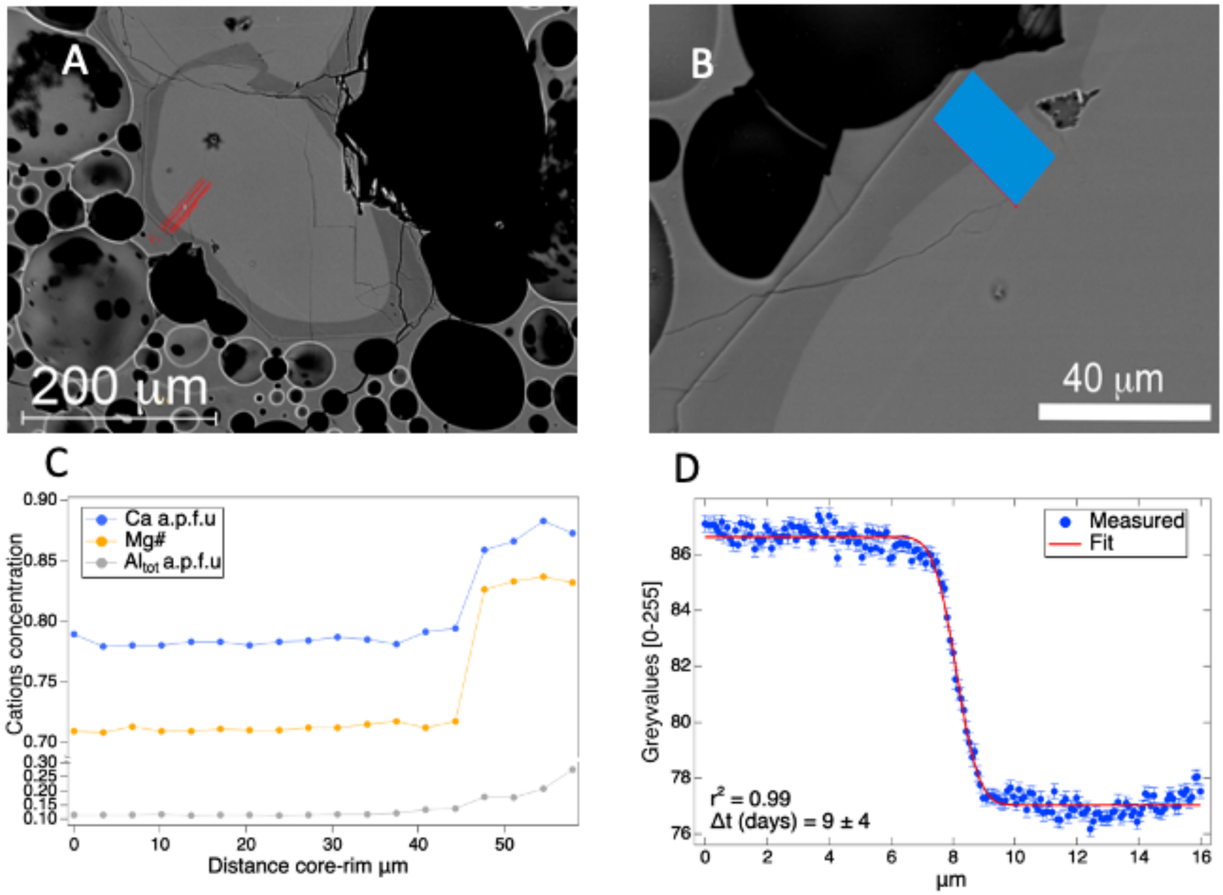

Supplementary Fig. 49.
Clinopyroxene chemical profile and diffusion modeling. (A) SEM-BSE image of the analyzed clinopyroxene. The red line with numbers marks the analyzed chemical profile reported in C. (B) SEM-BSE high resolution image showing the area (light blue area) where the grey scale profile for the diffusion profile has been extracted using the *greyvalues* Matlab script of the NIDIS model from Petrone *et al.*[5]. The red dot on the light blue area marks the initial point of the profile. (C) Variation of Ca (blue dots +line), Al (grey dots + line) (a.p.f.u.) and Mg# (orange dots + line) *vs* distance (μm) along the chemical profile shown in (A). (D) Grey values diffusion profile (blue dots with error bar) *vs* distance (μm) extracted along the light blue area in (B). The red line is the fit of the diffusion modeling calculated using the *createfit* Matlab script of the NIDIS model from Petrone *et al.*[5]. The goodness of the fitting is reported as r^2 and Δt is the calculated timescales (in days or years). The grey values are used as proxy of Mg# in clinopyroxene following Petrone *et al.*[5]. The temperature at which the diffusion has been calculated is reported in Table 1 for each clinopyroxene. See Method and Materials for further details.

Paroxysm 28 August 2019, Sample P47-1, lp – Px17 – Subgroup 1B

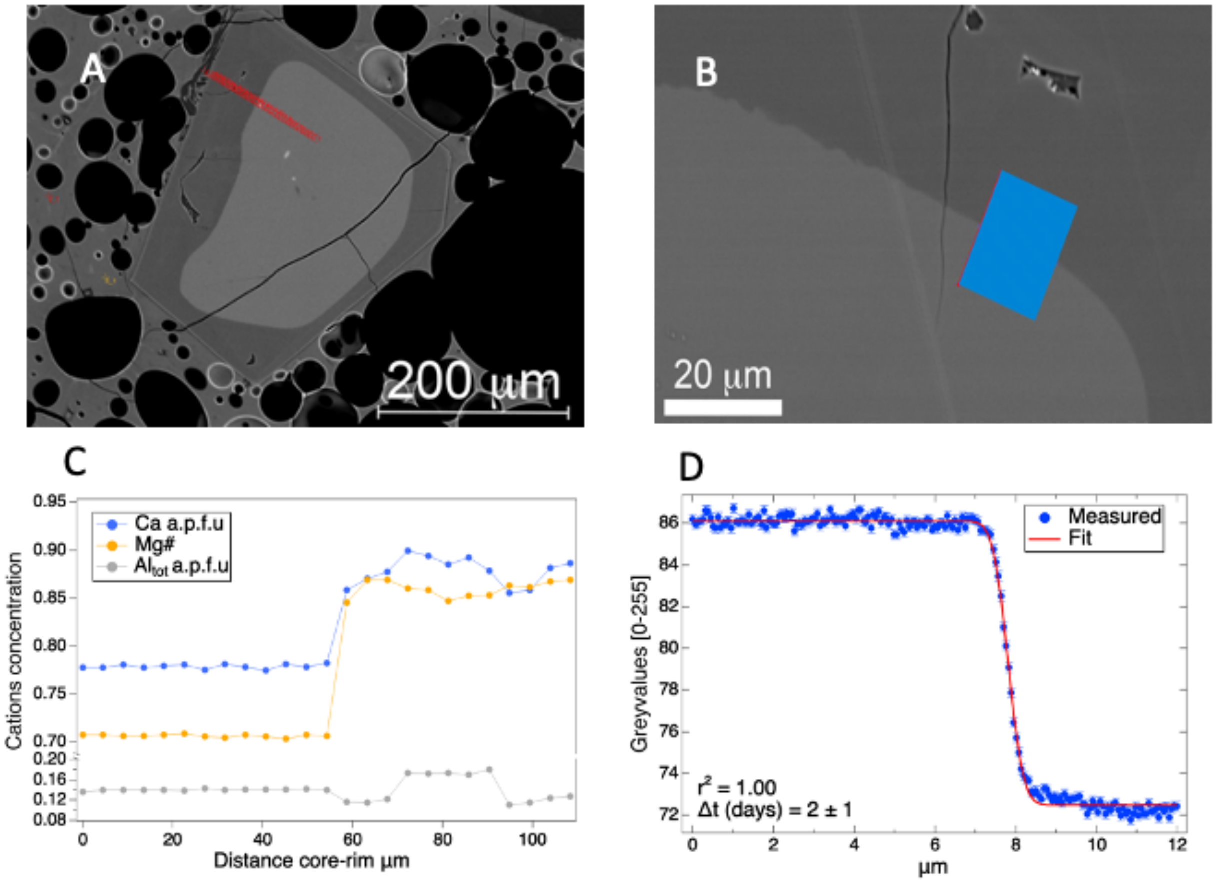

Supplementary Fig. 50.
Clinopyroxene chemical profile and diffusion modeling. (A) SEM-BSE image of the analyzed clinopyroxene. The red line with numbers marks the analyzed chemical profile reported in C. (B) SEM-BSE high resolution image showing the area (light blue area) where the grey scale profile for the diffusion profile has been extracted using the *greyvalues* Matlab script of the NIDIS model from Petrone *et al.*[5]. The red dot on the light blue area marks the initial point of the profile. (C) Variation of Ca (blue dots +line), Al (grey dots + line) (a.p.f.u.) and Mg# (orange dots + line) *vs* distance (μm) along the chemical profile shown in (A). (D) Grey values diffusion profile (blue dots with error bar) *vs* distance (μm) extracted along the light blue area in (B). The red line is the fit of the diffusion modeling calculated using the *createfit* Matlab script of the NIDIS model from Petrone *et al.*[5]. The goodness of the fitting is reported as r^2 and Δt is the calculated timescales (in days or years). The grey values are used as proxy of Mg# in clinopyroxene following Petrone *et al.*[5]. The temperature at which the diffusion has been calculated is reported in Table 1 for each clinopyroxene. See Method and Materials for further details.

Paroxysm 28 August 2019, Sample P47-1, lp – Px18 – Subgroup 1B

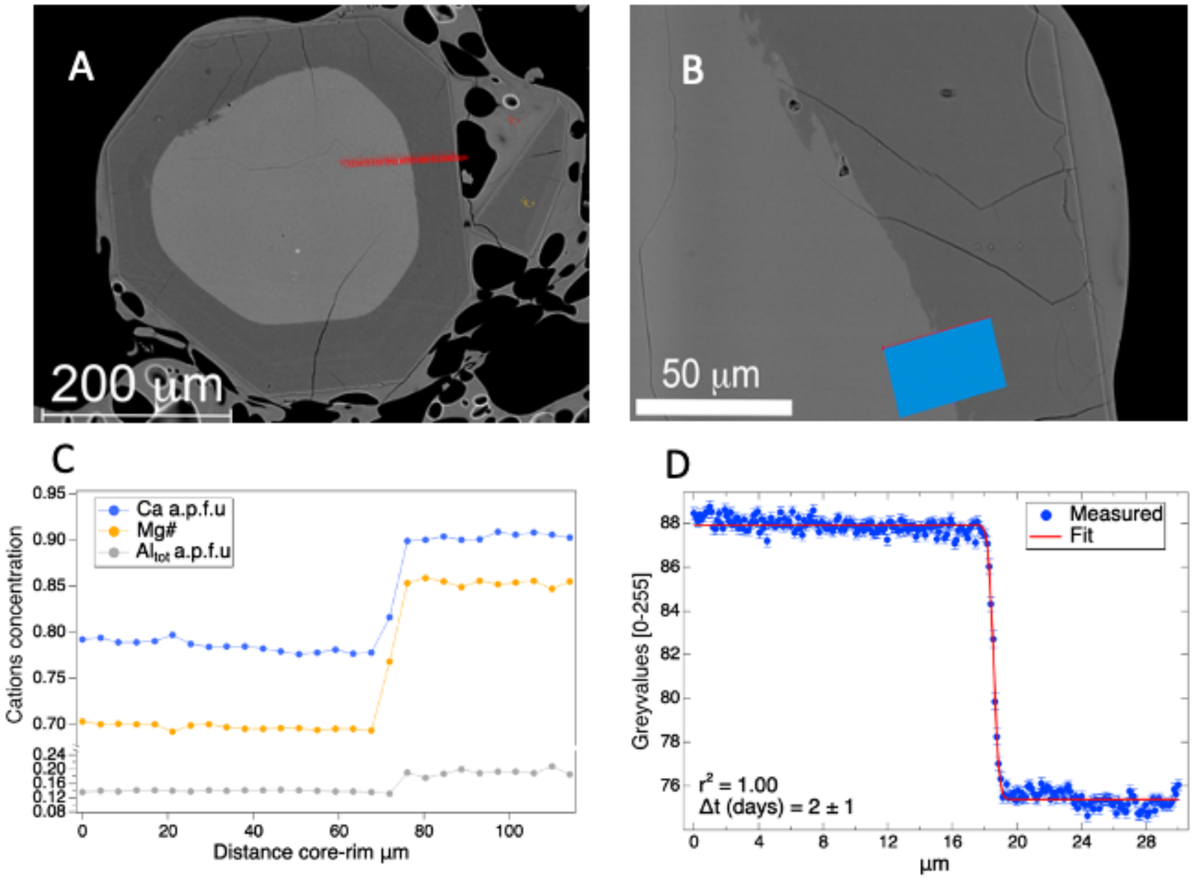

Supplementary Fig. 51.
Clinopyroxene chemical profile and diffusion modeling. (A) SEM-BSE image of the analyzed clinopyroxene. The red line with numbers marks the analyzed chemical profile reported in C. (B) SEM-BSE high resolution image showing the area (light blue area) where the grey scale profile for the diffusion profile has been extracted using the *greyvalues* Matlab script of the NIDIS model from Petrone *et al.*[5]. The red dot on the light blue area marks the initial point of the profile. (C) Variation of Ca (blue dots +line), Al (grey dots + line) (a.p.f.u.) and Mg# (orange dots + line) *vs* distance (µm) along the chemical profile shown in (A). (D) Grey values diffusion profile (blue dots with error bar) *vs* distance (µm) extracted along the light blue area in (B). The red line is the fit of the diffusion modeling calculated using the *createfit* Matlab script of the NIDIS model from Petrone *et al.*[5]. The goodness of the fitting is reported as r^2 and Δt is the calculated timescales (in days or years). The grey values are used as proxy of Mg# in clinopyroxene following Petrone *et al.*[5]. The temperature at which the diffusion has been calculated is reported in Table 1 for each clinopyroxene. See Method and Materials for further details.

Paroxysm 28 August 2019, Sample P47-1, lp – Px19 – Subgroup 1B

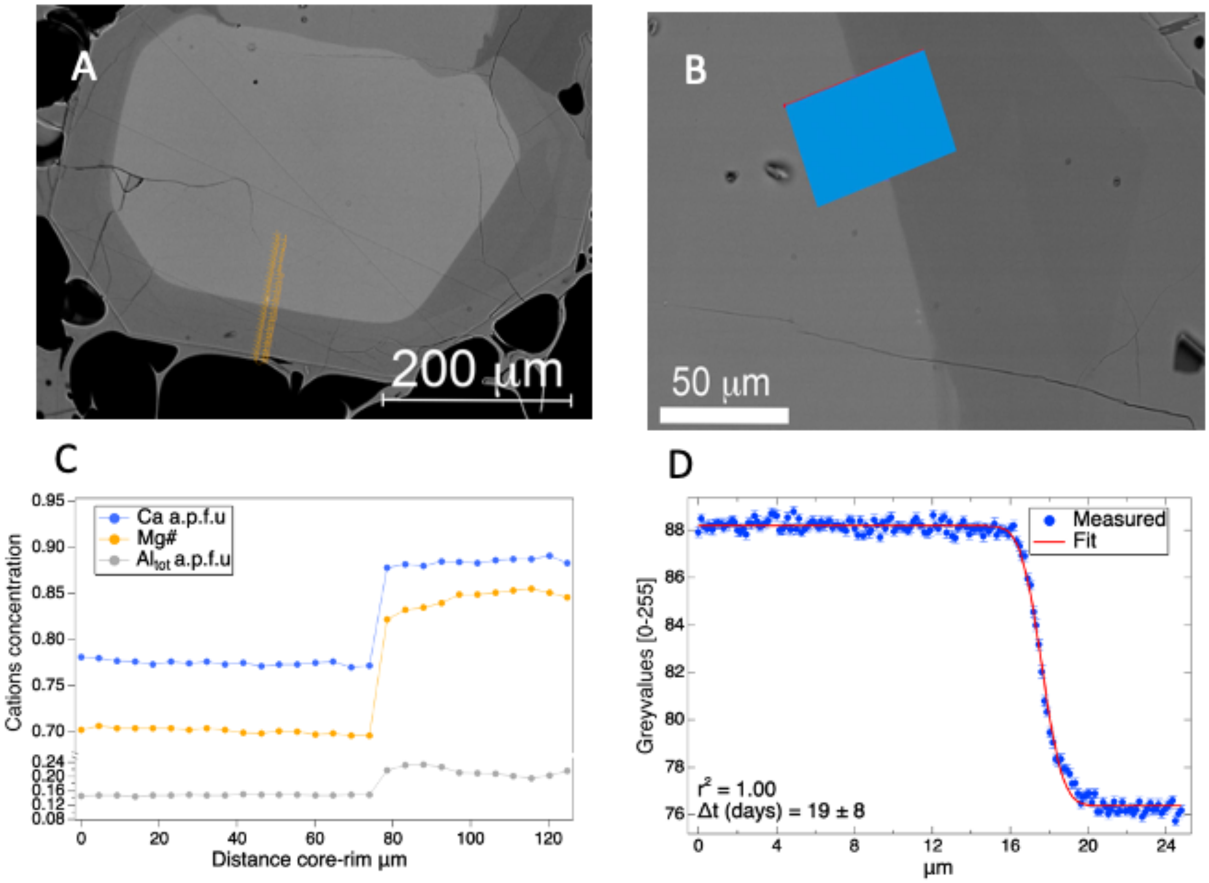

Supplementary Fig. 52.

Clinopyroxene chemical profile and diffusion modeling. (A) SEM-BSE image of the analyzed clinopyroxene. The yellow line with numbers marks the analyzed chemical profile reported in C. (B) SEM-BSE high resolution image showing the area (light blue area) where the grey scale profile for the diffusion profile has been extracted using the *greyvalues* Matlab script of the NIDIS model from Petrone *et al.*[5]. The red dot on the light blue area marks the initial point of the profile. (C) Variation of Ca (blue dots +line), Al (grey dots + line) (a.p.f.u.) and Mg# (orange dots + line) *vs* distance (µm) along the chemical profile shown in (A). (D) Grey values diffusion profile (blue dots with error bar) *vs* distance (µm) extracted along the light blue area in (B). The red line is the fit of the diffusion modeling calculated using the *createfit* Matlab script of the NIDIS model from Petrone *et al.*[5]. The goodness of the fitting is reported as r^2 and Δt is the calculated timescales (in days or years). The grey values are used as proxy of Mg# in clinopyroxene following Petrone *et al.*[5]. The temperature at which the diffusion has been calculated is reported in Table 1 for each clinopyroxene. See Method and Materials for further details.

Paroxysm 28 August 2019, Sample P47-1, lp – Px20 – Subgroup 1B

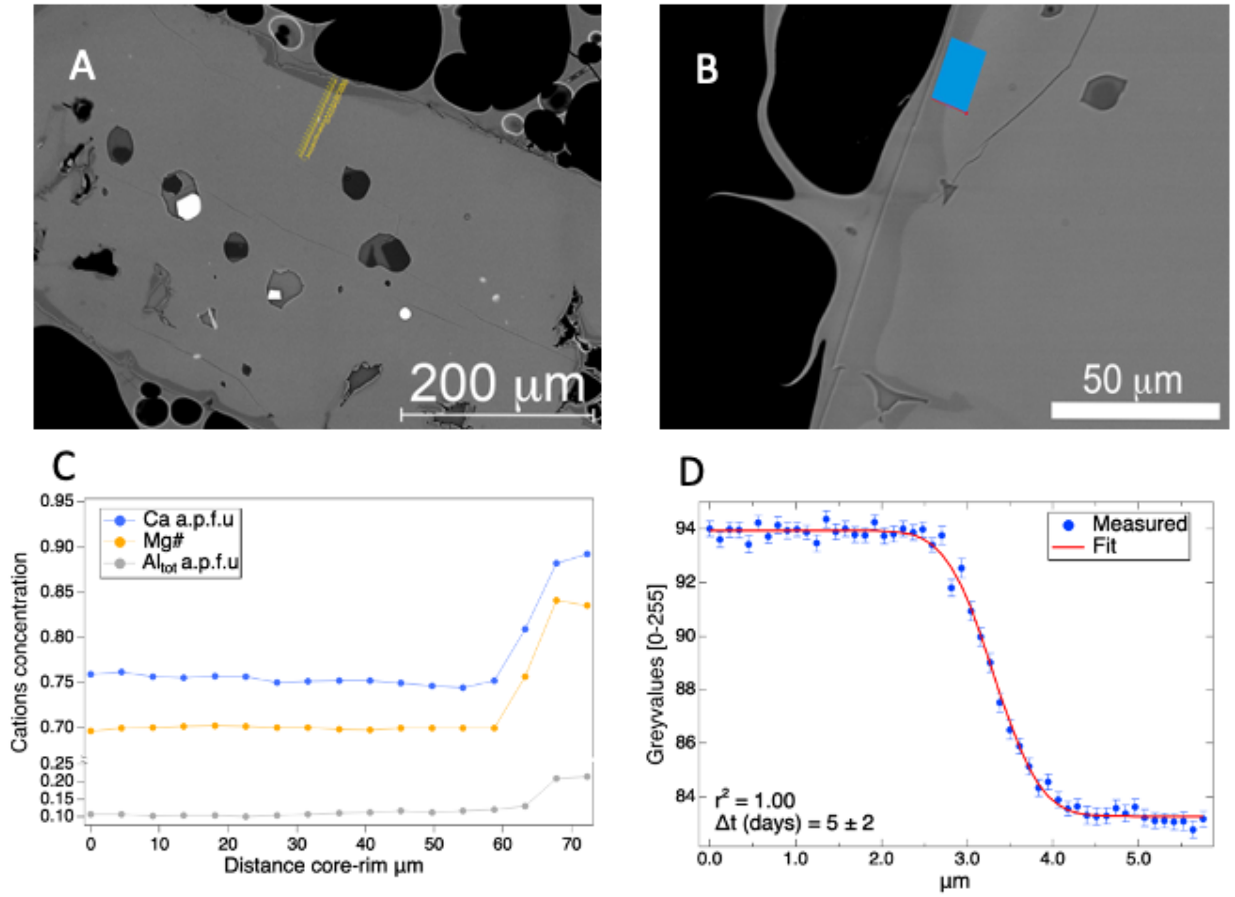

Supplementary Fig. 53.
Clinopyroxene chemical profile and diffusion modeling. (A) SEM-BSE image of the analyzed clinopyroxene. The yellow line with numbers marks the analyzed chemical profile reported in C. (B) SEM-BSE high resolution image showing the area (light blue area) where the grey scale profile for the diffusion profile has been extracted using the *greyvalues* Matlab script of the NIDIS model from Petrone *et al.*[5]. The red dot on the light blue area marks the initial point of the profile. (C) Variation of Ca (blue dots +line), Al (grey dots + line) (a.p.f.u.) and Mg# (orange dots + line) *vs* distance (µm) along the chemical profile shown in (A). (D) Grey values diffusion profile (blue dots with error bar) *vs* distance (µm) extracted along the light blue area in (B). The red line is the fit of the diffusion modeling calculated using the *createfit* Matlab script of the NIDIS model from Petrone *et al.*[5]. The goodness of the fitting is reported as r^2 and Δt is the calculated timescales (in days or years). The grey values are used as proxy of Mg# in clinopyroxene following Petrone *et al.*[5]. The temperature at which the diffusion has been calculated is reported in Table 1 for each clinopyroxene. See Method and Materials for further details.

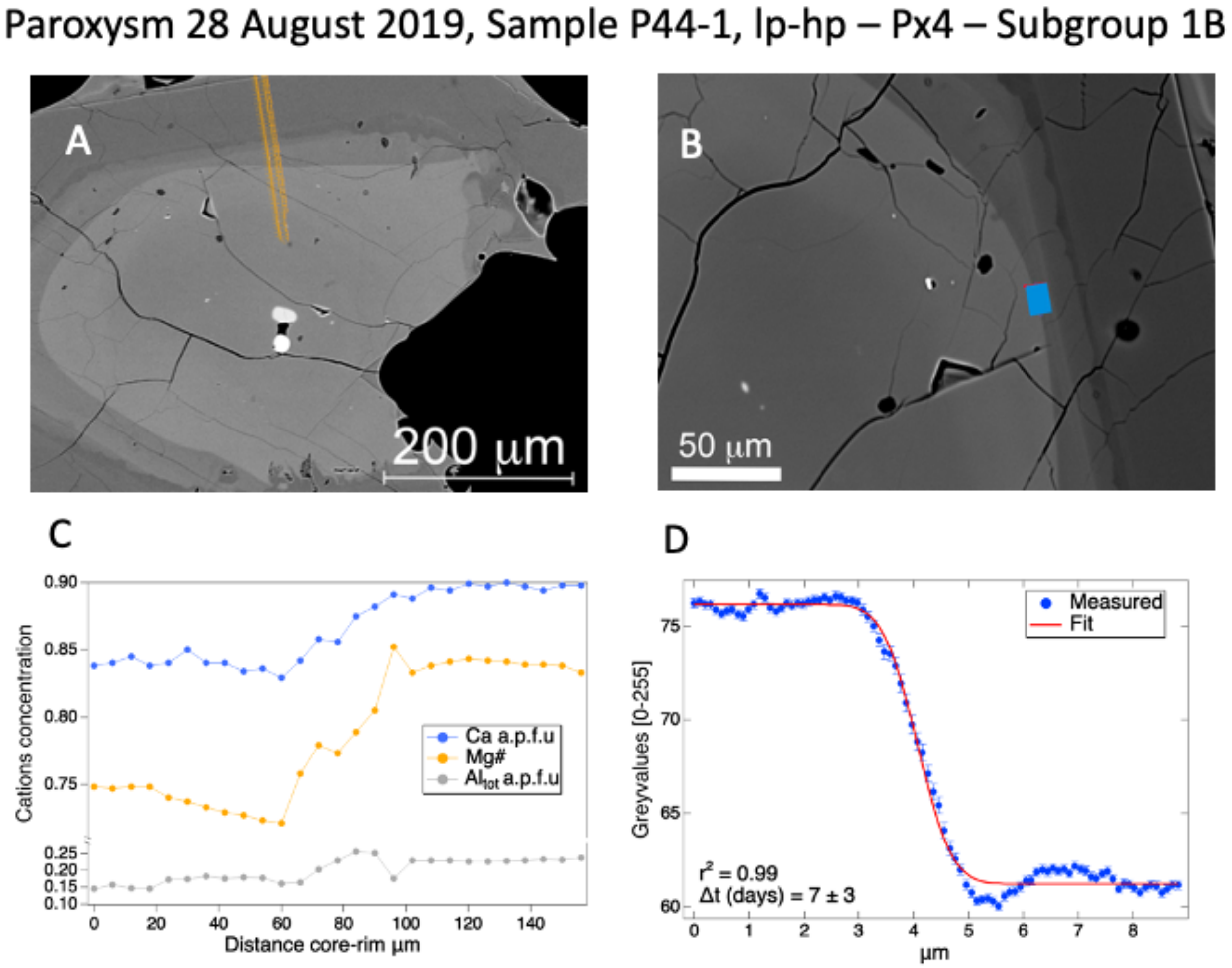

Supplementary Fig. 54.

Clinopyroxene chemical profile and diffusion modeling. (A) SEM-BSE image of the analyzed clinopyroxene. The yellow line with numbers marks the analyzed chemical profile reported in C. (B) SEM-BSE high resolution image showing the area (light blue area) where the grey scale profile for the diffusion profile has been extracted using the *greyvalues* Matlab script of the NIDIS model from Petrone *et al.*[5]. The red dot on the light blue area marks the initial point of the profile. (C) Variation of Ca (blue dots +line), Al (grey dots + line) (a.p.f.u.) and Mg# (orange dots + line) *vs* distance (μm) along the chemical profile shown in (A). (D) Grey values diffusion profile (blue dots with error bar) *vs* distance (μm) extracted along the light blue area in (B). The red line is the fit of the diffusion modeling calculated using the *createfit* Matlab script of the NIDIS model from Petrone *et al.*[5]. The goodness of the fitting is reported as r^2 and Δt is the calculated timescales (in days or years). The grey values are used as proxy of Mg# in clinopyroxene following Petrone *et al.*[5]. The temperature at which the diffusion has been calculated is reported in Table 1 for each clinopyroxene. See Method and Materials for further details.

Paroxysm 28 August 2019, Sample P44-1, lp-hp – Px8 – Subgroup 1B

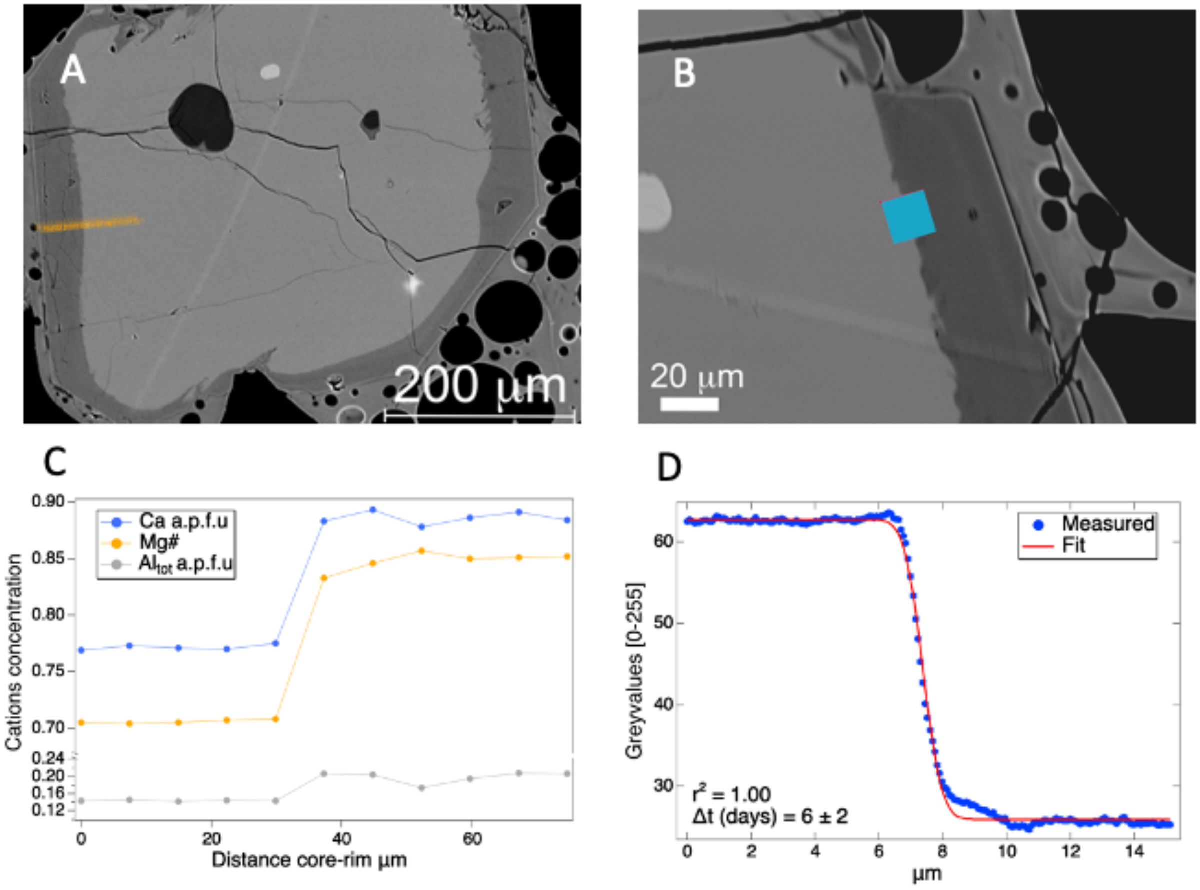

Supplementary Fig. 55.
Clinopyroxene chemical profile and diffusion modeling. (A) SEM-BSE image of the analyzed clinopyroxene. The yellow line with numbers marks the analyzed chemical profile reported in C. (B) SEM-BSE high resolution image showing the area (light blue area) where the grey scale profile for the diffusion profile has been extracted using the *greyvalues* Matlab script of the NIDIS model from Petrone *et al.*[5]. The red dot on the light blue area marks the initial point of the profile. (C) Variation of Ca (blue dots +line), Al (grey dots + line) (a.p.f.u.) and Mg# (orange dots + line) *vs* distance (μm) along the chemical profile shown in (A). (D) Grey values diffusion profile (blue dots with error bar) *vs* distance (μm) extracted along the light blue area in (B). The red line is the fit of the diffusion modeling calculated using the *createfit* Matlab script of the NIDIS model from Petrone *et al.*[5]. The goodness of the fitting is reported as r^2 and Δt is the calculated timescales (in days or years). The grey values are used as proxy of Mg# in clinopyroxene following Petrone *et al.*[5]. The temperature at which the diffusion has been calculated is reported in Table 1 for each clinopyroxene. See Method and Materials for further details.

Major 25 June 2019, Sample 25062019, hp – Px35 – Subgroup 1C

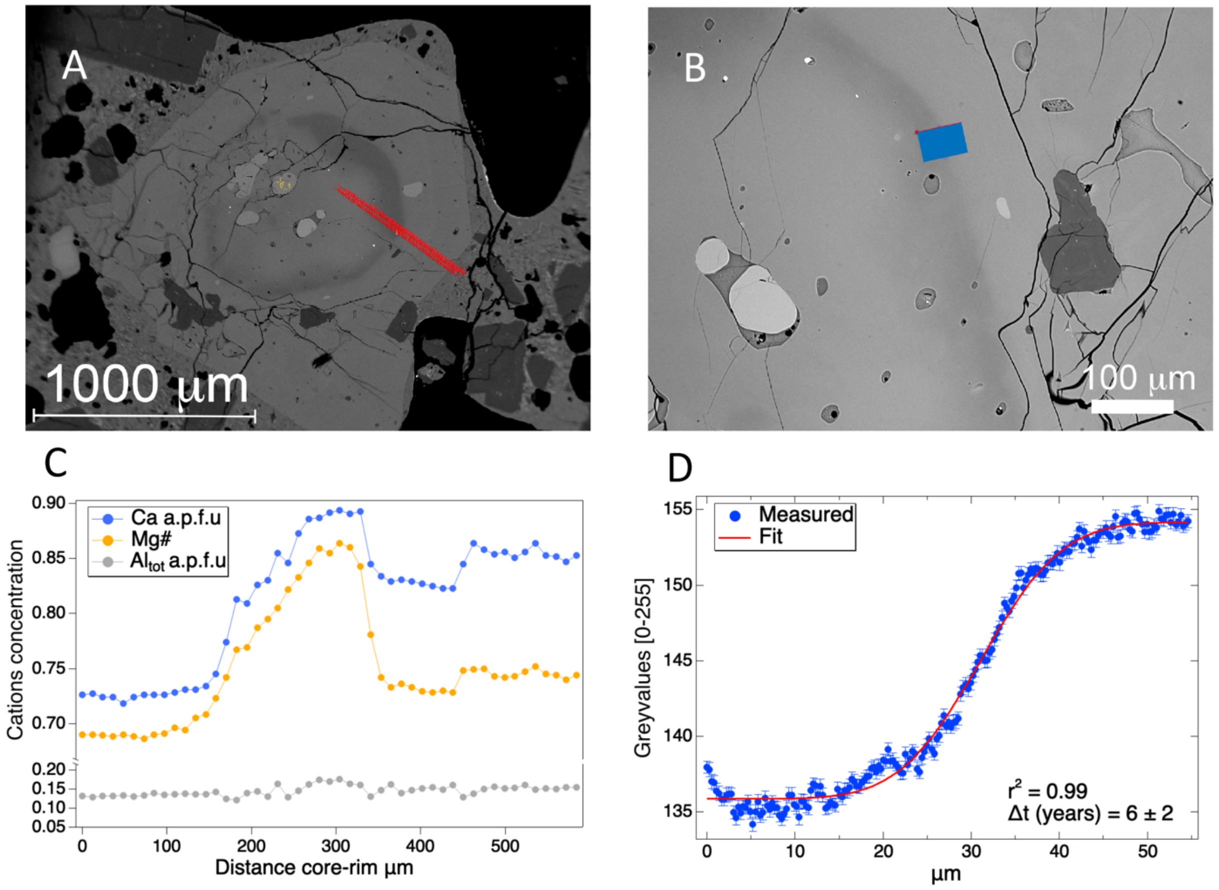

Supplementary Fig. 56.

Clinopyroxene chemical profile and diffusion modeling. (A) SEM-BSE image of the analyzed clinopyroxene. The red line with numbers marks the analyzed chemical profile reported in C. (B) SEM-BSE high resolution image showing the area (light blue area) where the grey scale profile for the diffusion profile has been extracted using the *greyvalues* Matlab script of the NIDIS model from Petrone *et al.*[5]. The red dot on the light blue area marks the initial point of the profile. (C) Variation of Ca (blue dots +line), Al (grey dots + line) (a.p.f.u.) and Mg# (orange dots + line) *vs* distance (μm) along the chemical profile shown in (A). (D) Grey values diffusion profile (blue dots with error bar) *vs* distance (μm) extracted along the light blue area in (B). The red line is the fit of the diffusion modeling calculated using the *createfit* Matlab script of the NIDIS model from Petrone *et al.*[5]. The goodness of the fitting is reported as r^2 and Δt is the calculated timescales (in days or years). The grey values are used as proxy of Mg# in clinopyroxene following Petrone *et al.*[5]. The temperature at which the diffusion has been calculated is reported in Table 1 for each clinopyroxene. See Method and Materials for further details.

Paroxysm 3 July 2019, Sample P01-05, lp – Px9 – Subgroup 1C

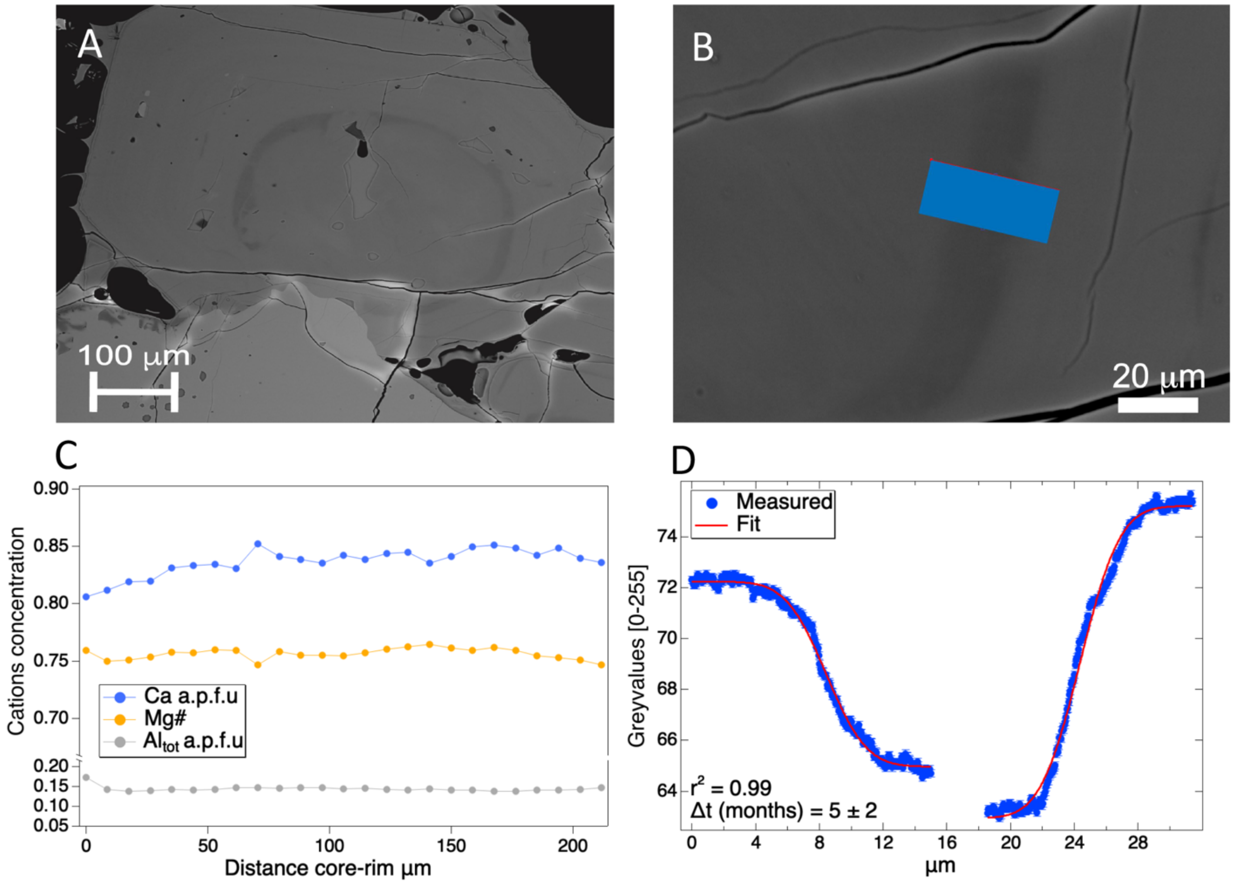

Supplementary Fig. 57.
Clinopyroxene chemical profile and diffusion modeling. (A) SEM-BSE image of the analyzed clinopyroxene. The red line with numbers marks the analyzed chemical profile reported in C. (B) SEM-BSE high resolution image showing the area (light blue area) where the grey scale profile for the diffusion profile has been extracted using the *greyvalues* Matlab script of the NIDIS model from Petrone *et al.*[5]. The red dot on the light blue area marks the initial point of the profile. (C) Variation of Ca (blue dots +line), Al (grey dots + line) (a.p.f.u.) and Mg# (orange dots + line) *vs* distance (μm) along the chemical profile shown in (A). (D) Grey values diffusion profile (blue dots with error bar) *vs* distance (μm) extracted along the light blue area in (B). The red line is the fit of the diffusion modeling calculated using the *createfit* Matlab script of the NIDIS model from Petrone *et al.*[5]. The goodness of the fitting is reported as r^2 and Δt is the calculated timescales (in days or years). The grey values are used as proxy of Mg# in clinopyroxene following Petrone *et al.*[5]. The temperature at which the diffusion has been calculated is reported in Table 1 for each clinopyroxene. See Method and Materials for further details.

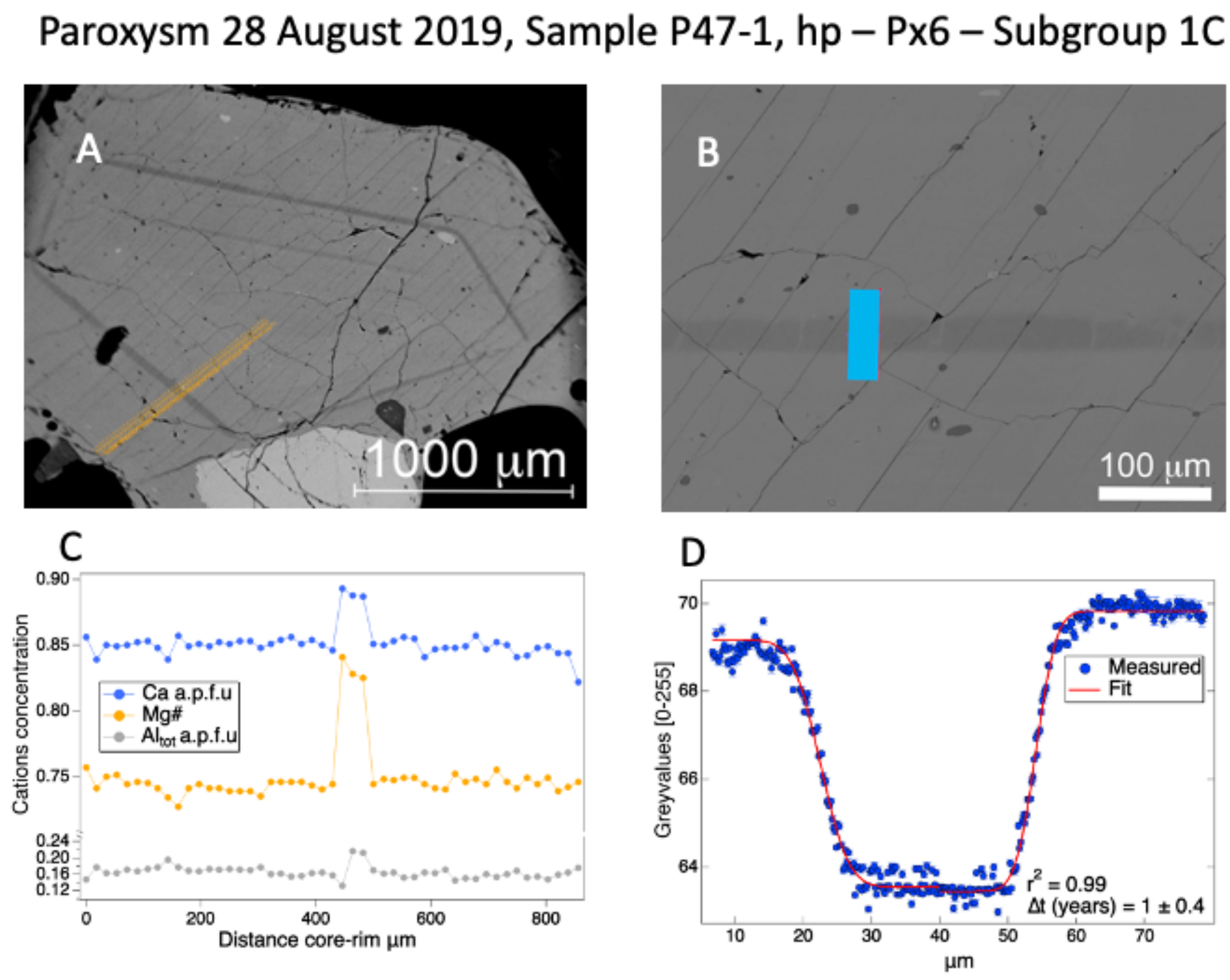

Supplementary Fig. 58.
Clinopyroxene chemical profile and diffusion modeling. (A) SEM-BSE image of the analyzed clinopyroxene. The yellow line with numbers marks the analyzed chemical profile reported in C. (B) SEM-BSE high resolution image showing the area (light blue area) where the grey scale profile for the diffusion profile has been extracted using the *greyvalues* Matlab script of the NIDIS model from Petrone *et al.*[5]. The red dot on the light blue area marks the initial point of the profile. (C) Variation of Ca (blue dots +line), Al (grey dots + line) (a.p.f.u.) and Mg# (orange dots + line) *vs* distance (μm) along the chemical profile shown in (A). (D) Grey values diffusion profile (blue dots with error bar) *vs* distance (μm) extracted along the light blue area in (B). The red line is the fit of the diffusion modeling calculated using the *createfit* Matlab script of the NIDIS model from Petrone *et al.*[5]. The goodness of the fitting is reported as r^2 and Δt is the calculated timescales (in days or years). The grey values are used as proxy of Mg# in clinopyroxene following Petrone *et al.*[5]. The temperature at which the diffusion has been calculated is reported in Table 1 for each clinopyroxene. See Method and Materials for further details.

Paroxysm 3 July 2019, Sample P01-05, lp – Px2 – Subgroup 2A

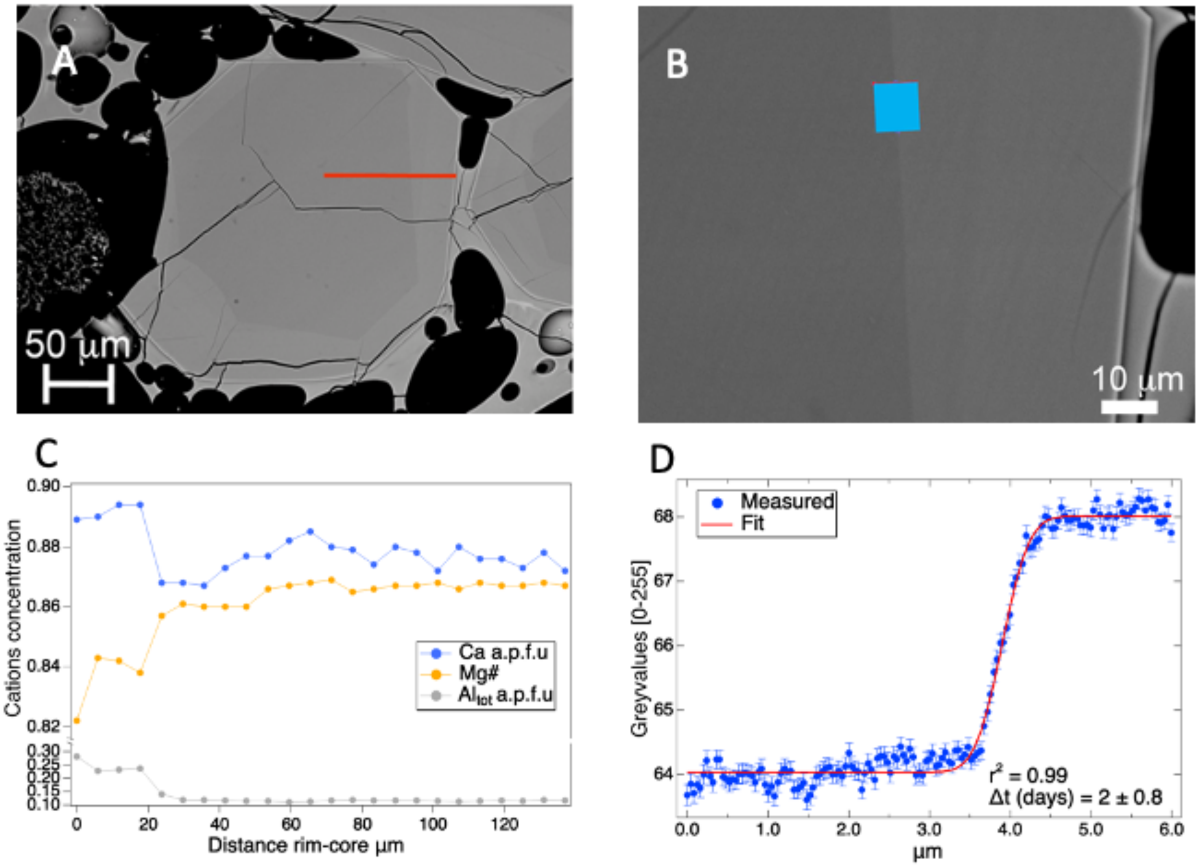

Supplementary Fig. 59.
Clinopyroxene chemical profile and diffusion modeling. (A) SEM-BSE image of the analyzed clinopyroxene. The red line marks the analyzed chemical profile reported in C. (B) SEM-BSE high resolution image showing the area (light blue area) where the grey scale profile for the diffusion profile has been extracted using the *greyvalues* Matlab script of the NIDIS model from Petrone *et al.*[5]. The red dot on the light blue area marks the initial point of the profile. (C) Variation of Ca (blue dots +line), Al (grey dots + line) (a.p.f.u.) and Mg# (orange dots + line) *vs* distance (µm) along the chemical profile shown in (A). (D) Grey values diffusion profile (blue dots with error bar) *vs* distance (µm) extracted along the light blue area in (B). The red line is the fit of the diffusion modeling calculated using the *createfit* Matlab script of the NIDIS model from Petrone *et al.*[5]. The goodness of the fitting is reported as r^2 and Δt is the calculated timescales (in days or years). The grey values are used as proxy of Mg# in clinopyroxene following Petrone *et al.*[5]. The temperature at which the diffusion has been calculated is reported in Table 1 for each clinopyroxene. See Method and Materials for further details.

Paroxysm 3 July 2019, Sample P21-1, lp – Px5 – Subgroup 2A

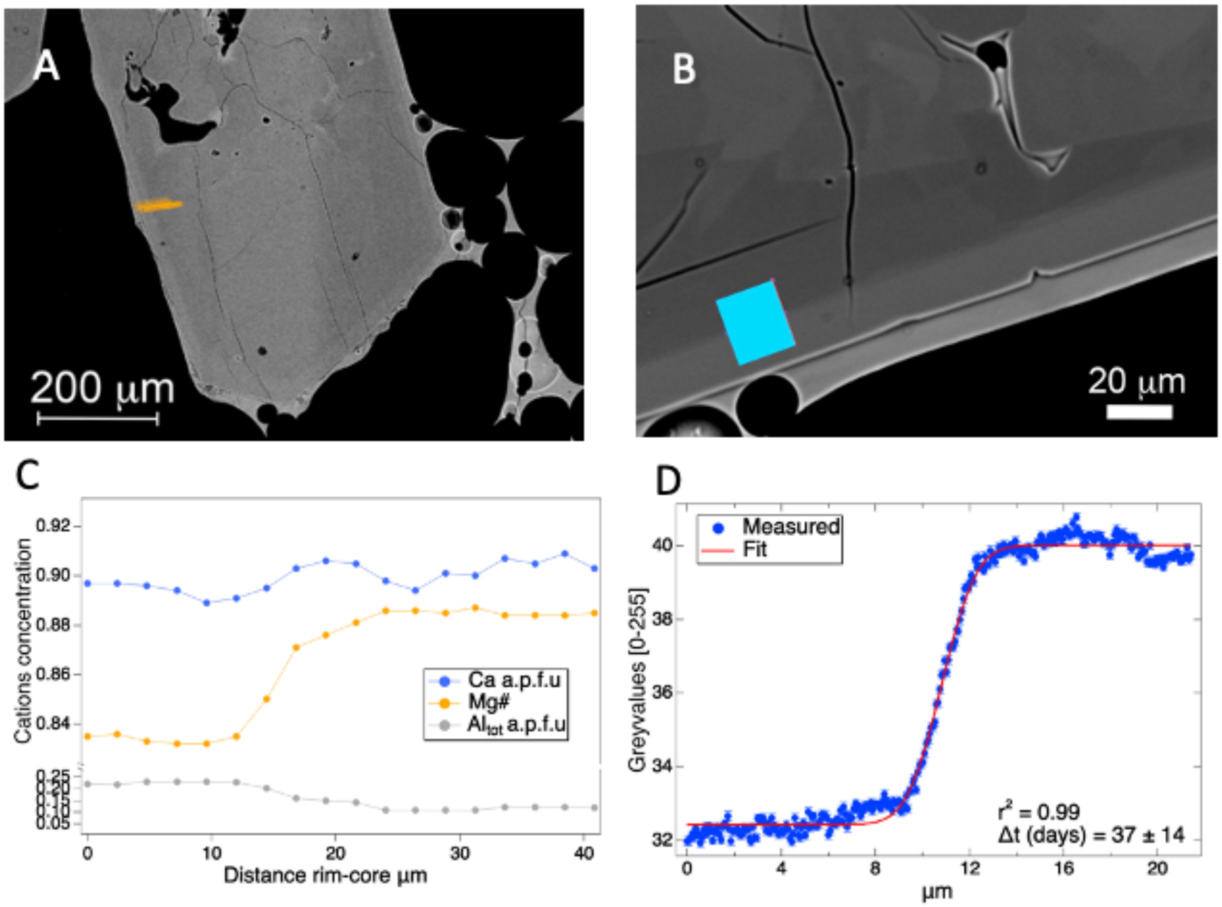

Supplementary Fig. 60.
Clinopyroxene chemical profile and diffusion modeling. (A) SEM-BSE image of the analyzed clinopyroxene. The yellow line with numbers marks the analyzed chemical profile reported in C. (B) SEM-BSE high resolution image showing the area (light blue area) where the grey scale profile for the diffusion profile has been extracted using the *greyvalues* Matlab script of the NIDIS model from Petrone *et al.*[5]. The red dot on the light blue area marks the initial point of the profile. (C) Variation of Ca (blue dots +line), Al (grey dots + line) (a.p.f.u.) and Mg# (orange dots + line) *vs* distance (µm) along the chemical profile shown in (A). (D) Grey values diffusion profile (blue dots with error bar) *vs* distance (µm) extracted along the light blue area in (B). The red line is the fit of the diffusion modeling calculated using the *createfit* Matlab script of the NIDIS model from Petrone *et al.*[5]. The goodness of the fitting is reported as r^2 and Δt is the calculated timescales (in days or years). The grey values are used as proxy of Mg# in clinopyroxene following Petrone *et al.*[5]. The temperature at which the diffusion has been calculated is reported in Table 1 for each clinopyroxene. See Method and Materials for further details.

Paroxysm 28 August 2019, Sample P44-1, lp – Px6 – Subgroup 2A

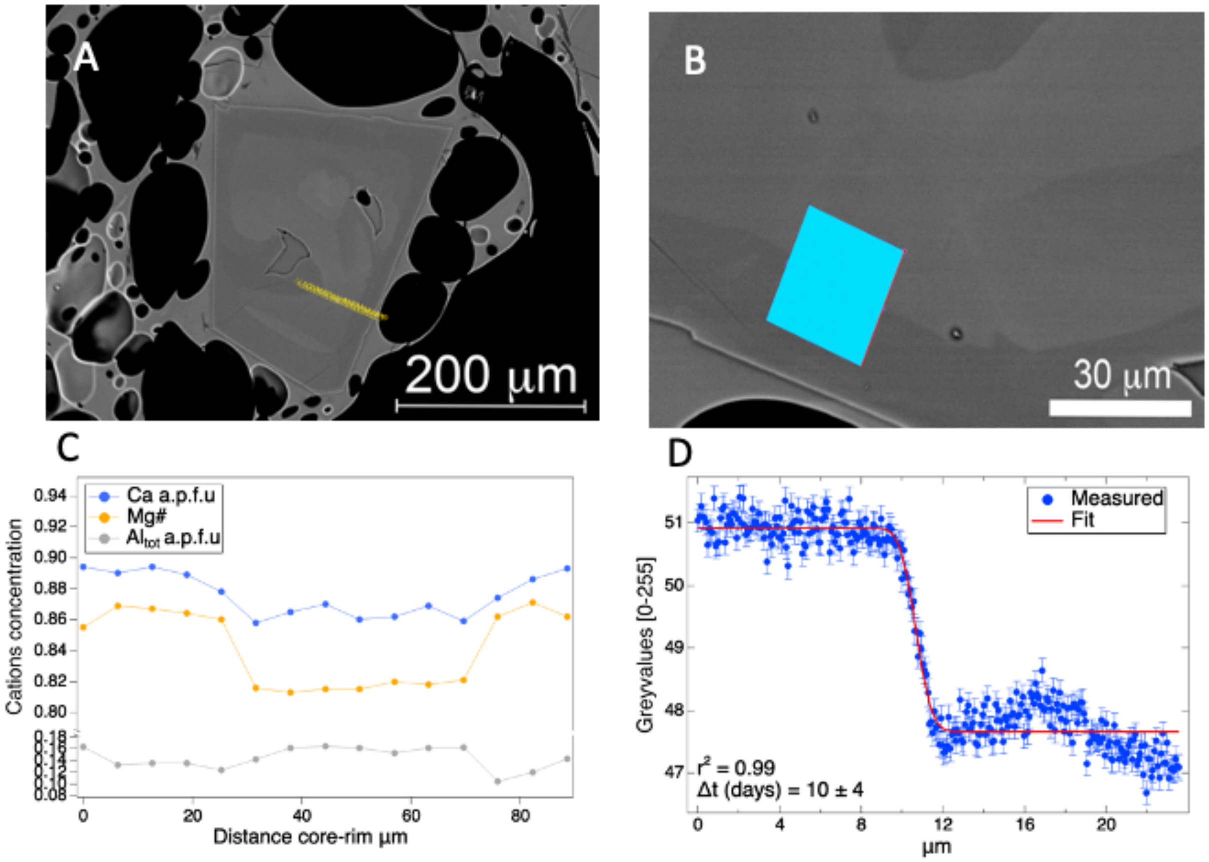

Supplementary Fig. 61.
Clinopyroxene chemical profile and diffusion modeling. (A) SEM-BSE image of the analyzed clinopyroxene. The yellow line with numbers marks the analyzed chemical profile reported in C. (B) SEM-BSE high resolution image showing the area (light blue area) where the grey scale profile for the diffusion profile has been extracted using the *greyvalues* Matlab script of the NIDIS model from Petrone *et al.*[5]. The red dot on the light blue area marks the initial point of the profile. (C) Variation of Ca (blue dots +line), Al (grey dots + line) (a.p.f.u.) and Mg# (orange dots + line) *vs* distance (μm) along the chemical profile shown in (A). (D) Grey values diffusion profile (blue dots with error bar) *vs* distance (μm) extracted along the light blue area in (B). The red line is the fit of the diffusion modeling calculated using the *createfit* Matlab script of the NIDIS model from Petrone *et al.*[5]. The goodness of the fitting is reported as r^2 and Δt is the calculated timescales (in days or years). The grey values are used as proxy of Mg# in clinopyroxene following Petrone *et al.*[5]. The temperature at which the diffusion has been calculated is reported in Table 1 for each clinopyroxene. See Method and Materials for further details.

Paroxysm 28 August 2019, Sample P44-1, lp-hp – Px6 – Subgroup 2A

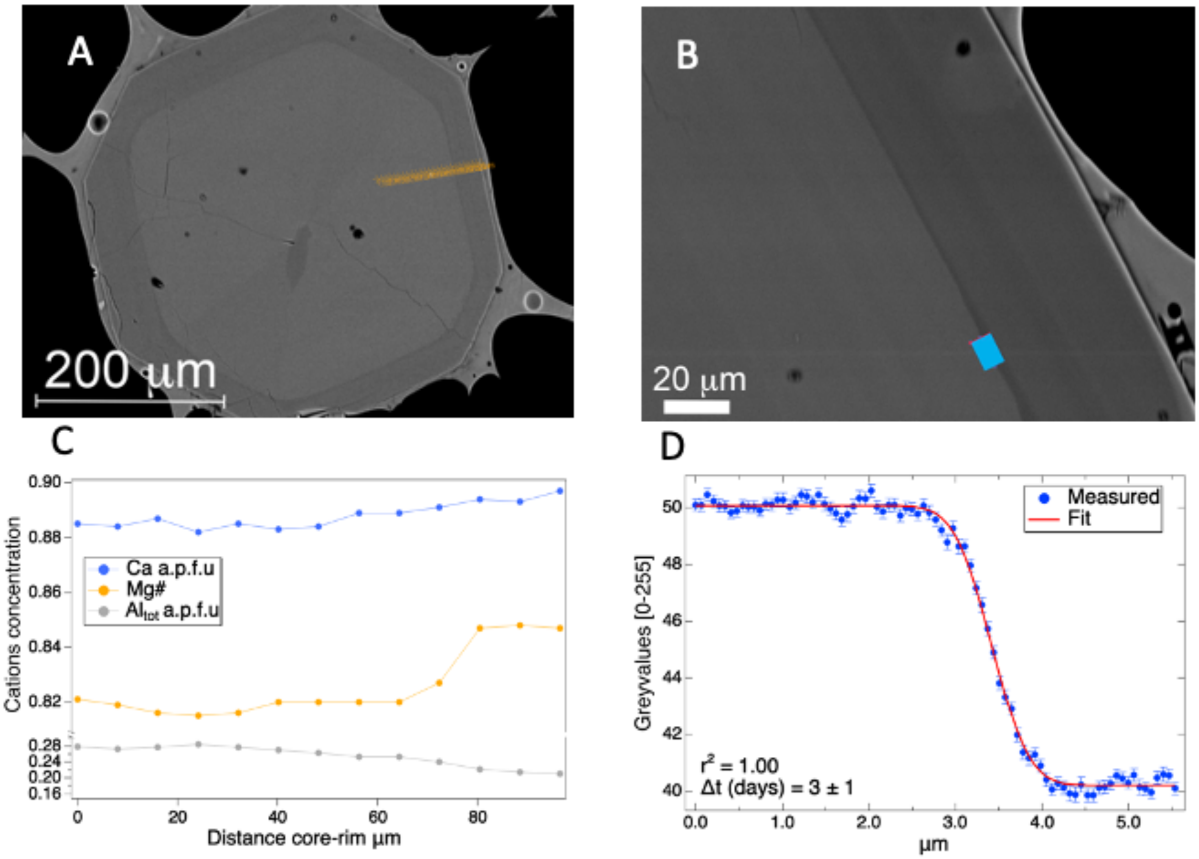

Supplementary Fig. 62.

Clinopyroxene chemical profile and diffusion modeling. (A) SEM-BSE image of the analyzed clinopyroxene. The yellow line with numbers marks the analyzed chemical profile reported in C. (B) SEM-BSE high resolution image showing the area (light blue area) where the grey scale profile for the diffusion profile has been extracted using the *greyvalues* Matlab script of the NIDIS model from Petrone *et al.*[5]. The red dot on the light blue area marks the initial point of the profile. (C) Variation of Ca (blue dots +line), Al (grey dots + line) (a.p.f.u.) and Mg# (orange dots + line) *vs* distance (μm) along the chemical profile shown in (A). (D) Grey values diffusion profile (blue dots with error bar) *vs* distance (μm) extracted along the light blue area in (B). The red line is the fit of the diffusion modeling calculated using the *createfit* Matlab script of the NIDIS model from Petrone *et al.*[5]. The goodness of the fitting is reported as r^2 and Δt is the calculated timescales (in days or years). The grey values are used as proxy of Mg# in clinopyroxene following Petrone *et al.*[5]. The temperature at which the diffusion has been calculated is reported in Table 1 for each clinopyroxene. See Method and Materials for further details.

Paroxysm 3 July 2019, Sample P30-07, hp – Px31 – Subgroup 2B

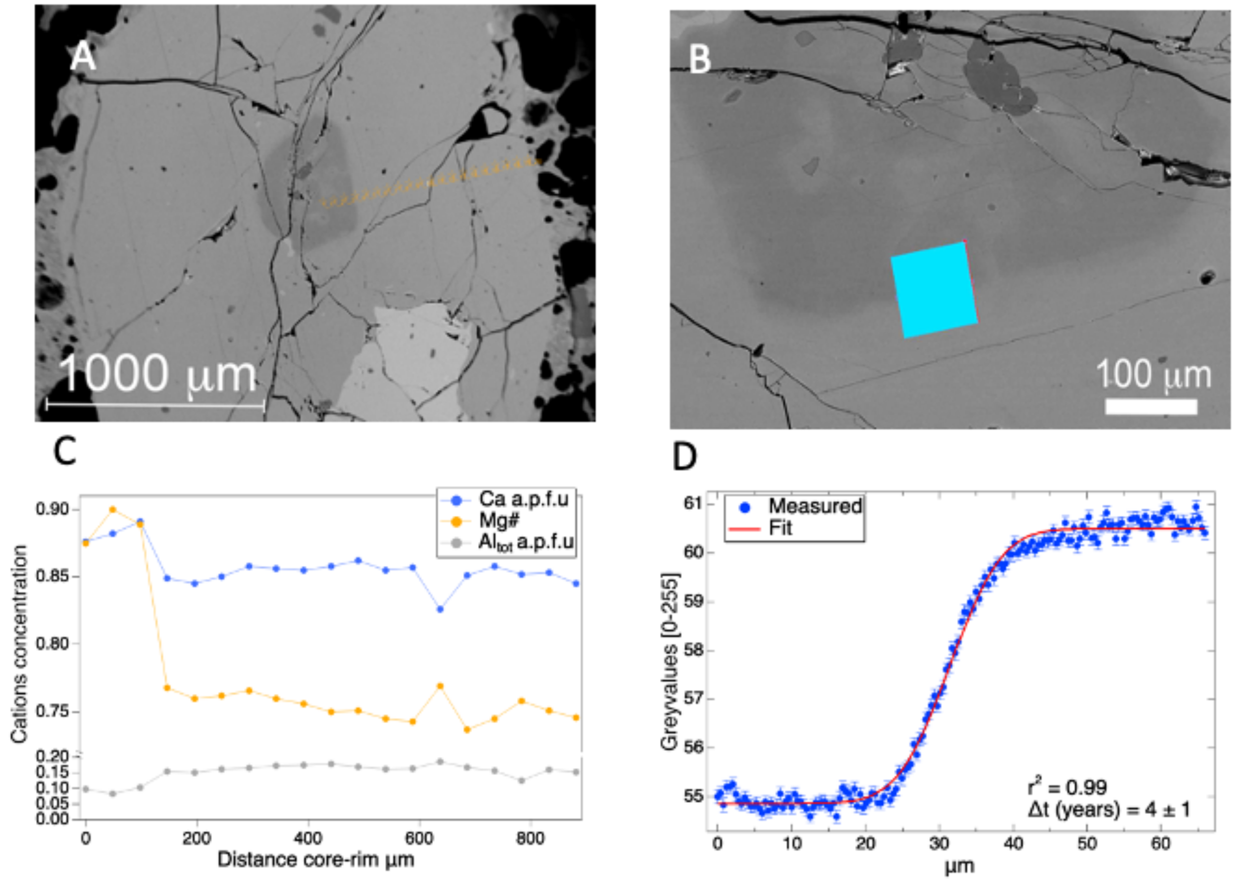

Supplementary Fig. 63.
Clinopyroxene chemical profile and diffusion modeling. (A) SEM-BSE image of the analyzed clinopyroxene. The yellow line with numbers marks the analyzed chemical profile reported in C. (B) SEM-BSE high resolution image showing the area (light blue area) where the grey scale profile for the diffusion profile has been extracted using the *greyvalues* Matlab script of the NIDIS model from Petrone *et al.*[5]. The red dot on the light blue area marks the initial point of the profile. (C) Variation of Ca (blue dots +line), Al (grey dots + line) (a.p.f.u.) and Mg# (orange dots + line) *vs* distance (µm) along the chemical profile shown in (A). (D) Grey values diffusion profile (blue dots with error bar) *vs* distance (µm) extracted along the light blue area in (B). The red line is the fit of the diffusion modeling calculated using the *createfit* Matlab script of the NIDIS model from Petrone *et al.*[5]. The goodness of the fitting is reported as r^2 and Δt is the calculated timescales (in days or years). The grey values are used as proxy of Mg# in clinopyroxene following Petrone *et al.*[5]. The temperature at which the diffusion has been calculated is reported in Table 1 for each clinopyroxene. See Method and Materials for further details.

Paroxysm 28 August 2019, Sample P47-1, hp – Px12 – Subgroup 2B

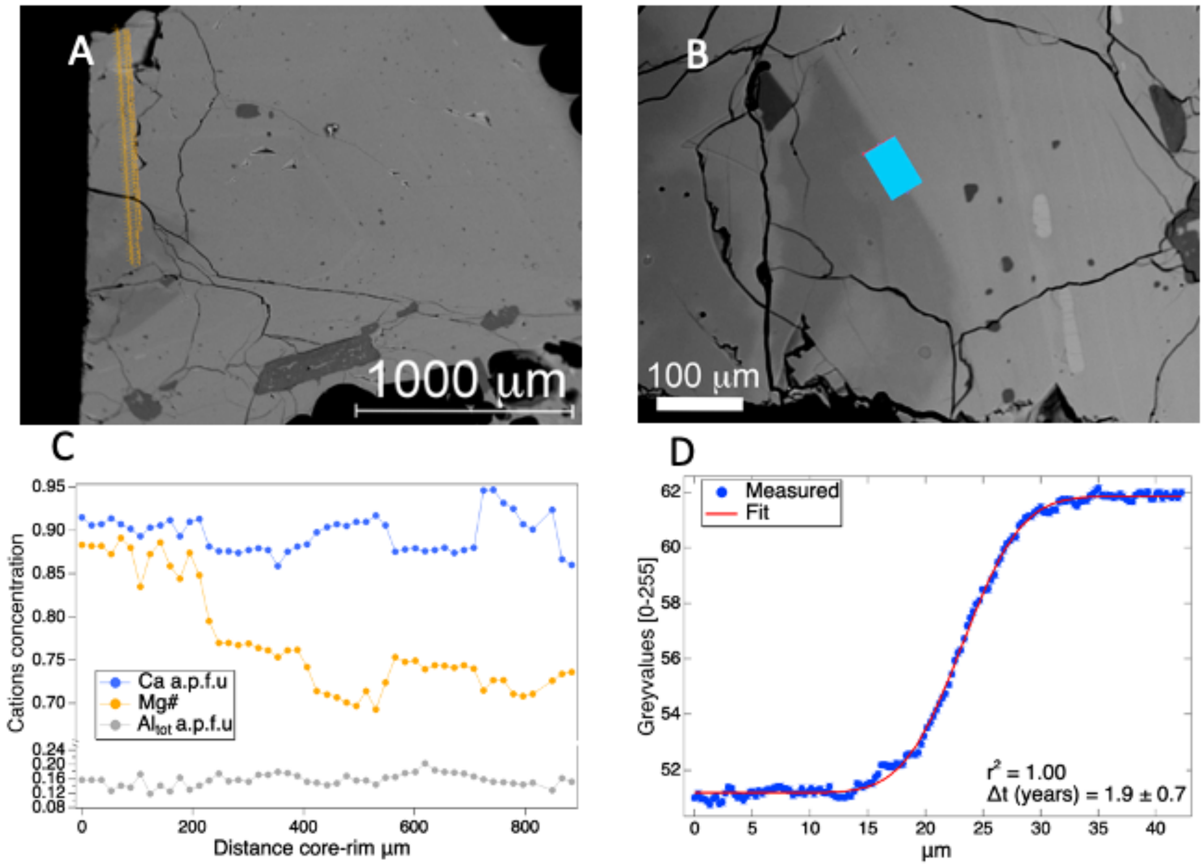

Supplementary Fig. 64.

Clinopyroxene chemical profile and diffusion modeling. (A) SEM-BSE image of the analyzed clinopyroxene. The yellow line with numbers marks the analyzed chemical profile reported in C. (B) SEM-BSE high resolution image showing the area (light blue area) where the grey scale profile for the diffusion profile has been extracted using the *greyvalues* Matlab script of the NIDIS model from Petrone *et al.*[5]. The red dot on the light blue area marks the initial point of the profile. (C) Variation of Ca (blue dots +line), Al (grey dots + line) (a.p.f.u.) and Mg# (orange dots + line) *vs* distance (μm) along the chemical profile shown in (A). (D) Grey values diffusion profile (blue dots with error bar) *vs* distance (μm) extracted along the light blue area in (B). The red line is the fit of the diffusion modeling calculated using the *createfit* Matlab script of the NIDIS model from Petrone *et al.*[5]. The goodness of the fitting is reported as r^2 and Δt is the calculated timescales (in days or years). The grey values are used as proxy of Mg# in clinopyroxene following Petrone *et al.*[5]. The temperature at which the diffusion has been calculated is reported in Table 1 for each clinopyroxene. See Method and Materials for further details.

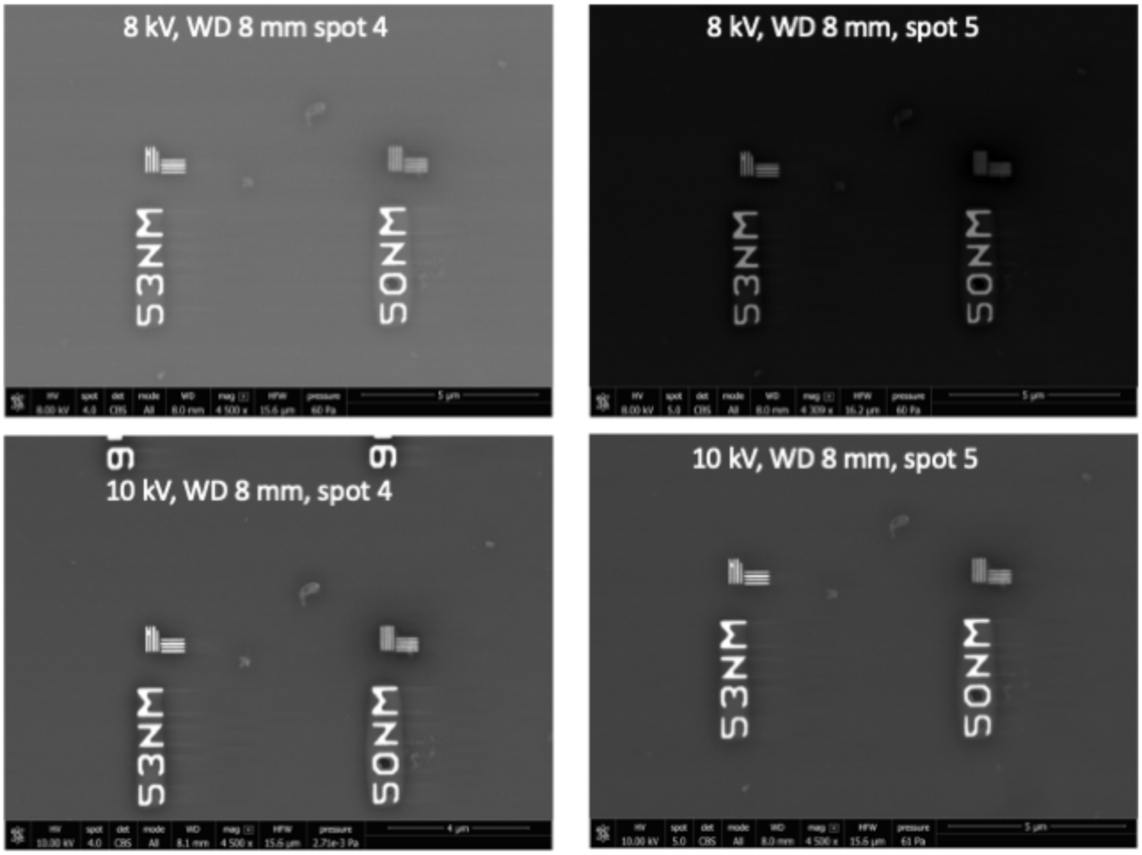

Supplementary Fig. 65.
Back-Scattered Electron (BSE) SEM images of the Richardson Test Slide (model 80302, Serial n. 10461) showing the resolution of the FEI Quanta 650 FEG-SEM operating at different voltages (8, 10 kV), working distance (WD) of 8 mm and spot size 4 and 5.

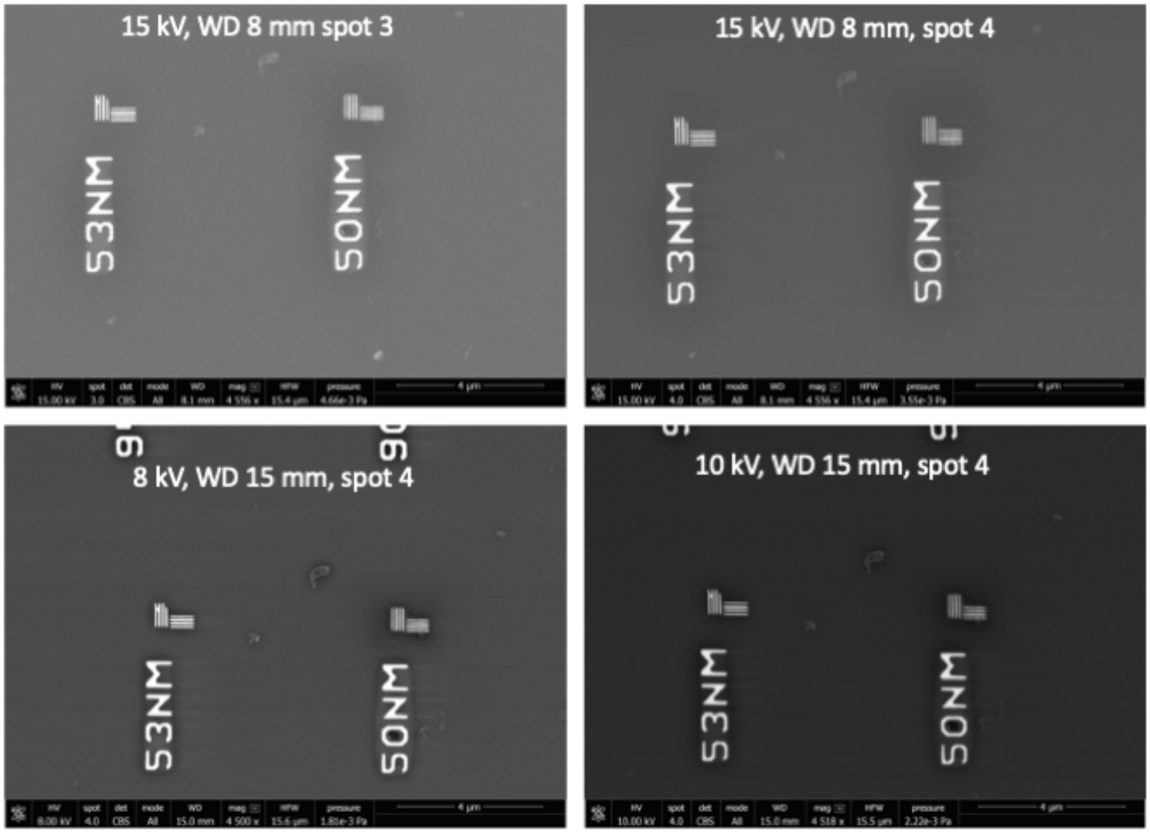

Supplementary Fig. 66.
Back-Scattered Electron (BSE) SEM images of the Richardson Test Slide (model 80302, Serial n. 10461) showing the resolution of the FEI Quanta 650 FEG-SEM operating at different voltages (8, 10, 15 kV), working distance (WD) of 8 and 15 mm and spot size 3 and 4.

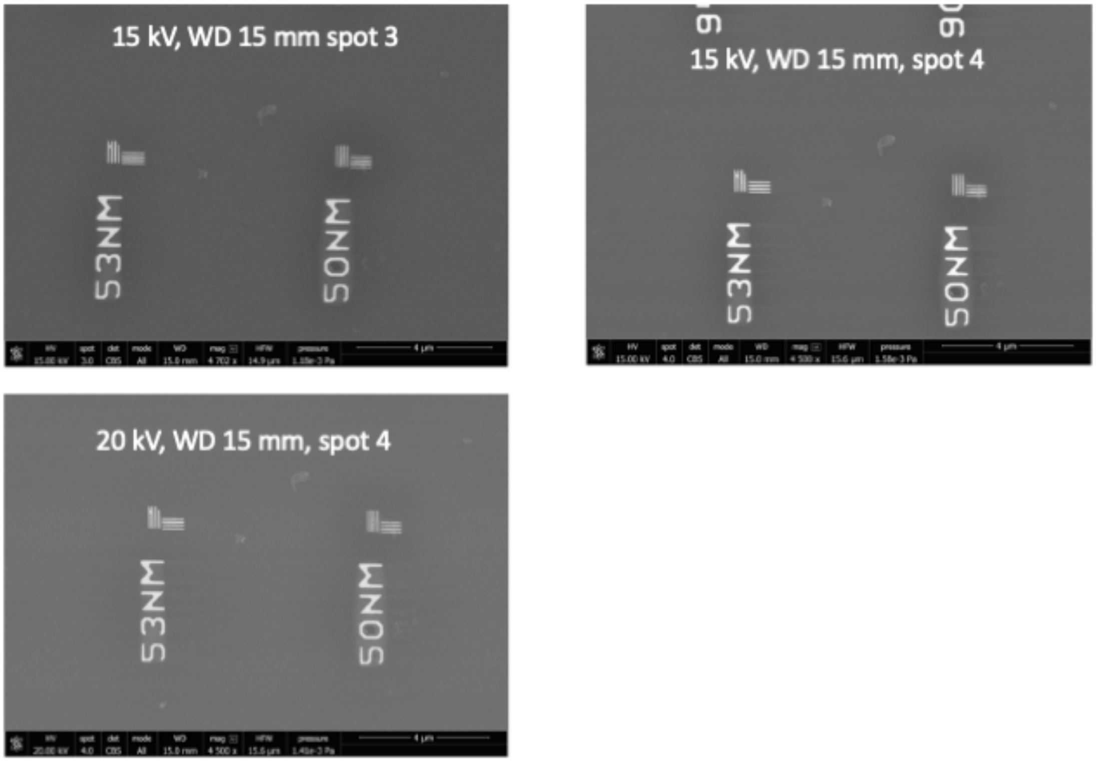

Supplementary Fig. 67.
Back-Scattered Electron (BSE) SEM images of the Richardson Test Slide (model 80302, Serial n. 10461) showing the resolution of the FEI Quanta 650 FEG-SEM operating at different voltages (15, 20 kV), working distance (WD) of 15 mm and spot size 3 and 4.

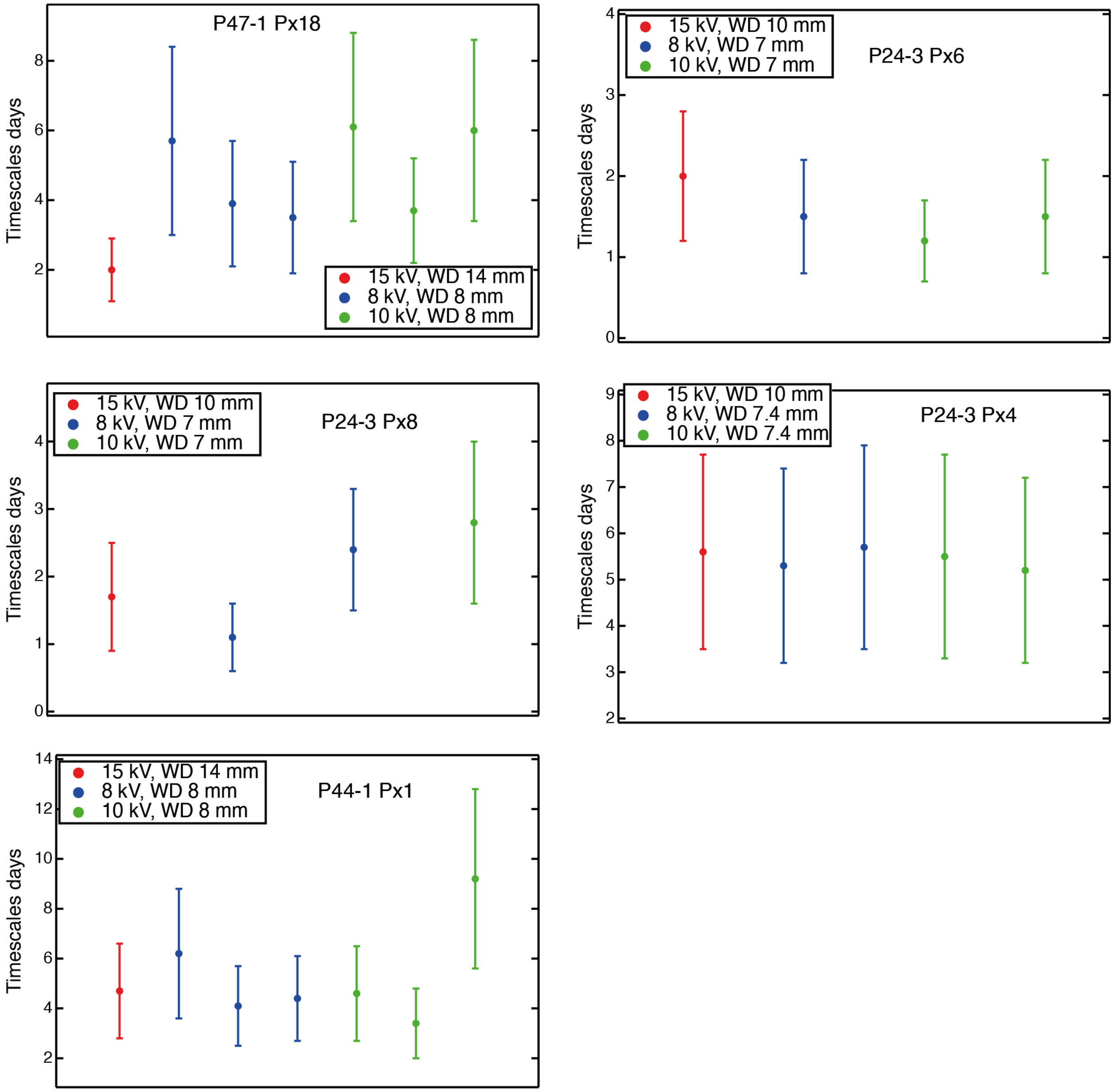

Supplementary Fig. 68.

Comparison of timescales calculated fitting greyscales profiles extracted form BSE-SEM images taken operating the FEG-SEM at different voltages and working distance conditions to test the convolution effect on the extracted greyscale profiles.

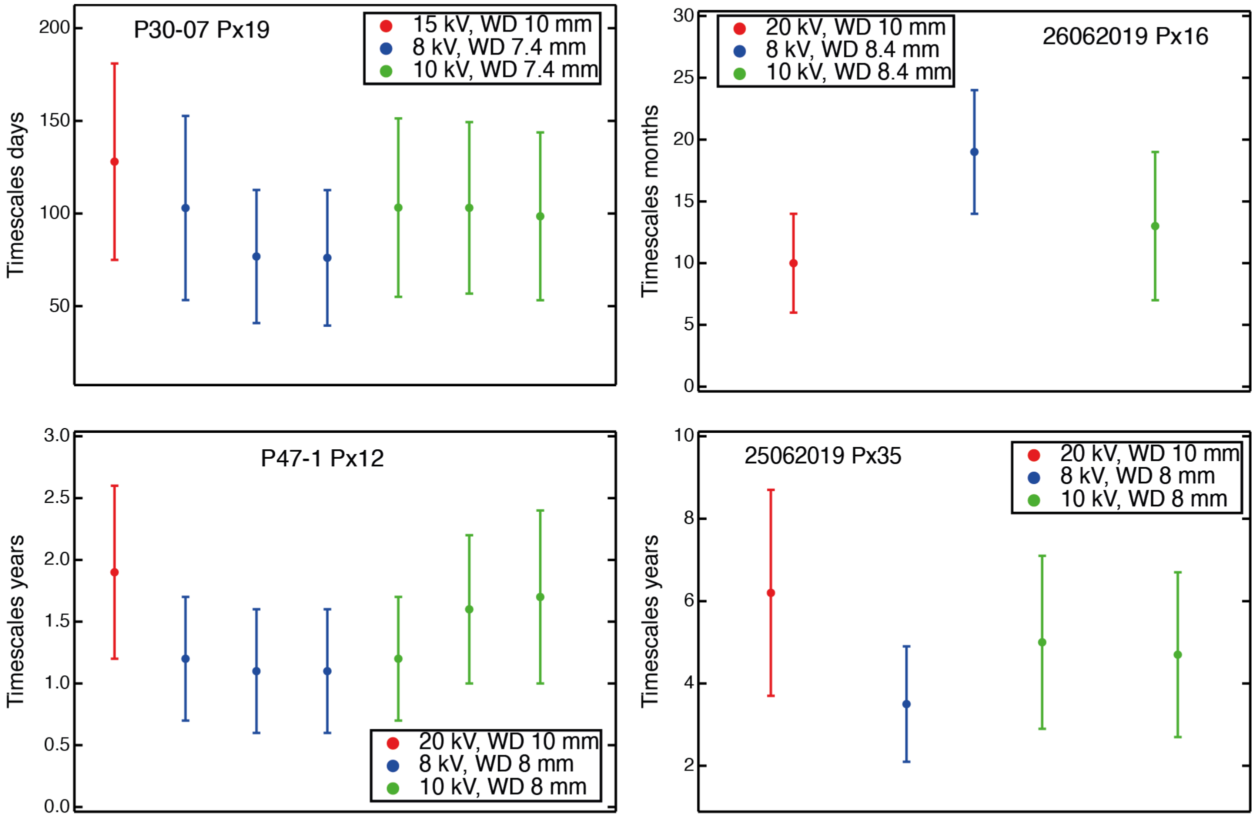

Supplementary Fig. 69.

Comparison of timescales calculated fitting greyscales profiles extracted form BSE-SEM images taken operating the FEG-SEM at different voltages and working distance conditions to test the convolution effect on the extracted greyscale profiles.

References

1. Francalanci, L., Lucchi, F., Keller, J., De Astis, G. & Tranne, C. A. Eruptive, volcano-tectonic and magmatic history of the Stromboli volcano (north-eastern Aeolian archipelago). *Geol. Soc. Mem.* **37**, 397–471 (2013).
2. Di Stefano, F. *et al.* Mush cannibalism and disruption recorded by clinopyroxene phenocrysts at Stromboli volcano: New insights from recent 2003–2017 activity. *Lithos* **360–361**, 105440 (2020).
3. Mollo, S. *et al.* A review of the lattice strain and electrostatic effects on trace element partitioning between clinopyroxene and melt: Applications to magmatic systems saturated with Tschermak-rich clinopyroxenes. *Earth-Science Rev.* **210**, 103351 (2020).
4. Mollo, S., Putirka, K., Misiti, V., Soligo, M. & Scarlato, P. A new test for equilibrium based on clinopyroxene–melt pairs: Clues on the solidification temperatures of Etnean alkaline melts at post-eruptive conditions. *Chem. Geol.* **352**, 92–100 (2013).
5. Petrone, C. M., Bugatti, G., Braschi, E. & Tommasini, S. Pre-eruptive magmatic processes re-timed using a non-isothermal approach to magma chamber dynamics. *Nat. Commun.* **7**, 12946 (2016).
