# Supplementary material for: Magma recharge and mush rejuvenation drive paroxysmal activity at Stromboli volcano
Source: Nat Commun. 2022 Dec 13;13:7717. doi: 10.1038/s41467-022-35405-z (PMC9746564; doi:10.1038/s41467-022-35405-z)
Supplement: Supplementary file 2 — Description of Additional Supplementary files [file 41467_2022_35405_MOESM2_ESM.pdf]

Description of Additional Supplementary Files

File name: Supplementary Data 1
Description: Bulk rock analyses of Stromboli eruptions

File name: Supplementary Data 2
Description: Major oxide concentrations of 2019 glasses

File name: Supplementary Data 3
Description: Major oxide concentrations of 2019 clinopyroxene crystals

File name: Supplementary Data 4
Description: Trace element concentrations of 2019 clinopyroxene crystals

File name: Supplementary Data 5
Description: Quality control of measured against certified standards used for bulk rock analyses at the Activation Laboratories Ltd. (Actlabs)

File name: Supplementary Data 6
Description: Quality control of measured against certified standards used for mineral analyses at the NHM-IAC laboratories
